# Improving wood properties for wood utilization through multi-omics integration in lignin biosynthesis

**DOI:** 10.1038/s41467-018-03863-z

**Published:** 2018-04-20

**Authors:** Jack P. Wang, Megan L. Matthews, Cranos M. Williams, Rui Shi, Chenmin Yang, Sermsawat Tunlaya-Anukit, Hsi-Chuan Chen, Quanzi Li, Jie Liu, Chien-Yuan Lin, Punith Naik, Ying-Hsuan Sun, Philip L. Loziuk, Ting-Feng Yeh, Hoon Kim, Erica Gjersing, Todd Shollenberger, Christopher M. Shuford, Jina Song, Zachary Miller, Yung-Yun Huang, Charles W. Edmunds, Baoguang Liu, Yi Sun, Ying-Chung Jimmy Lin, Wei Li, Hao Chen, Ilona Peszlen, Joel J. Ducoste, John Ralph, Hou-Min Chang, David C. Muddiman, Mark F. Davis, Chris Smith, Fikret Isik, Ronald Sederoff, Vincent L. Chiang

**Affiliations:** 1 State Key Laboratory of Tree Genetics and Breeding, Northeast Forestry University, Harbin, 150040 China; 2 Forest Biotechnology Group, Department of Forestry and Environmental Resources, North Carolina State University, Raleigh, NC 27695 USA; 3 Electrical and Computer Engineering, North Carolina State University, Raleigh, NC 27695 USA; 4 State Key Laboratory of Tree Genetics and Breeding, Chinese Academy of Forestry, Beijing, 100091 China; 5 Joint BioEnergy Institute, Lawrence Berkeley National Laboratory, Emeryville, CA 94608 USA; 6 Civil, Construction and Environmental Engineering, North Carolina State University, Raleigh, NC 27695 USA; 7 Department of Forestry, National Chung-Hsing University, Taichung, 40227 Taiwan; 8 Department of Chemistry, W.M. Keck Fourier Transform Mass Spectrometry Laboratory, North Carolina State University, Raleigh, NC 27695 USA; 9 School of Forestry and Resource Conservation, National Taiwan University, Taipei, 10617 Taiwan; 10 Department of Biochemistry, and the DOE Great Lakes Bioenergy Research Center, Wisconsin Energy Institute, University of Wisconsin, Madison, WI 53726 USA; 11 National Bioenergy Center, National Renewable Energy Laboratory, Golden, CO 80401 USA; 12 Department of Forest Biomaterials, North Carolina State University, Raleigh, NC 27695 USA; 13 Department of Operations Research, North Carolina State University, Raleigh, NC 27695 USA; 14 Department of Forestry, Beihua University, Jilin, 132013 China; 15 Department of Life Sciences, College of Life Science, National Taiwan University, Taipei, 10617 Taiwan; 16 Bioinformatics Research Center, North Carolina State University, Raleigh, NC 27695 USA; 17 Department of Forestry and Environmental Resources, North Carolina State University, Raleigh, NC 27695 USA

## Abstract

A multi-omics quantitative integrative analysis of lignin biosynthesis can advance the strategic engineering of wood for timber, pulp, and biofuels. Lignin is polymerized from three monomers (monolignols) produced by a grid-like pathway. The pathway in wood formation of *Populus trichocarpa* has at least 21 genes, encoding enzymes that mediate 37 reactions on 24 metabolites, leading to lignin and affecting wood properties. We perturb these 21 pathway genes and integrate transcriptomic, proteomic, fluxomic and phenomic data from 221 lines selected from ~2000 transgenics (6-month-old). The integrative analysis estimates how changing expression of pathway gene or gene combination affects protein abundance, metabolic-flux, metabolite concentrations, and 25 wood traits, including lignin, tree-growth, density, strength, and saccharification. The analysis then predicts improvements in any of these 25 traits individually or in combinations, through engineering expression of specific monolignol genes. The analysis may lead to greater understanding of other pathways for improved growth and adaptation.

## Abstract (web summary)

A systematic analysis of lignin biosynthetic genes to quantitatively understand their effect on wood properties is still lacking. Here, the authors integrate transcriptomic, proteomic, fluxomic and phenomic data to quantify the impact of perturbations of transcript abundance on lignin biosynthesis and wood properties.

## Introduction

**Fig. 1:**
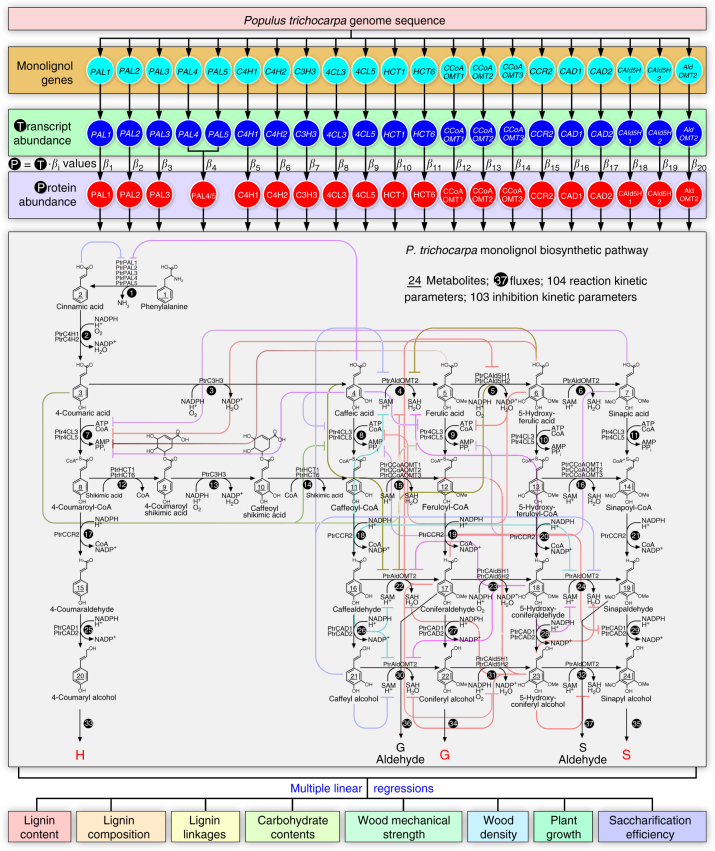
Our multi-omics integrative analysis of the monolignol biosynthetic pathway in *P. trichocarpa* predicts wood properties for wood utilization. The pathway is represented by 21 monolignol genes (turquoise circles) identified from the genome sequence, and their corresponding transcripts (blue circles) and proteins (red circles). The gene transcript abundances can be converted to protein abundances by specific *β*_i_ values from linear regression models (Supplementary Table 2). The monolignol proteins in a metabolic grid convert phenylalanine to monolignols in stem-differentiating xylem. The metabolic grid consists of 24 metabolites (underlined numbers within structures), 37 reaction fluxes (white numbers on black circles), 104 reaction kinetic parameters and 103 inhibition kinetic parameters. Other enzymes, regulators, and components are involved but genes encoding these factors are unknown or not yet sufficiently characterized to be included in this study. Fluxes 16, 20, 21, and 26 were set to zero because kinetic parameters for these reactions are not currently available. Colored lines represent pathway enzyme inhibitions. Multiple linear regressions predict the lignin and wood properties. Abbreviations: Reduced nicotinamide adenine dinucleotide phosphate (NADPH), oxidized nicotinamide adenine dinucleotide phosphate (NADP+), coenzyme A (CoA), adenosine triphosphate (ATP), adenosine monophosphate (AMP), pyrophosphate (PPi), S-adenosylmethionine (SAM), S-adenosylhomocysteine (SAH)

Wood has long been used as construction material and for pulp and paper production. Such uses are in considerable part determined by the properties of lignin in wood. Removal of lignin is the key step in pulp and paper production and the conversion of biomass to liquid biofuels. Lignin was discovered in wood by Anselme Payen in 1839 as the incrusting material that must be removed to isolate wood’s useful fiber, cellulose^1^. Lignin is a major phenolic polymer in plant secondary cell walls and is formed predominately from the oxidative polymerization of three major monolignols, 4-coumaryl alcohol (H-subunit, 20, Fig. 1), coniferyl alcohol (G-subunit, 22), and sinapyl alcohol (S-subunit, 24)^2^ in angiosperms. The monolignols polymerize around a framework of cellulose and hemicelluloses to form secondary cell walls in trees to make wood. Despite more than half a century of investigation, there is not yet a fundamental quantitative basis for understanding the regulation of lignin biosynthesis and its effects on the properties and utilization of wood.

**Fig. 2:**
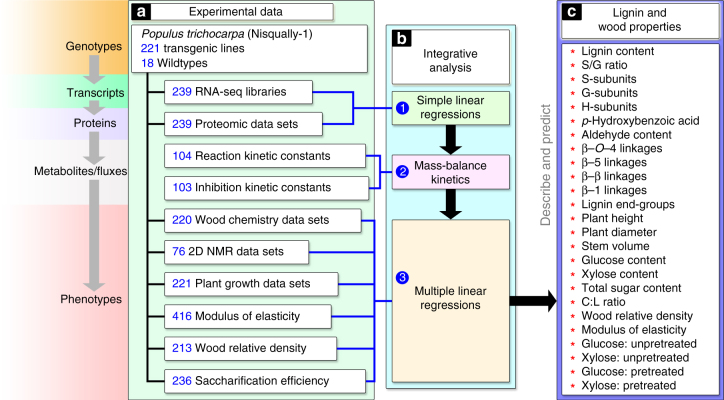
Systematic integrative analysis of lignin biosynthesis in *P. trichocarpa*. **a** The experimental data measured for the transgenics and wildtype. **b**(1-3) The quantitative relationships between transcript and protein abundances are represented by simple linear regressions. Monolignol pathway metabolites and fluxes are estimated using mass-balance kinetics. Multiple linear regressions associate the metabolite concentrations and fluxes with lignin and wood properties. **c** The 25 key lignin and wood properties

We now present a quantitative multi-omics integration of the regulation of lignin biosynthesis in wood formation in the model woody plant *Populus trichocarpa* (Torr. & Gray) that improves wood properties for utilization. The genome sequence of *P. trichocarpa*^3^ identified the genes for the biosynthesis of lignin from its monolignol precursors^4^. A core set of 21 genes^4^ (known at the onset of this study) encode specific enzymes that mediates a biosynthetic pathway of 37 metabolic-fluxes converting phenylalanine (1, Fig. 1) through 24 intermediate metabolites into the three monolignols (20, 22, and 24)^5^ for lignin formation that determines wood properties. Using transgenic *P. trichocarpa*, we systematically integrated five levels of regulation (genomic, transcriptomic, proteomic, fluxomic, and phenomic, Fig. 1, Fig. 2a, b) to quantify the effects of the expression of monolignol biosynthetic pathway genes on wood properties and wood utilization, such as saccharification (extraction of sugars) (Fig. 2c).

## Results

### Overall approach

The five level multi-omics integrative analysis involves three major steps (1–3, Fig. 2b). (1) Quantifying the relationships between the abundances of gene-specific transcripts and proteins. (2) Establishing the effects of gene-specific pathway protein abundances on metabolic-flux and metabolite concentrations. (3) Quantifying the effects of predicted metabolic-flux and metabolite concentrations on specific end products (lignin and other wood properties, Fig. 2c). We defined these relationships through xylem-specific transgenic perturbation of the 21 monolignol pathway genes (Fig. 2a, Supplementary Table 1; Supplementary Data 1) to provide a broad range of variation in the levels of gene expression and the downstream responses. Many of these genes are phylogenetically paired members within different gene families^4^. We therefore used RNA interference constructs (types I–III, Supplementary Fig. 1a) to downregulate individual genes, gene-pairs or gene families. Artificial microRNA constructs (type IV, Supplementary Fig. 1a) were also designed to target the specific downregulation of single genes within gene families. We generated ~2000 transgenic *P. trichocarpa* trees and selected 221 independent lines showing varying degrees of gene downregulation (Supplementary Table 1, Supplementary Data 1, Supplementary Note).

Monolignol biosynthetic genes are abundantly expressed in both fiber and vessel cells (the key wood-forming cells; Supplementary Fig. 1b) in stem-differentiating xylem. We isolated the stem-differentiating xylem from the transgenics and wildtype and analyzed 239 full transcriptomes and 239 proteomes to regress the abundances of transcripts and proteins (Fig. 2a, b). Using recombinant proteins from the 21 monolignol pathway genes used in this study, we determined 207 reaction and inhibition enzyme kinetic parameters to predict the effects of protein abundances on pathway metabolic-fluxes and metabolite concentrations (Fig. 2a, b, Supplementary Data 9)^5–8^. To determine the effects of metabolic-fluxes and metabolite concentrations on lignin and wood properties, we quantified the chemical composition of 220 wood samples, and 76 lignin samples using 2D HSQC NMR for lignin composition and structures (Fig. 2a, b, Supplementary Note describes the number of samples used for each quantification). We measured the growth of 221 lines, the modulus of elasticity (MOE) of 416 wood samples, the density of 213 wood samples, and tested 236 wood samples for saccharification efficiency (Fig. 2a, b, Supplementary Note). All these data were then systematically integrated to describe the transduction of biological information from the 21 monolignol genes through transcripts, proteins, metabolic-fluxes, and metabolite concentrations, leading to specific lignin and wood properties (Fig. 2a–c).

### Monolignol gene transcript and protein relationships

**Fig. 3:**
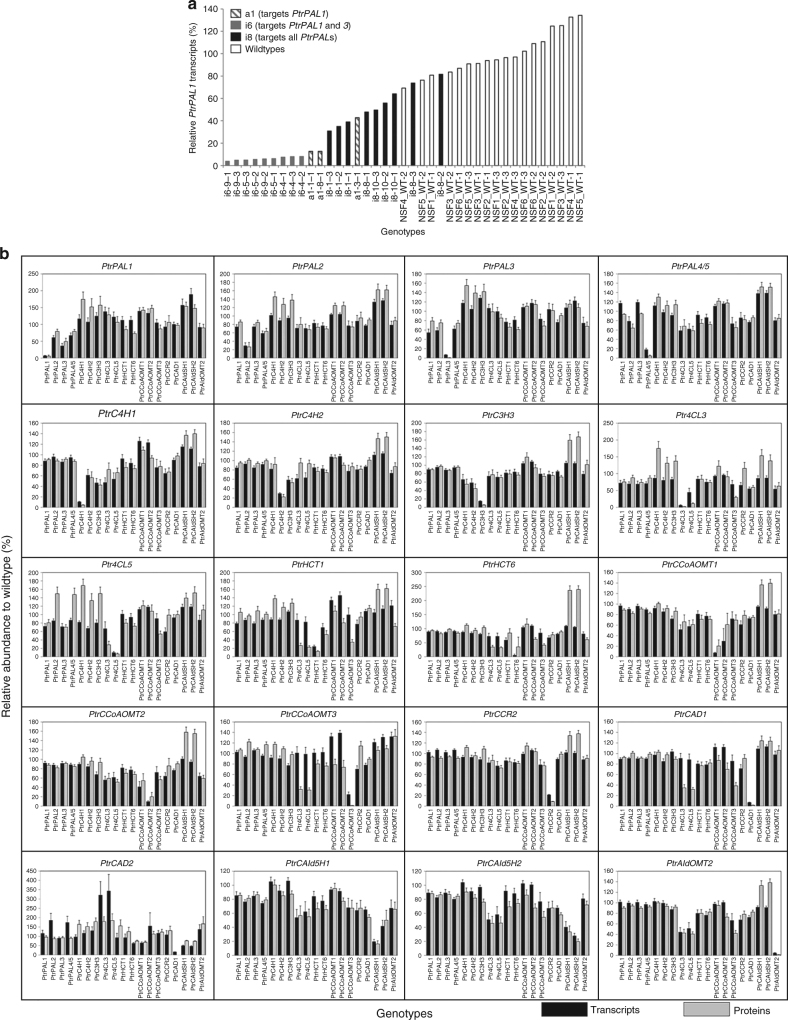
The abundance of the monolignol biosynthetic gene transcripts and proteins in transgenic *P. trichocarpa* lines. **a** Relative abundance of *PtrPAL1* transcripts in the transgenic and wildtype *P. trichocarpa*. White bars represent *PtrPAL1* transcript abundance in wildtype trees. Striped bars represent *PtrPAL1* transcript abundance in transgenic lines targeting the downregulation of *PtrPAL1*. Gray bars represent *PtrPAL1* transcript abundance in transgenic lines targeting the downregulation of both *PtrPAL1* and *PtrPAL3*. Black bars represent *PtrPAL1* transcript abundance in transgenic lines targeting the downregulation of all five *PtrPAL*s. **b** The *x*-axis represents the genotypes of the transgenic lines, and the *y*-axis represents relative transcript (black bars) and protein (gray bars) abundance to the average wildtype level. Error bars represent the standard error of at least three biological replicates

We analyzed the whole transcriptomes of the stem-differentiating xylem of 221 transgenics and 18 wildtypes by RNA-seq (GEO accession number: GSE78953) (Supplementary Fig. 1c, d). Transgenic suppression provided broad variation in the levels of monolignol gene expression (Supplementary Fig. 2). Variation in gene expression arose from (1) targeted transgene suppression, (2) indirect (non-targeted) effects of other monolignol transgenes, and (3) seasonal and environmental effects (Supplementary Note, Supplementary Fig. 3). Targeted transgenesis always resulted in a wide range of target gene suppression (Fig. 3a, Supplementary Data 1). For example, the targeted transgenic suppression of *PtrPAL1* expression ranged from 18.4 (i8-8-2, Fig. 3a) to 92.5% (i6-9-3) of the parental wildtype level. *PtrPAL1* was also suppressed by homologous transgenes of its family members, such as *PtrPAL3* (Fig. 3b), and to a lesser extent, by transgenes of members from other families, such as *PtrAldOMT2* (Fig. 3b), through gene interactions. The indirect effect of genetic interactions was observed for many monolignol genes, suggesting unknown higher-order regulation (Fig. 3b). Seasonal and environmental effects in the greenhouse (Supplementary Note) also contributed to variations in transcript abundance, as seen for *PtrPAL1* expression in the wildtypes (white bars, Fig. 3a). These variations provided a broad range of gene expression to correlate transcript abundance and protein abundance, and increased the power of the integrative analysis.

**Fig. 4:**
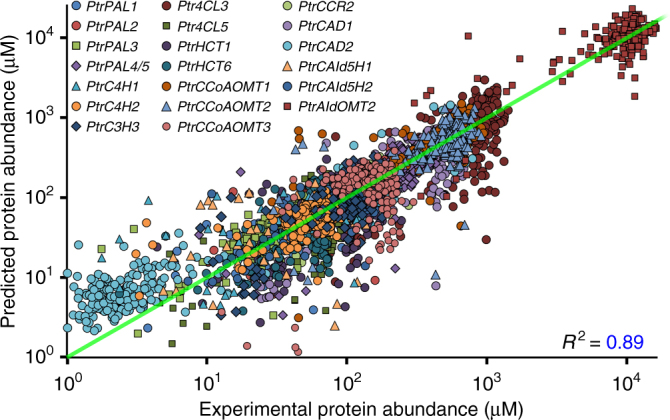
Regression of monolignol transcript/protein relationships. Transcript abundance is used in the linear regression equations (Supplementary Table 2) to obtain the predicted protein abundance (*y*-axis), which is regressed on the experimental protein abundance (*x*-axis). The equations can explain ~89% of the variation in abundance of the monolignol biosynthetic proteins in the 239 transgenic and wildtype samples, based on the abundance of the gene transcripts. Each data point represents the abundance of a monolignol gene for a replicate pool of one wildtype or transgenic line containing 3–5 clonally propagated plants

We determined the absolute quantities of the monolignol biosynthetic proteins in the stem-differentiating xylem of the 239 transgenics and wildtype using protein cleavage-isotope dilution mass spectrometry (PC-IDMS) and protein-specific stable-isotope-labeled internal standards, following Shuford et al. (2012)^9^. Specific reduction of protein abundance was observed in the downregulated transgenics for all monolignol pathway enzymes (Fig. 3b). Protein and transcript abundance for specific monolignol genes showed significant positive linear relationships (Fig. 4, Supplementary Fig. 2, Supplementary Table 2). We assembled a simple linear regression equation for each monolignol gene to describe the efficiency of translation of the transcript to the protein (equations 1–20, Supplementary Data 2). On average, each transcript molecule (inferred from RNA-seq, Supplementary Note) produces a steady-state level of ~1.5 × 10^4^ molecules of the protein (*β*_i_ values, Supplementary Table 2). Translation efficiencies are typically ~10^4^ in eukaryotes and ~540 in prokaryotes^10,11^. Different monolignol genes showed dramatically different efficiencies (*β*_i_ values, Supplementary Table 2, Supplementary Note). The monolignol gene transcript abundance explained on average 31% (coefficient of determination, *R*^2^ = 0.31) of the variation in protein abundance, which ranged from 53% (for *PtrPAL2*) to 3% (for *PtrCCoAOMT3*, a poor predictor) (Supplementary Table 2, Supplementary Note). The protein abundances predicted by the linear regressions were used in mass-balance equations to estimate metabolite concentrations and pathway reaction fluxes (Fig. 2b).

### Metabolic fluxes for monolignol biosynthesis

We previously measured 104 Michaelis–Menten kinetic parameters and 103 inhibition kinetic parameters for purified functional recombinant proteins of 21 monolignol genes^5,7^. These 207 kinetic parameters provide an approximation of the kinetic behaviors of the monolignol enzymes in vivo. We integrated these 207 kinetic parameters and the absolute quantity of monolignol proteins in stem-differentiating xylem into 39 mass-balance equations to predict the effects of changing the abundance of each pathway protein on metabolic-flux, total lignin content, and lignin S/G ratio^5,7^. We now extend these mass-balance equations (equations 21–59, Supplementary Data 2) to include the additional regulation that comes from some specific protein–protein interactions^12^ (Methods) and the transcript/protein regression equations (equations 1–20) to illustrate quantitatively how changes in the pathway gene transcript levels affect protein abundance, metabolite concentrations, metabolic-flux, and additional major lignin properties. These new properties include the abundances of the major lignin subunits (H, G and S) and their structural linkages, as well as two non-monolignol phenolics in lignin, based on semi-quantitative two-dimensional nuclear magnetic resonance (2D NMR) of lignin from transgenic trees. The non-monolignol phenolics are *p*-hydroxybenzoic acid and hydroxycinnamaldehydes (Fig. 2c, Fig. 1), which are usually present in minor quantities in lignin of wildtype *P. trichocarpa*. In transgenics modified in the expression of specific monolignol genes, the abundance of some of these non-monolignol phenolics incorporated into lignin is elevated (Supplementary Note) and affects wood properties (Supplementary Data 3–8).

Our current 59 equations (equations 1–59, Supplementary Data 2) describe how changes in monolignol transcript abundance affect protein abundances and the 37 metabolic-fluxes and 24 metabolite concentrations in the pathway (Fig. 1). We next characterized 25 major lignin and wood chemical and physical properties (Fig. 2c; and described in sections below) in the transgenics and wildtype (Supplementary Table 1) for multiple linear regressions (equations 60–84, Supplementary Data 2, Methods) to predict the effects of pathway flux and metabolite concentrations on these wood properties. The final 84 equations (equations 1–84, Supplementary Data 2) represent the quantitative relationships linking monolignol genes to transcripts, proteins, predicted metabolic-flux, predicted metabolite concentrations, and 25 lignin and wood chemical and physical properties (Fig. 2a–c).

### Wood chemical properties

**Fig. 5:**
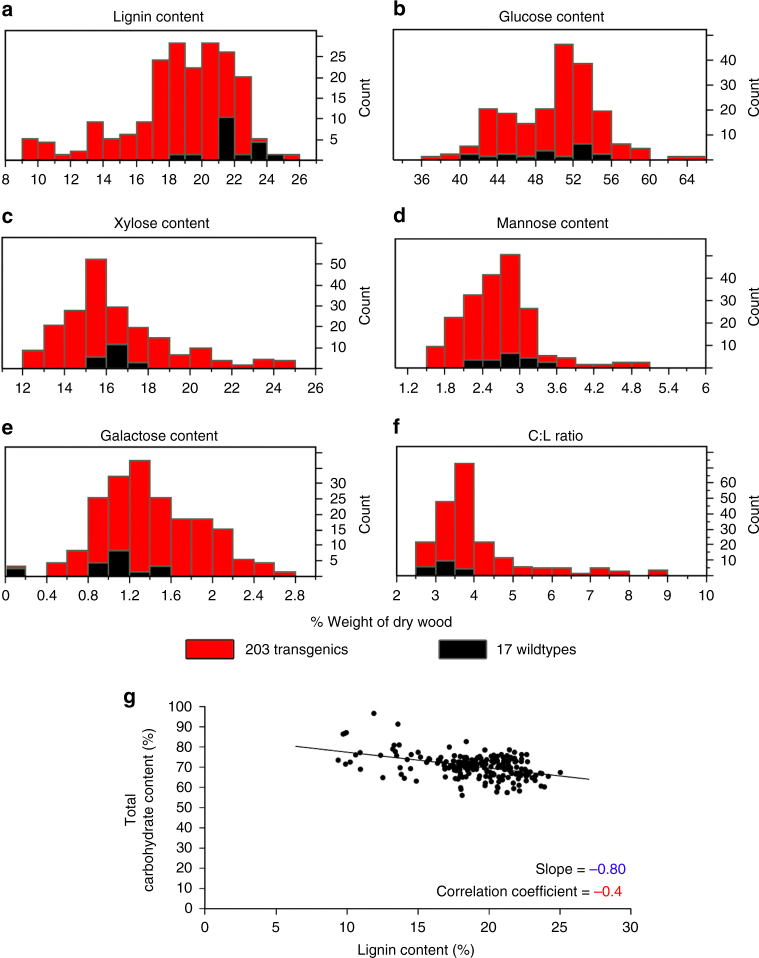
Variation of lignin and carbohydrate contents in transgenic and wildtype *P. trichocarpa*. **a** lignin, **b** glucose, **c** xylose, **d** mannose, and **e** galactose contents, and the carbohydrate to lignin (C:L) ratio **f** in the wood of transgenic (red bars) and wildtype (black bars) *P. trichocarpa*. See Supplementary Data 3. **g** Lignin and total carbohydrate contents are negatively correlated. The negative linear relationship has a slope of −0.8 and a correlation coefficient of −0.4. Each data point represents a replicate pool of one wildtype or transgenic line containing 3–5 clonally propagated trees

To quantify the effects of monolignol gene perturbations on wood chemical properties, we analyzed stem wood from 203 transgenics and 17 wildtypes. Wildtype wood contains on average 21.7% lignin, 49.8% glucose, 16.2% xylose, 2.8% mannose, and 1.0% galactose (% = g/100 g dry wood), with a total carbohydrate to lignin ratio (C:L) of 3.2 (Supplementary Data 3). The C:L ratio is a direct indicator of the potential maximum cellulosic yield for wood pulp and maximum sugar yield for biofuels and other bioproducts. The levels of monolignol gene expression significantly altered the wood composition; we observed broad variation of lignin content that ranged from 9.4 to 25.0% (Fig. 5a). Glucose varied from 37.0 to 66.9% (Fig. 5b), xylose from 12.4 to 23.9% (Fig. 5c), mannose from 0.0 to 4.9% (Fig. 5d), and galactose from 0.1 to 2.9% (Fig. 5e, Supplementary Data 3, Supplementary Fig. 4). The C:L ratios of the transgenics ranged from 2.5 to 8.8 (Fig. 5f), demonstrating the feasibility of generating raw materials suitable for a wide range of end uses. Consistent with our previous finding^13^, lignin content showed a significant negative linear relationship with total carbohydrate (Fig. 5g), having a slope of −0.8 and a correlation coefficient of −0.4. Therefore, for every 1% reduction in lignin, the total carbohydrate increases on average by ~0.8% (% = g/100 g dry wood).

**Fig. 6:**
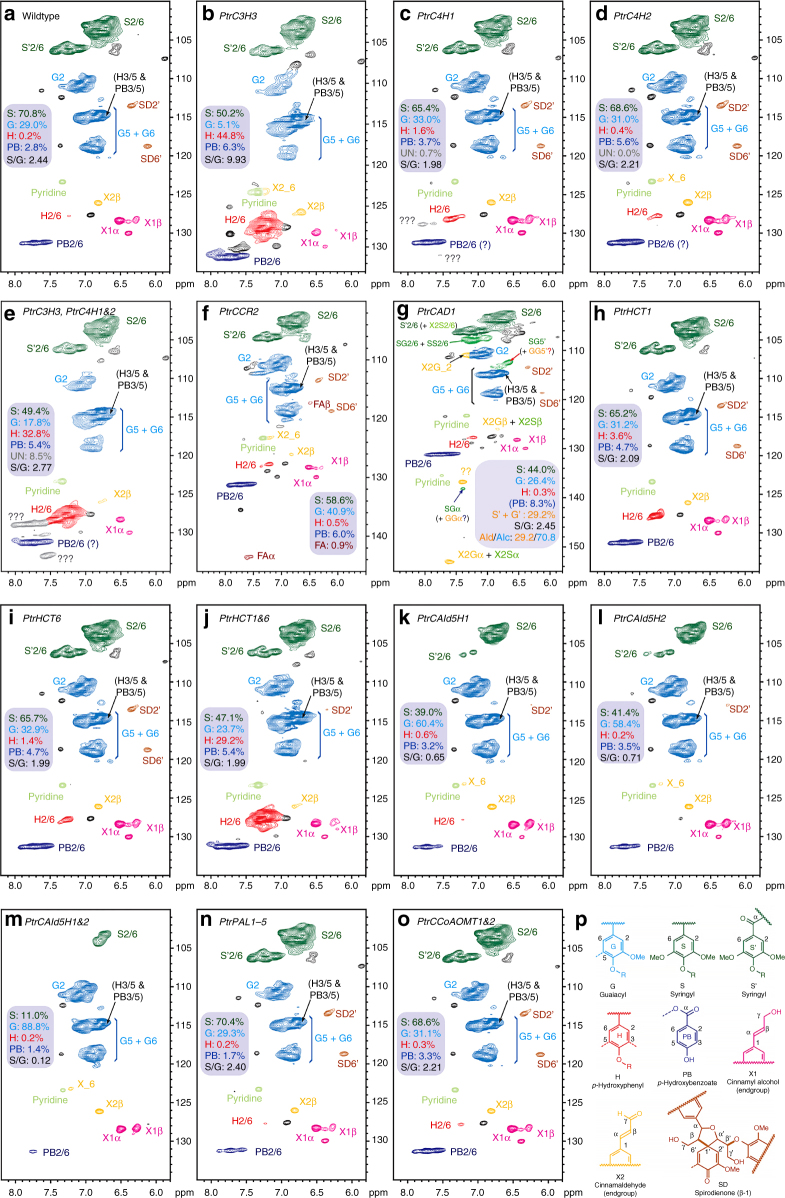
Composition of lignin quantified by 2D NMR. Partial (aromatic and double-bond regions) short-range 2D ^1^H–^13^C correlation (HSQC) spectra of enzyme lignin from wildtype (**a**), and an example of a transgenic *P. trichocarpa* line for each construct downregulated in the gene expression of **b** *PtrC3H3*, **c** *PtrC4H1*, **d** *PtrC4H2*, **e** *PtrC3H3* and *PtrC4H1*&*2*, **f** *PtrCCR2*, **g** *PtrCAD1*, **h** *PtrHCT1*, **i** *PtrHCT6*, **j** *PtrHCT1*&*6*, **k** *PtrCAld5H1*, **l** *PtrCAld5H2*, **m** *PtrCAld5H1*&*2*, **n** *PtrPAL1*–*5*, **o** *PtrCCoAOMT1*&*2*. S/G ratio and the percentage volume integrals of major lignin components are from contour volume integrals. **p** The chemical structures of the lignin monomeric subunits, color-coded to match their signal assignments in the spectra. See Supplementary Data 4 for all 2D NMR data

Lignin from 68 transgenics and 8 wildtypes was analyzed by 2D NMR to quantify lignin subunit composition and major interunit linkages (Fig. 6, Supplementary Data 4, Supplementary Fig. 5, Supplementary Fig. 6, Supplementary Note). Changing monolignol gene expression significantly altered lignin composition. In the lignin of transgenics, S-subunits ranged from 11.0 to 74.8%, G-subunits from 5.1 to 88.8%, H-subunits from 0.2 to 44.8%, *p*-hydroxybenzoic acid from 1.4 to 14.8%, and S/G ratios from 0.12 to 9.9 (Fig. 6b–o, Supplementary Data 4, Supplementary Note). The wide variation in lignin subunit composition also significantly altered the interunit linkages. β-Ethers (β–*O*–4-ether linkages) ranged from 76.9 to 95.9%, phenylcoumarans (β–5) from 1.2 to 16.3%, resinols (β–β) from 1.4 to 12.7%, spirodienones (β–1) from 0.0 to 3.5%, and cinnamyl alcohol end-groups from 4.6 to 14.2% (Supplementary Data 4, Supplementary Note).

### Wood physical properties

The consequences of changing monolignol gene expression on plant growth, wood mechanical strength, and density has not previously been systematically investigated in any tree species within a uniform genetic background. We measured the growth (height, diameter, and stem volume) of 221 transgenic and wildtype lines (6-month-old). Plant growth was significantly affected by the downregulation of specific monolignol genes (Supplementary Data 5). Transgenics downregulated in *PtrHCT*, *Ptr4CL* and *PtrCCR* families showed severe growth reduction (Supplementary Data 5) that does not correlate with lignin content. Some transgenics with low lignin, such as *PtrPAL* transgenics with 9.4% lignin (% = g/100 g dry wood) (i7-2-2, Supplementary Data 3) showed growth similar to wildtype (i7-2-2, Supplementary Data 5). Growth is also not associated with lignin subunit composition or specific linkages (Supplementary Data 5), but the growth reduction has been attributed to collapsed vessel elements^14^, transcriptional reprogramming^15^, and accumulation of chemical inhibitors^16^ in some transgenics.

The MOE is a quantitative index of wood mechanical strength and is widely used to guide solid wood and engineered wood utilization. A higher MOE indicates that the wood is stiffer and less prone to deformation. We measured MOE perpendicular to the longitudinal axis for stem segments of 416 transgenic and wildtype trees (Supplementary Data 6). Transgenic trees showed broad variation of MOE that ranged from 355 to 6058 Mega-Pascals (MPa), compared to the wildtype (3763 ± 121 MPa) (Supplementary Data 6). MOE is substantially affected by the subunit content and composition of lignin. All transgenic trees with reduced lignin showed proportional reductions in MOE (Supplementary Data 3 and 6). Under conditions where total lignin content does not change, an increase in hydroxycinnamaldehyde units from 4.0 to 29.2% in lignin (i33-05, Supplementary Data 4) resulted in a ~61% reduction in MOE (i33-05, Supplementary Data 6).

**Fig. 7:**
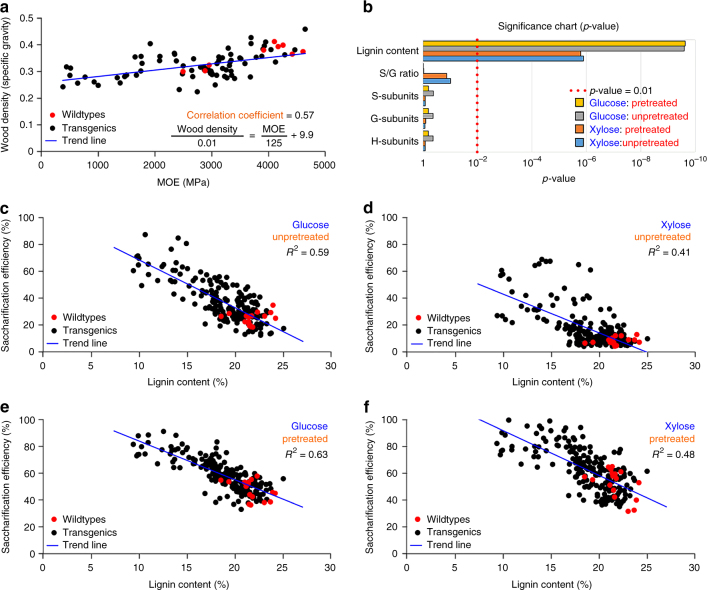
Quantitative relationships between wood relative density, MOE, lignin content, and saccharification efficiency. **a** The relationship between experimentally measured wood density and MOE. Each dot represents a replicate pool of one wildtype (red dots) or transgenic (black dots) line containing 3–5 clonally propagated plants. MOE and wood density show a positive linear relationship with a correlation coefficient of 0.57. Linear regression shows that each 0.01 increase in wood density corresponds to a 125 MPa increase in MOE. **b** Significance chart (*p*-value) shows that among the lignin properties, lignin content is the only significant effector (*p* < 0.01) of wood saccharification efficiency. The five highest *p*-values and the corresponding lignin properties are shown. **c**–**f** Wood saccharification efficiency is negatively correlated with lignin content. Scatterplots show the percentage release of glucose (**c**, **e**) and xylose (**d**,** f**) in the transgenic (black dots) and wildtype (red dots) *P. trichocarpa*. The wood samples were either unpretreated (**c**, **d**) or hot-water pretreated (**e**,** f**) (Methods). Each dot represents a replicate pool of one wildtype or transgenic line containing 3–5 clonally propagated plants. *R*^2^ denotes the coefficient of determination (percent variance explained by the regression)

Wood density (expressed as specific gravity, Supplementary Data 7) is one of the most important properties of wood because of its strong relationship to the yield and quality of wood products^17^. The density of 213 samples of transgenic and wildtype wood varied from 0.26 to 0.43 (wildtype = 0.36) (average density for each line, Supplementary Data 7). Wood density and mechanical strength (MOE) showed a positive correlation (coefficient = 0.57) (Fig. 7a). Each 0.01 unit increase in wood density corresponds to a 125 MPa increase in MOE (Fig. 7a). MOE per 0.01 unit increase in wood density for *P. cathayana* and *P. tomentosa* are 120 and 125 MPa, respectively, and averaged 166 MPa across 16 diverse woody plant species^18^.

### Saccharification efficiency and lignin

Wood is an attractive resource for sustainable biofuel and biomaterial production. However, the recalcitrant properties of lignin have impeded enzymatic saccharification for biofuels and bioproducts. Acid pretreatment is typically used to reduce lignin recalcitrance to facilitate enzymatic saccharification, but the process is costly and produces enzyme inhibitors^19^. Lowering lignin content reduces or eliminates the need for chemical pretreatment^20^. The saccharification efficiency of 236 wood samples of the transgenics and wildtype generated here was calculated from the quantities of glucose and xylose (the two main sugars in hardwood species) released from unpretreated or pretreated (Methods) wood samples (Fig. 7b–f). In unpretreated wildtypes, the saccharification efficiency of releasing glucose from glucans (cellulose and glucomannan) is 24.8%, and xylose from xylans is 8.3% (Fig. 7c, d, Supplementary Data 8). Unpretreated transgenic wood with reduced lignin showed high glucan saccharification efficiencies, up to 87.2% (Fig. 7c) and xylan up to 68.8% (Fig. 7d, Supplementary Data 8). Without pretreatment, every 1% (% = g/100 g dry wood) lignin content reduction results in a 3.5% increase in glucose saccharification efficiency (Fig. 7c) and a 2.8% increase in xylose efficiency (Fig. 7d). Pretreatment elevated the saccharification efficiencies of wildtype wood from 24.8 to 47.6% for glucose (Fig. 7e) and from 8.3 to 52.8% for xylose (Fig. 7f). Pretreatment together with transgenic reduction in lignin content further elevated wood saccharification efficiencies from 47.6 to 91.1% for glucose (Fig. 7e) and from 52.8 to 99.6% for xylose (Fig. 7f). The glucan and xylan saccharification efficiencies are strongly and negatively affected by lignin content whether the wood is pretreated or not (Fig. 7b–f), consistent with previous studies in alfalfa^21^ and Arabidopsis^22^. The efficiency in *P. trichocarpa* is not significantly affected by the lignin subunit composition or its linkage distribution (*p* > 0.01, Fig. 7b). Lignin and sugar composition have been shown to influence saccharification efficiency in Arabidopsis^22^ and unpretreated alfalfa^21^.

### Integrative systems analysis

**Fig. 8:**
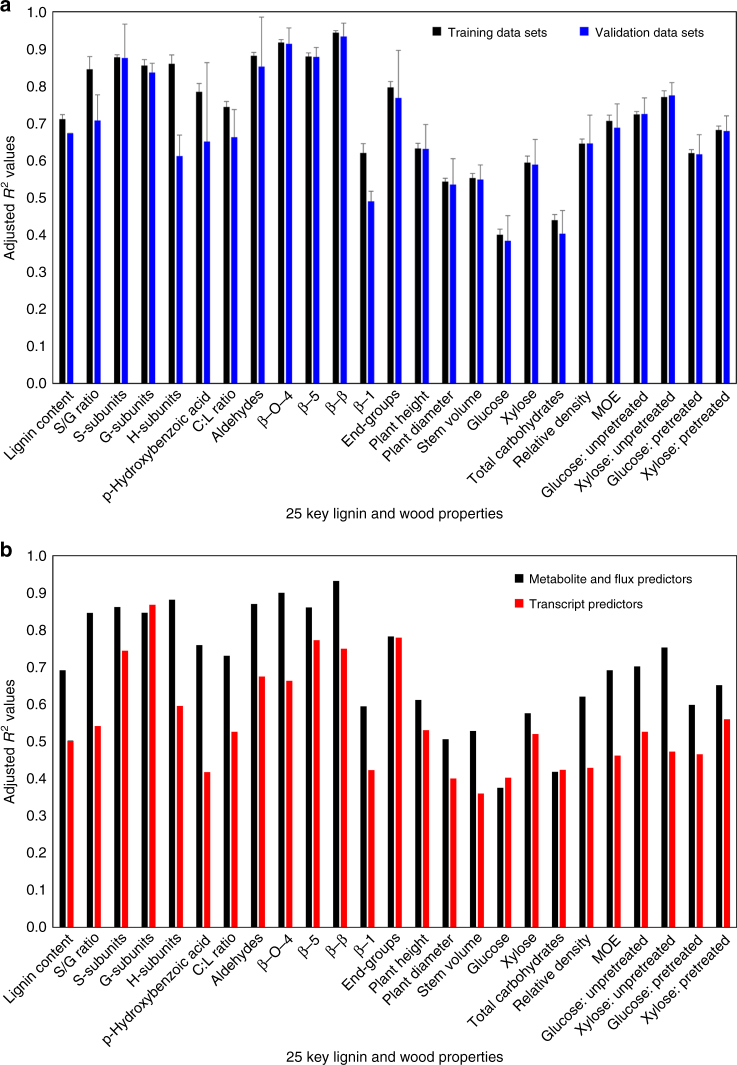
Integrative analysis validation. **a** Average adjusted *R*^2^ values for the training data sets (black bars) and the validation data sets (blue bars) for a five-fold cross-validation. Error bars denote the standard error of five sets of validations. **b** Adjusted *R*^2^ values for the lignin and wood properties predicted using either absolute transcript abundances as predictors (red bars) or metabolic-fluxes and metabolite concentrations as predictors (black bars). For both regression equations, an all-possible-model algorithm was used to determine the specific predictor variables. See Methods for detail. Adjusted *R*^2^ values represent variation explained by the regressions after adjusting for a different number of predictors

**Fig. 9:**
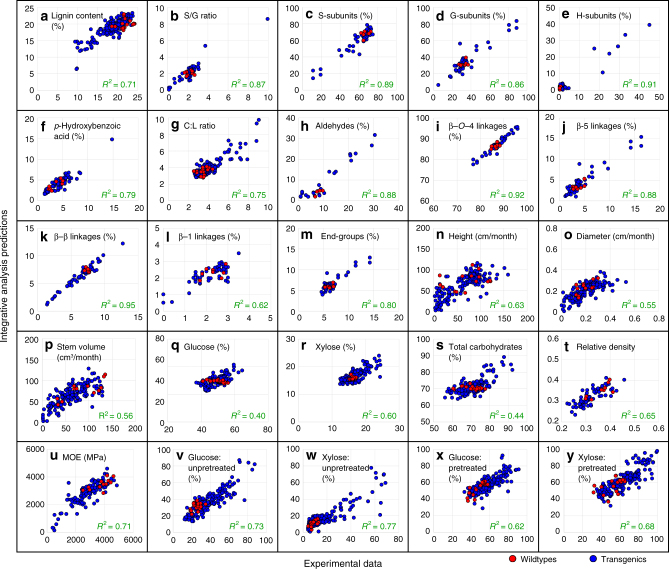
Scatterplots of the phenotypic variations captured by the lignin-based integrative analysis. The monolignol transcript abundance was used as the only input for the multi-level integrative analysis to predict the corresponding protein abundance, predicted metabolic-flux, predicted metabolite concentrations, and the 25 lignin and wood properties. The figure shows that transcript abundance is used to predict the 25 lignin and wood properties (*y*-axis, through the multi-level analysis), which are regressed on the experimental data (*x*-axis, measured in transgenics and wildtype). **a** Lignin content; **b** S/G ratio; **c** S-subunits; **d** G-subunits; **e** H-subunits; **f** *p*-Hydroxybenzoic acid; **g** Carbohydrate to lignin (C:L) ratio; **h** Aldehyde content; **i** β–*O*–4 linkages; **j** β–5 linkages; **k** β–β linkages; **l** β–1 linkages; **m** Cinnamyl alcohol end-groups; **n** Height; **o** Diameter; **p** Stem volume; **q** Glucose content; **r** Xylose content; **s** Total carbohydrates; **t** Relative density; **u** MOE; **v**–**y** Saccharification efficiency for glucose (**v**,** x**) and xylose (**w**, **y**) release from unpretreated (**v**, **w**) or pretreated (**x**,** y**) wood samples. The *R*^2^ values are coefficients of determination, showing the percent variation in dependent variables explained by monolignol gene transcript abundance as the predictor. Each data point represents a replicate pool of one wildtype or transgenic line containing 3–5 clonally propagated trees

We then systematically combined the individual quantitative effects of changing monolignol gene expression on protein quantity, predicted metabolic-flux, predicted metabolite concentration, lignin (content, subunit composition, and linkages), carbohydrates, growth, MOE, wood density, and saccharification efficiency for an integrative analysis (Fig. 2b). The analysis integrates 84 equations (Supplementary Datas 2 and 9, Methods), describing and predicting the behavior of the lignin biosynthetic pathway and the consequence of monolignol gene perturbations on the chemical and physical properties of wood. We validated the predictive capabilities of the analysis using five-fold cross-validation. The cross-validation includes re-estimation of all transcript/protein constants (*β*_i_ values) and multiple linear regression parameters in the absence of the validation data sets, thereby providing a validity check of the entire integrative analysis. The training data sets and the validation data sets (Fig. 8a) show similar *R*^2^ values, indicating that the integrative analysis equations are not overfitting the experimental data. With only transcript abundance as the input, the validated integrative analysis accurately captured the variation in lignin and wood properties of the transgenics and wildtype (Fig. 9). The integrative analysis explained on average 82% of the variation in lignin properties (Fig. 9a–m) and 72% for all 25 wood properties (Fig. 9a–y). Lower variations (*R*^2^ of 0.40 and 0.44, respectively) in glucose and total carbohydrates (Fig. 9q, s) were explained, indicating that these traits are more strongly regulated by mechanisms other than lignin biosynthesis. The analysis predictions and the experimentally measured values showed minimal bias (average bias = 0.96, Supplementary Table 3), suggesting that our integrative analysis has high predictive power. Quantitative estimation of the monolignol pathway fluxes and metabolite concentrations is necessary for the multiple linear regression equations to accurately predict lignin and wood properties (Fig. 8b, Supplementary Note).

### Strategic engineering of wood properties

**Fig. 10:**
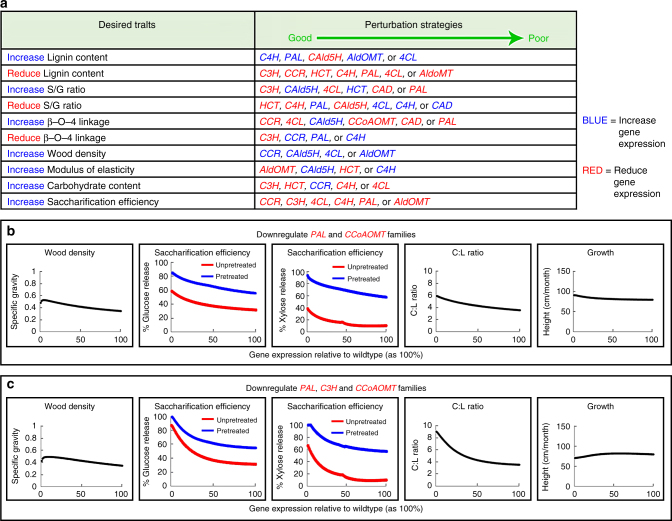
Strategic engineering of single or combinations of multiple lignin and wood traits through manipulating the expression of specific monolignol genes or combinations of these genes. **a** The integrative analysis based gene perturbation strategies to achieve specific desired wood traits. Perturbation strategies for each desired trait are arranged from the most significant effector to the least. Blue text represents increased gene expression, and red text represents reduced gene expression. **b**, **c** Examples of integrative analysis predicting how to simultaneously improve a combination of three key wood properties without significant impact on plant growth—downregulating two gene families (*PtrPAL* and *PtrCCoAOMT*) (**b**) or downregulating three gene families (*PtrPAL*, *PtrC3H*, and *PtrCCoAOMT*) (**c**). Each scatterplot is composed of 100 data points, representing 1 to 100% of wildtype transcript abundance

Our integrative analysis (84 integrated equations, Supplementary Data 2) can guide the strategic engineering of wood for better timber and more efficient conversion to pulp and liquid biofuels. High quality timber is dense, strong, and resistant to pests and pathogens, whereas wood pulp and saccharification benefit from reduced wood recalcitrance and increased carbohydrate content. To understand the extent and direction to which specific monolignol genes or combinations of these genes affect lignin and other wood properties, we used the integrative analysis to predict the consequences of varying transcript abundance of a single monolignol gene or a gene family, while all other gene transcripts remained at wildtype levels (Fig. 10, Supplementary Data 10). Different genes and gene families show distinct and specific effects on lignin and wood properties (Fig. 10a, Supplementary Data 10, Supplementary Table 4, Supplementary Note). The effects of changing the expression of an entire gene family are greater than the effects of changing the expression of individual genes within a family (Supplementary Data 10), which indicates that individual family members are functionally redundant and maintain wildtype levels of lignin and wood formation.

Our integrative analysis can be used to identify the best approach for improving single or combinations of multiple lignin and wood properties, while minimizing negative effects on growth. For example, we identified perturbation approaches for a maximal increase in three key properties, i.e., wood density, saccharification efficiency, and the ratio of carbohydrate to lignin (C:L), for more efficient biofuel and pulp/paper production, without significant impact on growth. We analyzed all possible combinations of monolignol gene perturbations, where each gene family was either upregulated, downregulated or remained at wildtype level (Methods). We then ranked these perturbations by the extent of improvement over wildtype for their predicted lignin and wood properties. For a single gene family perturbation, the integrative analysis predicts that the downregulation of *PAL* delivers the best overall improvement in wood quality. Compared to wildtype, *PAL* downregulation by 95% is predicted to exhibit ~70% increase in glucose release and ~260% increase in xylose release during saccharification, and ~60% increase in the C:L ratio (Supplementary Data 10). To achieve maximal improvement in all three key properties simultaneously, perturbation of multiple gene families is needed. Downregulation of both *PAL* and *CCoAOMT* genes by 95% (Fig. 10b) is predicted to increase wood density by ~53% over wildtype, with ~75 and ~220% increase in glucose and xylose release, respectively, and ~60% increase in the C:L ratio (Fig. 10b). Downregulation of *PAL*, *C3H* and *CCoAOMT* gene families is predicted to yield the best overall wood quality (Fig. 10c), with ~41% increase in wood density, ~153 and ~456% increase in glucose and xylose release, respectively, and ~140% increase in the C:L ratio (Fig. 10c).

Our integrative analysis provides a foundation for future work to incorporate additional regulatory processes that affect lignin and wood properties. For example, epigenetic and transcriptional regulation of monolignol gene expression could be incorporated to understand how developmental (e.g., G-lignin and S-lignin cell-types) and genetic variation affect these properties. Novel pathway components, such as the recently discovered caffeoyl shikimate esterases^23–25^, could also be incorporated and may further increase predictive power. To develop the designed transgenic trees with predicted wood and growth properties for utilization, field trials are essential to validate their viability. Field data on key growth and development regulations linking lignin and wood formation with environmental effects (i.e., biotic and abiotic stresses) could then be integrated into our analysis for a more comprehensive strategic engineering of wood properties.

## Discussion

Genetic perturbation of monolignol biosynthetic pathway expands the range of variation in monolignol gene transcripts, lignin and wood traits, and saccharification efficiencies, compared to a natural population of *P. trichocarpa*^26,27^. Sufficient downregulation of any monolignol gene family can modify the properties of lignin and wood. In many cases, these gene-specific downregulations also affect plant development. Specific combinatorial effects of changing the expression of multiple genes are necessary for trait modifications to alleviate negative effects on plant growth and adaptation. Our integrative analysis enables strategic designs for such combinatorial effects. The analysis has also indicated significant novel regulatory components that have not yet been investigated, such as where transcripts and proteins are not well correlated, and epistatic effects imply feed-back regulation. Field testing of the analysis in transgenic *Populus* will reveal more novel regulation associated with specific growth and adaptation effects that can be incorporated into the engineering designs. Our current integrative analysis is more directly applicable to wood formation in *Populus* spp. The applicability in other economically important species, such as Eucalypts and pines, remains to be determined. Monolignol biosynthetic pathway genes and metabolic fluxes may vary broadly across genera. With applicable multi-omics data, the integrative analysis approach used here provides a unique strategy that would allow improvements in multiple lignin and wood properties in any woody species. It also provides a general strategy for rigorous definitions of other biological pathways that could lead to a more comprehensive understanding and description of plant metabolism, growth, and adaptation.

## Methods

### Plant materials

*P. trichocarpa* genotype Nisqually-1 was used for all experiments. Wildtype and transgenic trees were grown in ½ Miracle-Gro Soil (Scotts Miracle-Gro products, Maysville, OH, USA) and ½ Metro-Mix 200 (Sun Gro, Bellevue, WA, USA) in a greenhouse (16 h light/8 h dark cycle with supplementary light of ~300 µE m^−2^ s^−1^)^28^. Wildtype trees for transformation were 6 months old. All transgenic and the corresponding wildtype trees in soil were grown in a greenhouse for 6 months before harvest for characterization.

### Constructs for transgenesis

Gene downregulation constructs were prepared for individual genes, phylogenetic gene-pairs, and gene families. We designed three types of RNAi constructs (types I–III, Supplementary Fig. 1a) and one type of amiRNA construct (type IV, Supplementary Fig. 1a) for these downregulations. Type I RNAi constructs can knock-down individual genes, such as only one xylem-specific family member (e.g., *PtrC3H3* or *PtrCCR2*) (Supplementary Table 1). Type II RNAi constructs with two cDNA silencing fragments (S1 & S2) suppress multiple genes. A type III RNAi construct with four cDNA silencing fragments suppress more genes simultaneously. Type IV is an amiRNA construct used to knock-down individual member within gene families that share high sequence similarities (>85%), such as genes in the *PAL* and *C4H* families.

Using the downregulation of *PtrPAL* genes as an example, five amiRNA constructs (Supplementary Table 1) were used to suppress each *PtrPAL* gene independently. A *P. trichocarpa* miRNA gene, *ptr-MIR408*, was used as the transgene to deliver the 21-nt mature amiRNA silencing sequence complementary only to a specific target gene region for transcript cleavage (knock-down). Then a type II RNAi construct (Supplementary Table 1) with two cDNA silencing fragments (S1 & S2) was designed to suppress the paired *PtrPAL1* and *3* genes. Another type II RNAi constructs (Supplementary Table 1) was designed to suppress the group of *PtrPAL2, 4*, and *5* genes. A type III RNAi construct (Supplementary Table 1) with four cDNA silencing fragments was designed to suppress all five *PtrPAL* genes simultaneously.

All of these constructs contained the *pBI* binary vector backbone. Its original 35S promoter was replaced by the xylem-specific promoter of *Ptr4CL3*, which we named pBI121-4CLXP plasmid^5^. A xylem-specific promoter ensures that the downstream transgene is specifically expressed in the wood-forming tissue of the transgenic trees. To prepare the gene knockdown constructs, the original *GUS* sequence of the pBI121-4CLXP plasmid was replaced by an RNAi or amiRNA transgene.

To assemble the RNAi transgene that targets multiple genes, such as genes in the same subgroup or all members of a gene family, one 150–300 bp sequence sharing over 70% sequence similarity between different target genes but less than 70% with untargeted genes was selected and used as the RNAi sequence. Such an RNAi sequence fragment was amplified from a cloned gene transcript sequence^4^. If that RNAi sequence did not have sufficient similarity to all target genes (>70%), more than one similar sequence was prepared and then assembled by overlapping PCR to form the type II and type III RNAi transgenes.

All RNAi fragments were amplified using primers with 3′ sequences that contained appropriate restriction sites. The amplified fragments were digested with restriction enzymes and assembled with a 600 bp GUS linker sequence (GL) to form an antisense: GL: sense fragment as an RNAi transgene sequence and cloned into an intermediate plasmid^29^. The assembled RNAi transgene fragments, confirmed by sequencing, were subcloned into the pBI121-4CLXP plasmid to replace the original GUS sequence^5^.

The amiRNA transgenes were prepared based on the Ptr-MIR408 transcript. The original miRNA408 and miRNA408* sequences were replaced by designed amiRNA and amiRNA* sequences using overlapping PCR^4^. All amiRNA sequences were designed using the online program WMD (http://wmd.weigelworld.org)^30^ based on the genome of *P. trichocarpa* v1.0 (JGI). The amiRNA sequences were designed using the “no off-target” option, i.e., the amiRNA had only one specific binding site in the target gene and avoided any possible off-target sites in other genes. AmiRNA* sequences and primer sets for integrating amiRNA and amiRNA* sequences into an amiRNA gene backbone were designed using the WMD program’s oligo function under the vector option RS3000 (MIR319a). To integrate the designed amiRNA and amiRNA* sequences into the Ptr-MIR408 transgene backbone, primer sets were modified by replacing the vector sequence of Ath-MIR319a (RS300 vector) with that of Ptr-MIR408. pBI121-based amiRNA expression binary vectors were assembled following Shi et al. (2010)^31^, except that the promoter-specific forward primer was designed for the xylem-specific promoter of the *Ptr4CL3* gene^5^. Transformation construct for simultaneously overexpressing *PtrCAD2* and downregulating *PtrCAD1* (type O, Supplementary Table 1) was prepared by the insertion of full-length *PtrCAD2* coding sequence after the *4CLXP*, and the cassette containing 4CLXP-PtrCAD2-NosT was inserted into the *PtrCAD1* (type I) construct. All binary constructs were introduced into *Agrobacterium tumefaciens* C58 for plant transformation^28^.

### Transgenic plant production

*P. trichocarpa* genotype Nisqually-1 was transformed to downregulate genes for monolignol biosynthesis by *Agrobacterium* transformation^28^ and RNAi or amiRNA constructs (Supplementary Fig. 1a)^30^ listed in Supplementary Table 1. To confirm positive transgene insertions, genomic DNA of putative plantlets was extracted from young leaf tissues using the Qiagen DNeasy Plant Mini Kit (Invitrogen/Life Technologies, Grand Island, NY). PCR assays using promoter forward primers and transgene-specific reverse primers were conducted for the putatively transformed plantlet along with a positive control, the transformation vector plasmid DNA, and a negative control, the genomic DNA from a wildtype tree. For each construct, we produced ~15 independent transgenic lines. Two vegetatively propagated copies of each line were grown in a greenhouse, and 6-month-old trees were analyzed by qRT-PCR to quantify the levels of target gene knock-down. To select transgenic lines that exhibit different levels of knock-down gene expression, stem-differentiating xylem tissues of transgenic lines of 6-months-old greenhouse-grown trees were collected for RNA extraction. Stem-differentiating xylem was scraped from the surface of the debarked stems using single-edge razor blades^32,33^. The collected tissues were frozen and ground into powder in liquid nitrogen. Total RNA was extracted using the Qiagen RNeasy Mini Kit (Invitrogen/Life Technologies, Grand Island, NY). Target gene transcript abundance was analyzed using qRT-PCR as in our previous studies^4^. Three to fourteen independent transgenic lines were selected per construct to represent varying levels of target gene expression. The selected lines were propagated to 9–15 copies each in the greenhouse. In this way, we produced ~2000 transgenic *P. trichocarpa* trees for our integrative analysis. All transgenic trees were grown in the greenhouse along with wildtype controls under the same conditions^29^. The same tissue and wood samples for each transgenic line and wildtype were used for all analyses (RNA, protein, and wood) (sections below).

Six-months-old trees were harvested for characterization. For each line, three biological replicates were collected; each replicate was a pool of 3–5 clonally propagated trees. Fresh stem-differentiating xylem tissues were collected and stored in liquid nitrogen for analysis by RNA-seq and absolute protein quantification. The corresponding wood samples were collected for quantification of MOE, wood density, lignin content and composition, 2D NMR, wood composition and saccharification efficiency. The transgenic trees were produced and harvested in batches at different times each with wildtype controls, which were used to normalize data collected from different batches (Supplementary Note).

### Quantitative estimation of transcript abundance by RNA-seq

Total RNA from each replicate pool (containing 3–5 clonally propagated trees) was extracted using the RNeasy standard protocol (Qiagen, Valencia, CA). Each RNA sample was tested for concentration and purity using a NanoDrop spectrophotometer (Thermo Scientific, Wilmington, DE). The preparation of RNA libraries for sequencing followed Li et al. (2012)^34^, using the TruSeq RNA sample preparation kit (Illumina, San Diego, CA). The RNA libraries were pooled for multiplex sequencing using the Illumina GAIIx platform (Genomic Sciences Laboratory, North Carolina State University, Raleigh, NC). We mapped the FASTQ files from the Illumina GAIIx onto the genome of *P. trichocarpa* v3.0^3,35–37^, using Bowtie^38^ and Tophat^39^. BAM (Binary Sequence Alignment/Map) files were converted to raw counts using BedTool^40^, and normalized read counts were obtained using the Trimmed Means of *M* values^35–37,41^.

### Protein quantification by PC-IDMS

Total protein extracts were isolated from stem-differentiating xylem and processed using filter-aided sample preparation^9^. The absolute protein abundance of the monolignol biosynthetic enzymes was measured by PC-IDMS using a C18 column (75 µm × 15 cm), reversed phase nanoLC and selected reaction monitoring on a TSQ triple quadrupole mass spectrometer (Thermo Scientific, San Jose, CA)^9^. The spectral data was processed and integrated using Skyline^42^ to determine the absolute quantities of proteins in stem-differentiating xylem.

### Wood chemistry analysis of transgenics and wildtype

After stem-differentiating xylem tissue collection for RNA and protein isolation, the remaining wood stem segments (devoid of internodes 1–5) from the same transgenic and wildtype trees were used for wood chemistry analysis. The stem segments were extracted with 90% acetone for 48 h, followed by three additional extractions (each 48 h) using 100% acetone, and air-dried. The extractive-free stem segments were ground to a fine powder using a Wiley mill and sieved to 40–60 mesh and vacuum dried over P_2_O_5_. Acid-insoluble lignin and acid-soluble lignin contents were determined following the Klason procedure^43^. Sugars in the acid-soluble lignin fractions were quantified by a gas chromatography-flame ionization detector (GC-FID 7890A; Agilent, Santa Clara, CA)^43^, or neutralized using CaCO_3_, filtered through a 0.2 µm PVDF membrane (Pall Corporation, Port Washington, NY), and analyzed by an Infinity 1200 HPLC (Agilent, Santa Clara, CA). Pure compounds of glucose, galactose, xylose, mannose, and arabinose (Sigma, St. Louis, MO) were used as standards. The sum of lignin and sugar contents averages 91.6% (% = g/100 g dry wood) for wildtype trees; the uronic acid and acetyl contents in wood likely account for much of the remainder.

### Quantification of lignin composition and interunit linkages

To ensure that we analyzed essentially the entire lignin fraction of the wood, we utilized nothing more than enzymatic saccharification and did not attempt to further purify the lignin, utilizing the power of the whole-cell-wall 2D NMR methods^44,45^ to resolve components and extract data. To enrich lignin from the wood samples of transgenics and wildtype for 2D NMR analysis, extractive-free wood samples were ground to 40–60 mesh and vacuum dried under P_2_O_5_. The wood samples were then milled using a Pulverisette 7 Planetary Micro Mill (Fritsch, Idar-Oberstein, Germany) at 600 rpm, with six cycles of 30 min on and 15 min off. The milled samples were incubated with cellulase (Sigma C9422, St. Louis, MO) in the ratio of 1 g wood per 450 units of cellulase, for 48 h at 48 °C. The samples were washed twice with an acetate buffer (pH 4.5, 20 mM), followed by two more washes using distilled water, and were freeze dried. The lignin samples were analyzed without further purification to retain the entire lignin without fractionation. The lignin samples were dissolved in DMSO-d_6_/pyridine-d_5_ (4:1, v/v), then analyzed by a 700 MHz Bruker NMR instrument fitted with a cryoprobe for improved sensitivity^44^. S, G, H, and *p*-hydroxybenzoic acid levels were determined by integrating the H2/C2 correlations using Bruker’s Topspin 3.5 software (and expressing on an S + G + H = 100% basis); relative interunit linkage levels were from integrating Hα/Cα correlations and are expressed on the basis of the sum of the (β–*O*–4) + (β–5) + (β–β) + (β–1) levels^45^.

### Quantification of wood modulus of elasticity

Three 20-cm long stem sections were cut from the base of each transgenic and wildtype sample. The stem sections were kept in sealed plastic bags to prevent drying. The stem sections were cut to a length-to-width ratio of 16:1 and analyzed using a three-point bending test^46^ by a universal mechanical tester (MTS Insight, Eden Prairie, MN) to measure the MOE.

### Quantification of wood density

One-inch thick disks were cut from the base of each stem and were used to measure wood density (specific gravity) of each tree using the water displacement method^47^. Samples were weighed, dried in an oven at 103 ± 2 °C overnight and the oven dry mass measured. The density was calculated following the ASTM standards (D2395)^47^.

### Wood pretreatment and enzymatic hydrolysis

Extractive-free wood samples from wood chemistry analysis were either (1) subjected to hot-water pretreatment at 180 °C for 5 min in 96-well reactor plates (pretreated, see below), or (2) without pretreatment (unpretreated) prior to enzymatic hydrolysis. Enzymatic hydrolysis of unpretreated and pretreated samples was carried out using a high-throughput pretreatment and incomplete-saccharification hydrolysis technique to allow comparisons^48^. Briefly, 5 ± 0.3 mg dry extractive-free wood was weighed into custom-made 96-well Hastelloy reactors with Mobile Tool Management robotics (FreeSlate, Sunnyvale, CA). The reactor plates were sealed and pretreated (5 min at 180 °C) after 250 µL of water was added to each well. After cooling, 40 mL of CTec2 cellulase (Novozymes, Franklinton, NC) diluted in 1.0 M citrate buffer pH 5.0 was added to each well to a final loading of 70 mg protein/g glucan. For unpretreated samples, CTec2 was added directly to the reactor plates at the same concentrations as the pretreated samples. The reactor plates were sealed, and the enzymatic hydrolysis was performed at 50 °C for 70 h. The released glucose and xylose in the supernatants were quantified by sugar-specific oxidation-reduction assays^48^. For each independent wood sample, three analytical replicates were performed. Sugar release data were reported as weight percentage (wt%) of cell wall residues^48^ and then converted to % of total glucose or xylose content.

### Normalizing transcript and protein abundances and regression

We collected stem-differentiating xylem tissues in six separate batches at different times. Each batch of transgenic trees was planted with wildtype controls. We normalized the RNA-seq read counts of specific transcript and the quantities of specific peptides against the average of three replicates of wildtype in each batch (Supplementary Fig. 3). Linear regression for the transcript (predictor) and protein (dependent variable) abundances of the monolignol biosynthetic pathway genes in the stem-differentiating xylem of wildtype and transgenic *P. trichocarpa* was performed using JMP software (JMP^®^, Version 12, SAS Institute Inc., Cary, NC, 1989–2007).

### Integrative analysis

The integrative analysis is represented by equations 1 to 84 (Supplementary Data 2), coded in MATLAB (Supplementary Data 9). There are three main components to the integrative analysis (1–3, Fig. 2b): (1) the transcript/protein equations (2) the mass-balance kinetic equations and (3) the multiple linear regression equations used to predict the phenotypes (Fig. 2c). The integrative analysis is based on wood formation that consists of a mix of different cell types (fiber, vessel, and ray cells).

#### Transcript/protein equations

The integrative analysis takes the absolute transcript abundances as the input to predict protein abundances using simple linear regressions (*β*_i_ values, Supplementary Table 2). The simple linear regressions were constrained to intercept 0 (i.e., zero transcript = zero protein). The absolute transcript abundances in µM were estimated from the RNA-seq libraries^49^. Briefly, the raw read counts were normalized to reads per kilobase per million mapped reads, and calibrated to µM using monolignol transcript abundances determined from qRT-PCR of wildtype *P. trichocarpa* stem-differentiating xylem^49^. The predicted protein abundances were used as the input for the mass-balance kinetic equations.

#### Mass-balance kinetic equations

The mass-balance equations by Wang et al. (2014)^5^ assumed independence between the monolignol enzyme functions^5^. However, we showed that the two Ptr4CL isoforms (Ptr4CL3 and Ptr4CL5, Fig. 1), each with distinct kinetic and inhibitory properties, form a heterotetrameric protein complex in vivo in the ratio of 3:1^12^. This 4CL protein complex affects the specificity and reaction rates of CoA ligation (fluxes 7 to 11, Fig. 1)^12^, improves the stability/robustness of the pathway^50^, and constitutes an important component of metabolic regulation^12^. Therefore, we modified the mass-balance equations from Wang et al. (2014)^5^ to include the updated flux equations from Chen et al. (2014)^12^ and Lin et al. (2015)^7^, which incorporate the heterotetrameric 4CL protein complex for fluxes 7 and 8 (Fig. 1), and the associated complex enzyme inhibition kinetics. The modified equations (equations 21 to 59, Supplementary Data 2) were coded into MATLAB 2015b using the ordinary differential equation solver (ODE15s) to predict pathway metabolic-fluxes and metabolite concentrations. We presented evidence that three monolignol cytochrome P450 monooxygenases (PtrC4H1, PtrC4H2, and PtrC3H3) interact to form heteromeric protein complexes^51^. However, mass-balance kinetic equations that describe these interactions were not part of the integrative analysis because they are difficult to determine, and biochemical techniques are not currently available to assay the membrane-bound enzymes at different molar ratios. To determine the initial conditions, we used MATLAB and equations 21 to 59 to solve for the phenylalanine concentration when wildtype S/G ratio is 2.16 (equivalent to fluxes V35/V34, Fig. 1), a quantity based on our 2D NMR analysis of isolated lignin from the stem-differentiating xylem of wildtype *P. trichocarpa* (Supplementary Data 4). MATLAB shows that a phenylalanine concentration of 1.4 µM is necessary to produce a wildtype S/G ratio of ~2.16. The remaining metabolite concentrations vary dynamically in MATLAB until a steady-state is reached. The simulation time was set from 0 to 10,000 s, which provides sufficient time for the metabolic-fluxes and metabolite concentrations to reach steady-state values. We tested 10,000 sets of random initial metabolite concentrations (Latin Hypercube Sampling) to determine all possible steady-states and found 92% of all simulations settled at the same steady-state, suggesting that the pathway is robust to changing initial conditions. 4-Coumaryl alcohol (20, H-subunit precursor, Fig. 1), coniferyl alcohol (22, G-subunit precursor), and sinapyl alcohol (24, S-subunit precursor) are formulated as precursors of the terminal products (H, G, and S-subunits) that accumulate over the course of the flux analysis.

#### Multiple linear regressions

Using JMP Pro 12 (SAS Institute Inc., Cary, NC), an all-possible-model approach was used to determine which of the metabolites and metabolic-fluxes are the best linear predictors for each of the lignin and wood properties. The all-possible-model approach tests all combinations of metabolites and fluxes as predictors to identify the best multiple linear regressions (equations 60 to 84, Supplementary Data 2) that exhibit global minima in the corrected Akaike’s information criterion (AICc). To reduce multicollinearity, metabolites and fluxes with correlation coefficients >0.95 were omitted from the multiple linear regressions. The predicted lignin and wood properties from the multiple linear regressions are restricted to non-negative values, and the properties that have the unit of percentage (e.g., β–*O*–4 linkages) are restricted to a maximum value of 100%.

### Integrative analysis validation

The integrative analysis was validated using 5-fold cross-validation. For the 5-fold cross-validation, each of the 239 transgenic and wildtype lines was randomly sorted into five approximately equal sized groups. Using JMP Pro 12 (SAS Institute Inc., Cary, NC), an all-possible-model approach was used to determine which of the metabolites and fluxes were the best linear predictors for each group, based on the lowest AICc value. The all-possible-model algorithm re-estimates all regression parameters including the transcript/protein *β*_i_ values and the multiple linear regression constants. To prevent the algorithm from choosing fluxes and metabolites with very small values and assigning them a very large scaling factor, the metabolite concentrations and fluxes were rounded to five decimal places for this process. The predictors that yield the lowest AICc value were chosen to be included in the integrative analysis (Supplementary Data 2).

Direct validation of metabolite concentrations and metabolic-fluxes derived from the mass-balance equations (equations 21–59, Supplementary Data 2) is not possible because there is no reliable extraction technique that permits the quantitation isolation of monolignol pathway metabolites. We have investigated extensively the quantification of monolignol pathway metabolites in vivo, by synthesizing stable-isotope-labeled standard compounds and using advanced LC-MS/MS systems. Our analysis confirmed that monolignol pathway metabolites could not be reliably extracted from wood forming tissues for quantification. Similarly, metabolic-fluxes for monolignol biosynthesis cannot be validated experimentally, due to the complex enzyme inhibitory network that regulates the pathway. These technical difficulties have long prohibited the use of metabolite concentrations and metabolic-fluxes for experimental validation. The most direct means of validating the integrative analysis is by association to the content, composition, and linkage structures of lignin, which we applied in our five-fold cross-validation. Cross-validation is suitable here because its re-sampling technique allows for efficient use of all available data and provides a robust estimation of the validity of predictions with minimal bias and variability. Considering the integrative analysis is based on ~2000 transgenic trees, a small independent experiment would be insufficient to provide an adequate validation.

### Genetic engineering strategies for optimal wood properties

To identify the optimal approaches for improving any single or combination of lignin and wood properties, we used the integrative analysis to investigate all possible combinations of monolignol gene perturbations. Each gene family transcript abundance was either (1) upregulated to 1000% of the wildtype level, (2) retained at the wildtype level, or (3) downregulated to 5% of the wildtype level. Gene upregulation by 1000% is an estimate of the level of gene-specific overexpression feasible by transgenesis, and represents an extrapolated projection of how increasing monolignol gene expression affects lignin and wood traits. Such extrapolation is outside of the experimental data and should be interpreted with caution. In silico gene perturbations were limited to a simultaneous modification of one, two, or three gene families. Untargeted gene families were set at the wildtype level because indirect effects were not included in the integrative analysis equations (Supplementary Data 2). A total of 1161 unique combinations of monolignol gene family perturbations were tested using the integrative analysis (equations 1 to 84, Supplementary Data 2). The predicted lignin and wood properties were then individually ranked from the highest value to the lowest, or vice versa, depending on the desired traits. The gene perturbations that produced the most desired traits were reported in Fig. 10a. For maximal improvement of multiple lignin and wood properties, the best perturbation approaches were identified by ranking each predicted outcome based on the extent of the overall improvement over wildtype for the specific desired properties. To do this, we applied proportional scoring^52^ to each predicted outcome using the equation:

$$\mathop {\sum }\limits_{i = 1}^N ({\mathrm {predicted}}\;{\mathrm {property}}_i - {\mathrm {desired}}\;{\mathrm {property}}_i)^2$$

Where *N* represents the number of desired properties. The scores were then ranked from the lowest value (most desired outcome) to the highest (least desired outcome) to identify the best perturbation approaches (Fig. 10b, c).

### Data availability

The RNA-seq libraries are available under GEO accession number GSE78953. Proteomics, MOE, wood density, 2D NMR, plant growth, and wood chemistry data sets are available on CyVerse [http://mirrors.iplantcollaborative.org/browse/iplant/home/shared/LigninSystemsDB]. Correspondence and requests for materials should be addressed to V.L.C. (vchiang@ncsu.edu).

## Electronic supplementary material

Supplementary Information

Description of Additional Supplementary Files

Supplementary Data 1

Supplementary Data 2

Supplementary Data 3

Supplementary Data 4

Supplementary Data 5

Supplementary Data 6

Supplementary Data 7

Supplementary Data 8

Supplementary Data 9

Supplementary Data 10
