# Supplementary material for: Improving wood properties for wood utilization through multi-omics integration in lignin biosynthesis
Source: Nat Commun. 2018 Apr 20;9:1579. doi: 10.1038/s41467-018-03863-z (PMC5910405; doi:10.1038/s41467-018-03863-z)
Supplement: Supplementary file 1 — Supplementary Information [file 41467_2018_3863_MOESM1_ESM.docx]

**Improving wood properties for wood utilization through multi-omics integration in lignin biosynthesis**

Wang *et al.*

**Supplementary Note 1**

**Monolignol biosynthesis:** Monolignols (4-coumaryl, coniferyl, and sinapyl alcohols that are the precursors to the H-, G- and S-subunits in lignin) are biosynthesized from phenylalanine through a metabolic grid consisting of 10 enzyme families and 24 metabolites (**Fig. 1**)^1,2^. In stem-differentiating xylem, a wood-forming tissue of *Populus trichocarpa*, five isoforms of phenylalanine ammonia-lyase (PtrPAL1–5) catalyze the deamination of phenylalanine to cinnamic acid^1,3-5^, which is then hydroxylated sequentially to 4-coumaric acid and caffeic acid by a membrane protein complex consisting of two isoforms of cinnamic acid 4-hydroxylases (PtrC4H1 and PtrC4H2) and a 4-coumaric acid 3-hydroxylase (PtrC3H3)^6^. 4-Coumaric acid is also activated to 4-coumaroyl-CoA by a hetero-tetrameric protein complex of 4-coumaric acid: CoA ligases (Ptr4CL3 and Ptr4CL5) in a 3:1 ratio^7^. 4-Coumaroyl-CoA can be reduced to 4-coumaryl alcohol (H-subunit) by a cinnamoyl-CoA reductase (PtrCCR2) and cinnamyl alcohol dehydrogenases (PtrCAD1 and PtrCAD2)^1^, or hydroxylated to caffeoyl-CoA by the cooperative reactions of two isoforms of hydroxycinnamoyl-CoA shikimate hydroxycinnamoyl transferases (PtrHCT1 and PtrHCT6)^4,8^, and the PtrC4H1/PtrC4H2/PtrC3H3 protein complex^6^. Recently, a caffeoyl shikimate esterase (CSE)^9-11^ was discovered in Arabidopsis that hydrolyzes caffeoyl shikimic acid to caffeic acid. Caffeoyl-CoA is methylated to feruloyl-CoA by three isoforms of caffeoyl-CoA *O*-methyltransferases (PtrCCoAOMT1–3), which is then reduced to coniferyl alcohol by PtrCCR2, PtrCAD1, and PtrCAD2^1,4^. Two isoforms of coniferaldehyde 5-hydroxylases (PtrCAld5H1 and PtrCAld5H2) and a 5-hydroxyconiferaldehyde *O*-methyltransferase (PtrAldOMT2) convert coniferaldehyde to sinapaldehyde, and coniferyl alcohol (G-subunit) to sinapyl alcohol (S-subunit) (**Fig. 1**)^12-14^.

**Generation of 221 transgenic lines of *P. trichocarpa* with modified expression of the core monolignol biosynthetic genes:** Transgenic perturbation provides a wide range of levels of gene expression and the downstream responses. Such variation is the foundation for integrative analysis because it allows us to determine the linearity of the relationships and to calculate the regression parameters, such as translation efficiency (*β*_i_ values, **Supplementary Table 2**). Genes expressed at a fixed transcript level may exhibit weak correlations with their corresponding protein abundance^15^. In contrast, genes expressed at a range of different levels may correlate well with their corresponding protein abundance^16^. Using a systematic gene perturbation approach with transgenic *P. trichocarpa*, we modified the expression of the 21 core genes involved in monolignol biosynthesis (**Fig. 1**, **Supplementary Table 1**) used in this study. Of the 21 pathway genes, 10 (from 5 families) had been targeted by transgenesis in previous studies (*PtrPAL*^17^, *Ptr4CL*^18^, *PtrC3H*^19^, *PtrCCR*^20^, and *PtrCAD*^20^), and 11 are perturbed for the first time in transgenic *P. trichocarpa*. The pathway for monolignol biosynthesis has been a target for genetic manipulation across diverse plant species^21^. We produced 28 transgene constructs for *P. trichocarpa* transformation (**Supplementary Table 1**, **Methods**). These include three types of RNA interference constructs (RNAi) (types I–III, **Fig. 1**), and one type of artificial microRNA construct (amiRNA) (type IV, **Fig. 1**). Type I RNAi constructs targeted the knock-down of individual genes, where gene-specific silencing is relatively predictable; such as for monolignol gene families with only one xylem-specific member (**Fig. 1**, **Supplementary Table 1**). Type II RNAi constructs contained two cDNA silencing fragments (S1 and S2) and were used to suppress monolignol phylogenetic gene-pairs (**Fig. 1**, **Supplementary Table 1**). A type III RNAi construct containing four cDNA silencing fragments was used to suppress all five members of the *PtrPAL* family (*PtrPAL1*–*5*) (**Fig. 1**, **Supplementary Table 1**). The amiRNA constructs were used to target single gene-specific knock-downs within families (**Fig. 1**, **Supplementary Table 1**)^22^. *PtrCAD2* transcript abundance is very low in stem-differentiating xylem^4^. Therefore, we used *PtrCAD2* overexpression (type O, **Supplementary Table 1**) to obtain broad variation in the levels of *PtrCAD2* transcript for the integrative analysis (**Methods**). The transgene’s expressions were driven by a Ptr4CLXP promoter for stem-differentiating xylem-specific expression^13^. Using these 28 transgene constructs, we produced ~2,000 transgenic trees. For each transgene construct, 3 to 14 independent lines (for a total of 221 lines) (**Supplementary Table 1**) exhibiting varying levels of transgene expression were selected and clonally propagated to 3 to 5 copies per replicate pool for characterization (**Methods**). All 221 transgenic lines and 18 wildtype samples were characterized by RNA-seq and quantitative proteomics (**Methods**). Our analysis of wood chemistry, lignin 2D NMR, MOE, density, and saccharification efficiency covered an extensive range of transgenic events (**Supplementary Data 3–8**). However, due to the large quantity of wood required for these specific analyses, not all the samples had enough wood or tissues for characterization by every analysis (**Fig. 2A**).

**Transcriptomic analysis of transgenic and wildtype *P. trichocarpa* stem-differentiating xylem:** Integrative analysis of transcriptomes depends on accurate quantification of gene expression^23^. The whole transcriptome RNA-seq is suitable for such quantification at the genome level with high sensitivity and reproducibility^24^. RNA-seq also allows deep sequencing of a specific tissue to determine which potential isoforms are expressed and functionally implicated in a pathway^25^. Using RNA-seq, we profiled the stem-differentiating xylem transcriptomes of all 221 transgenic lines and 18 wildtype controls of *P. trichocarpa*. About 5.06 billion sequence reads (0.53 terabases) were generated from the 239 RNA-seq libraries, averaging 21.2 ± 0.1 million reads (2.2 ± 0.1 billion bases) per library (**Supplementary Fig. 1c**). The RNA-seq reads showed on average an 89.9 ± 0.6% alignment rate to the *P. trichocarpa* genome v3.0. The biological coefficient of variation (BCV) that describes variation in transcript abundance across the 239 RNA-seq libraries showed a common dispersion of 0.217 (**Supplementary Fig. 1d**), which is lower than the BCV typically observed in transcriptomes of genetically identical organisms (BCV of ~0.25)^26^. This result indicates that our transcriptomes exhibit minimal global ectopic effects^26^. Reads mapping to *PtrPAL4* and *PtrPAL5* were quantified as a sum of the two genes because the surrogate peptide used for their corresponding protein quantification was a shared sequence, preventing independent quantification by protein cleavage-isotope dilution mass spectrometry (PC-IDMS)^27^. All transgene constructs were able to significantly knock-down their targeted monolignol biosynthetic genes in the transgenic *P. trichocarpa* (**Fig. 3a**, **b**, **Supplementary Data 1**).

**Normalization of transcript and protein abundances for regression analysis:** Our transgenics and wildtype were produced over time and data measurements were taken for the batches, which introduced batch-to-batch variations^28^. Batch effects were observed for most monolignol genes in the transgenic and wildtype *P. trichocarpa* (**Supplementary Fig. 3**). Batch effects arise predominantly from variations due to seasonal effects. Normalization is an effective and commonly used approach to correct batch effects^29^. We normalized the transcript and protein abundances using wildtype samples for each batch (**Methods**). Normalization corrected the batch effects (**Supplementary Fig. 3**) and resulted in an approximate normality of the transcript and protein abundances across the six batches, and therefore enabling the integration of data to examine the relationships between transcripts and proteins.

**The relationships between the abundances of transcripts and proteins:** The transcription/ translation processes are central components of gene regulation in the control of development and metabolism and therefore essential to understanding any specific metabolic pathway such as lignin biosynthesis. The efficiency of translation of transcript to protein is a complex process involving the formation of an initiation complex, protein factors, and tRNA-dependent elongation, and is also dependent on transcript abundance, sequence, structure, and stability^23,30^. To our knowledge, there is no previous work in plants on the efficiency of translation of all the genes involved in a specific metabolic pathway. Such information would provide insights into the coordinated regulation of the metabolic pathway, and how the regulation is controlled. Transcript abundance can predict protein abundance in yeast, bacteria, insects, nematodes, and human cells^31-35^. Most studies show weak to moderate correlations between transcript and protein abundance^15^.

For the proteins of the monolignol pathway, we could estimate the relative abundance of transcript equivalents measured as RNA-seq fragment reads, relative to the abundance of specific peptide fragments measured by quantitation of surrogate tryptic peptides in the wood-forming tissue of *P. trichocarpa.* Transcript abundance was a significant predictor of protein for genes *PtrPAL2* (coefficient of determination, R^2^=0.53), *PtrPAL4|5* (R^2^=0.48), *PtrCAld5H1* (R^2^=0.41), and *PtrC4H1* (R^2^=0.41). In bacteria and eukaryotes, such relationships are typically ~0.4 (R^2^)^23^. A strong transcript/protein relationship indicates that transcript level is a major determinant of the corresponding protein abundance. In genes *PtrCCoAOMT3*, *PtrHCT1*, *Ptr4CL5,* and *PtrHCT6*, transcript abundance was a poor predictor of protein quantity as shown by low R^2^ values (0.03 to 0.15) (**Supplementary Table 2**). Such weak transcript/protein relationships suggest that regulatory processes after transcription exert significant control over their protein abundance. These processes may include post-transcriptional, translational, and protein degradation regulation^23^. Some monolignol proteins are post-translationally modified by protein phosphorylation and glycosylation^36,37^.

The general relationship between transcript abundance and protein abundance (**Fig. 4**, **Supplementary Fig. 2**, **Supplementary Table 2**) for genes in the monolignol pathway is linear with a positive slope of 14,980 ± 5150 molecules of proteins per molecule of transcript. The protein-per-transcript ratios are consistent with the observation that stability (half-life) of proteins is usually a matter of days, compared to the transcripts where the half-life is more a matter of hours^15^. Therefore, the abundance of protein is likely to accumulate relative to the abundance of the transcript. However, different genes have dramatically different translational efficiency, which ranges from 2610 to 104,730 molecules of protein per transcript (*β*_i_ values, **Supplementary Table 2**). Therefore, even for genes in the same metabolic pathway, the conversion of their transcripts to proteins may be regulated differently.

Different translational efficiency was also observed between members of the same monolignol gene family. For example, the regression slopes (*β*_i_), or the number of protein molecules produced per molecule of the transcript, are much higher for the xylem-specific *PAL* genes (*PtrPAL2* and *4|5*) compared to the non-xylem-specific *PAL* genes (*PtrPAL1* and *3*) (**Supplementary Table 2**). The two *HCT* genes also show different regression slopes. For each molecule of the transcript, *PtrHCT6* produces ~480% more proteins than *PtrHCT1*. Similarly, *Ptr4CL3* transcripts produce ~200% more proteins than do the *Ptr4CL5* transcripts. Ptr4CL3 and Ptr4CL5 form a heterotetrameric protein complex in a ratio of 3:1^7^. Perturbations that alter the ratios of Ptr4CL3 to Ptr4CL5 could therefore affect metabolic-flux for monolignol biosynthesis. The *CCoAOMT* family is encoded by three members, *PtrCCoAOMT1*, *PtrCCoAOMT2* and *PtrCCoAOMT3*. The three *CCoAOMT*s have similar and high protein/transcript ratios of 12,720, 13,440 and 10,820, respectively. The cytochrome P450 monooxygenases *PtrC4H1, PtrC4H2* and *PtrC3H3* also have similar ratios of protein/transcript abundance. PtrC4H1, PtrC4H2, and PtrC3H3 form a heteromeric membrane protein complex^6^. The similar protein/transcript ratios suggest co-regulation of translational efficiencies of the protein complex.

The inclusion of transcript to protein relationships in an integrative analysis provides greater utility than an integrative analysis based on proteins alone. Traditional tree breeding and modern genetic engineering technologies such as RNAi, amiRNA, and CRISPR all rely on quantifying changes in transcript abundance. Transcripts can be reliably quantified by many genomic techniques, whereas absolute protein quantification remains challenging. We generated simple linear regression equations representing the efficiency of translation of transcripts to proteins for the monolignol biosynthetic pathway genes (equations 1–20, **Supplementary Data 2**). The regression equations were incorporated into the integrative analysis of monolignol biosynthesis (**Supplementary Data 9**) to predict the absolute abundance of the pathway proteins in the stem-differentiating xylem of *P. trichocarpa*, based on the corresponding transcript abundances.

To investigate the extent to which uncertainties between monolignol transcript and protein abundances impact predictive power of the integrative analysis, we tested either transcript or protein abundances as input to predict the 25 lignin and wood properties (**Fig. 2c**) in the transgenics and wildtype. An all-model-approach (**Methods**) was used to determine the multiple linear regression equations when protein abundances were the input. Similar average adjusted R^2^ values were observed for the 25 lignin and wood properties when either transcript (adjusted R^2^ = 0.70 ± 0.15) or protein (adjusted R^2^ = 0.64 ± 0.16) abundances were the input, suggesting that uncertainties between monolignol transcript and protein abundances do not significantly impact predictive power.

**Lignin composition and interunit linkages of transgenic and wildtype *P. trichocarpa*:** Perturbation of monolignol gene expression may cause significant changes in the biosynthesis of the type and quantity of monolignols, and result in altered lignin composition and the relative levels of the various interunit linkages. To quantify the effects of gene perturbation on lignin properties, we first developed and optimized a method to isolate the maximum possible yield of lignin with intact structural features (with their characteristic interunit linkages) (**Methods**). Using this optimized method, we performed 2D NMR analysis of 76 wildtype and transgenic *P. trichocarpa* lignin samples (**Supplementary Data 4**). Wildtype lignin is composed on average of 67.8% S-subunits, 31.9% G-subunits, 0.3% H-subunits, and 3.5% *p*-hydroxybenzoic acid (on an S + G + H = 100% basis); with 6.3% cinnamyl alcohol end-groups (end-groups are usually over-estimated in HSQC spectra^38^) (**Supplementary Data 4**). The subunits are linked on average by 86.5% β–*O*–4 linkages, 3.7% β–5 linkages, 7.5% β–β linkages, and 2.3% β–1 linkages (**Supplementary Data 4**).

In transgenic lines that target the downregulation of *PtrC3H3* (NSF3-i20-5, **Supplementary Data 4**), we observed a drastic increase in H-subunits (to 44.8%) (**Fig. 7b**), significantly reduced G-subunits (to 5.1%), and mildly reduced S-subunits (to 50.2%). This change in lignin composition is consistent with *C3H* downregulated hybrid poplar (*P. alba* × *P. grandidentata*)^39^. *p*-Hydroxybenzoic acid was increased (to 6.3%) (**Fig. 7**b, **Supplementary Data 4**). The increase in H-subunits may be the result of reduced metabolic-fluxes that convert 4-coumaric and 4-coumaroyl shikimic acids to caffeic and caffeoyl shikimic acids, respectively (fluxes 3 and 13, **Fig. 1**). Consequently, metabolic-flux may be redirected to the biosynthesis of 4-coumaryl alcohol for incorporation as H-subunits in lignin (20, **Fig. 1**). The remaining flux may then be preferentially directed to the biosynthesis of S-subunits (24, **Fig. 1**)^12,13^, resulting in a significant increase in the lignin S/G ratio (9.93) (**Fig. 7b**), despite the decreased S-subunit level. β–Aryl ether (β–*O*–4) unit levels in *PtrC3H3* transgenics were slightly lower (**Supplementary Fig. 5b**, **Supplementary Data 4**), consistent with the decreased S-subunit levels. Monolignols have a higher probability of β–aryl ether coupling to S-subunits in the growing lignin polymer during lignification because of the extra methoxyl group^39^. Spirodienones (β–1) were also reduced. Phenylcoumarans (β–5) and resinols (β–β) were higher (to ~230% and ~170% of wildtype, respectively) (**Supplementary Fig. 5b**, **Supplementary Data 4**) in the *PtrC3H3* transgenics. The main reason for these observations is likely the increased H-subunits.

Transgenic lines that target the downregulation of *PtrC4H1* (NSF4-a10, **Supplementary Data 4**) or *PtrC4H2* (NSF4-a9) showed no significant changes in lignin composition. The lignin aliphatic area of the HSQC spectra also did not show significant changes. However, for transgenic lines that target the downregulation of three monolignol hydroxylases (*PtrC3H3*, *PtrC4H1,* and *PtrC4H2*) (NSF3-i69-4, **Supplementary Data 4**), H-subunit levels were significantly elevated to 32.8% (**Fig. 7e**) compared to the wildtypes (0.3%, **Supplementary Data 4**). S- and G-subunits were both reduced (to 49.4% and 17.8%, respectively) (**Fig. 7e**) compared to the wildtypes. Spirodienones (β–1) were reduced in the transgenics (**Supplementary Fig. 5e**, **Supplementary Data 4**). There were new peaks at 7.79 ppm/128.8 ppm and 7.74 ppm/131.2 ppm, which represent structures that we are not yet able to identify (**Fig. 7e**).

For the *PtrCCR2* downregulated transgenic lines, the ferulic acid in lignin, although a minor component, was clearly increased (from ~0% to ~0.9%) compared to the wildtypes (**Fig. 7f**). A ferulic acid marker (*bis*–β–*O*–4–ether) that has previously been detected in *CCR*-downregulated plants^40^ was also identified in the *PtrCCR2* downregulated *P. trichocarpa* lignin (**Supplementary Fig. 6a–d**). *PtrCCR2* downregulation results in a minor reduction of S-subunits and a minor increase in G-subunits, reducing the S/G ratio to 1.43 (**Fig. 7f**) compared to the wildtypes (2.44) (**Fig. 7a**). A reduction in resinol units (β–β) to 3.3% (**Supplementary Fig. 5f**) was observed compared to the wildtypes. The reduction in S/G ratio and the reduction in resinol units are consistent because most resinol structures in lignin come from sinapyl alcohol dimerization^39^.

All *PtrCAD1* and *PtrCAD2* downregulated transgenic lines showed significantly elevated levels of aldehydes in lignin, compared to the wildtype (**Fig. 7g**, **Supplementary Data 4**). Aldehyde levels reached 30.4% in the most severely downregulated *PtrCAD* line (NSF3-i35-7, **Supplementary Data 4**), compared to ~4% in the wildtypes (**Supplementary Data 4**). Products resulting from the endwise coupling of coniferaldehyde and sinapaldehyde into the lignin polymer were readily observed in the 2D HSQC NMR spectra (**Fig. 7g**), confirming that the 4-hydroxycinnamaldehydes are functioning as lignin subunits^40^. *PtrCAD* downregulation did not affect the ratios of H:G:S in lignin (**Fig. 7g**, **Supplementary Data 4**). However, the *p*-hydroxybenzoic acid level (14.8%) (NSF3-i35-7, **Supplementary Data 4**) was drastically elevated compared to the wildtype (3.5%), suggesting the activation of a *p*-hydroxybenzoic acid specific pathway by *PtrCAD* downregulation. The transgenic lignin shows a minor increase in β-aryl ether (β–*O*–4) linkages, and a corresponding reduction in resinol (β–β) and phenylcoumaran (β–5) contents (NSF3-i33 and i35, **Supplementary Fig. 5g**, **Supplementary Data 4**). The reduced resinol and phenylcoumaran contents are likely due to the reduced S- and G-subunits, respectively^39^.

For transgenic lines that target the downregulation of *PtrHCT1* (NSF5-a17-4-1, **Supplementary Data 4**) or *PtrHCT6* (NSF5-a18-9-1), we observed minor increase in H-subunits (to 3.6% and 1.4%, respectively), compared to the wildtypes (0.3%). As a result, resinol (β–β) contents were slightly reduced, and phenylcoumaran (β–5) contents were slightly increased (**Supplementary Fig. 5h**, **i**, **Supplementary Data 4**). In contrast, when both *PtrHCT1* and *PtrHCT6* were downregulated (NSF5-i19-7-1, **Fig. 7j**, **Supplementary Data 4**), we observed a significant increase in H-subunits (to 29.2%), S-subunits were significantly reduced (to 47.1%), and G-subunits were slightly reduced (23.7%) (NSF5-i19-7-1, **Fig. 7j**, **Supplementary Data 4**). The drastic difference in lignin composition between single *PtrHCT* downregulation and the downregulation of both *PtrHCT*s suggests functional redundancy of *PtrHCT1* and *PtrHCT6*. Despite the large increase in H-subunits and reduction of S-subunits, lignin interunit linkage distributions were not significantly affected by the downregulation of *PtrHCT*s (**Supplementary Fig. 5j**, **Supplementary Data 4**), a feature that has been noted previously^41^.

Transgenic lines that target the downregulation of *PtrCAld5H1* (NSF5-a27-2-1, **Supplementary Data 4**) or *PtrCAld5H2* (NSF5-a28-3-1) showed significant reduction in S-subunits (to 39.0% and 41.4%, respectively) (**Fig. 7k**, **l**), and correspondingly increased G-subunits (to 60.4% and 58.4%, respectively) (**Fig. 7k**, **l**). Consequently, S/G ratio was reduced from 2.44 in wildtype (**Fig. 7a**) to 0.65 and 0.71, respectively for *PtrCAld5H1* and *PtrCAld5H2* transgenic lignin (**Fig. 7k**, **l**). One *PtrCAld5H2* downregulated line (NSF5-a28-1-1, **Supplementary Data 4**) showed significantly elevated H-subunit content (to 11%), which suggests additional regulation of the pathway. In transgenic lines that downregulated both *PtrCAld5H1* and *PtrCAld5H2* (NSF5-a29-2-1, **Supplementary Data 4**), S-subunits in lignin were further reduced to 11% and G-subunits were increased to 88.8% (**Fig. 7m**), resulting in a S/G ratio of 0.12 (**Fig. 7m**). *p*-Hydroxybenzoic acid content was reduced from 2.8% in the wildtypes to 1.4% in transgenic lignin (**Fig. 7m**, **Supplementary Data 4**). The drastic alteration in lignin composition also affected lignin interunit linkage distributions. Phenylcoumaran (β–5) contents were increased to 16.3% (**Supplementary Fig. 5m**) due to the increase in G-subunits. These results confirmed that the activities of PtrCAld5H1 and PtrCAld5H2 are additive for the biosynthesis of sinapyl alcohol (24, **Fig. 1**), and that the abundance of total PtrCAld5H is the primary determinant of S/G ratio in lignin ^12^. Downregulation of *PtrPAL* genes and *PtrCCoAOMT* genes resulted in no significant change in lignin composition or interunit linkages in *P. trichocarpa*, compared to the wildtypes (**Supplementary Data 4**).

**Modulus of elasticity:** Modulus of elasticity (MOE) represents the extent of wood deformation when a constant force is applied. MOE is an important index for the evaluation of wood mechanical properties and is crucial for solid wood and engineered wood applications^42^. MOE was measured perpendicular to the longitudinal axis for stem segments of 416 trees (**Supplementary Data 6**). The 416 trees correspond to 36 wildtypes and 128 replicate pools, with each pool containing 2 to 5 clonally propagated trees (**Methods**).

**Wood density:** Wood density (as specific gravity) is the ratio of the density of wood to the density of water. Most mechanical properties and physical properties are closely related to wood density^42^. Little is known about the density of wood of transgenic trees with altered monolignol genes. Horvath et al. (2010)^43^ found reduced wood density in transgenic quaking aspen (*P. tremuloides* Michx.) with reduced lignin content and increased S/G ratio.

We measured the wood density for 213 transgenic and wildtype trees (**Supplementary Data 7**). The wood density of the transgenic lines showed a large range from 0.26 to 0.43 with an average of 0.32 (**Supplementary Data 7**). The wildtypes have an average wood density of 0.36 (**Supplementary Data 7**), which is similar to the average wood density of mature *P. trichocarpa* trees reported^42^. The lowest wood densities were found in *PtrCAD*, *PtrC3H,* and *PtrC4H* transgenics produced in our study (**Supplementary Data 7**).

**Integrative systems analysis:** The integrative analysis captures the variation in the 25 key lignin and wood properties of our 239 transgenics and wildtype. Using transcript abundance as the only input, the integrative analysis explained a high percentage of the phenotypic variation in lignin content, composition, and C:L ratio (R^2^ from 0.71 to 0.91, **Fig. 9a–h**). Major lignin interunit linkages are also accurately explained (R^2^ from 0.62 to 0.95, **Fig. 9i–m**). The integrative analysis captures plant height, diameter, and stem volume with R^2^ values of 0.63, 0.55, and 0.56, respectively (**Fig. 9n–p**). Glucose, xylose, and total carbohydrate contents are captured with R^2^ values of 0.40, 0.60, and 0.44, respectively (**Fig. 9q–s**). Wood density and MOE are explained with R^2^ values of 0.65 and 0.71 (**Fig. 9t**, u). The integrative analysis of saccharification efficiency of unpretreated wood captured 73% and 77% of the variation in glucose and xylose release (**Fig. 9v**, **w**), and captured 62% and 68% of the variation in glucose and xylose release from pretreated wood (**Fig. 9x**, **y**).

Our integrative analysis can guide gene perturbation strategies to improve specific lignin and wood traits (**Fig. 10a**). For example, it predicts that lignin content can be reduced by severe downregulation of any monolignol gene family except for *CCoAOMT*, *CAD,* and *CAld5H* (**Fig. 10a**, **Supplementary Data 10**). The strongest lignin reduction is predicted for the downregulation of *C3H*, *CCR*, or *HCT* families (**Fig. 10a**), but these perturbations also inhibit growth (**Supplementary Data 10**). In contrast, the integrative analysis shows that downregulating the *PAL* or *C4H* genes can reduce lignin to ~8.7% (a 60% reduction based on wildtype lignin content) without significant effects on growth (**Supplementary Data 10**). The integrative analysis predictions are generally consistent with our experimental transgenic results, except for the *CCoAOMT* transgenics (**Supplementary Data 3**). A discrepancy between predictions and experimental observations may lead to novel mechanistic insights into lignin biosynthesis and the downstream effects of changing gene expression. The integrative analysis shows that the reduced lignin in *CCoAOMT* transgenics (**Supplementary Data 3**) is the consequence of epistatic effects that reduced the abundance of 4CL, HCT, CAD and AldOMT proteins (**Fig. 3b**), and not a direct result of reduced CCoAOMT activity.

**Monte Carlo simulations:** Monte Carlo simulation is a mathematical technique that uses repeated random sampling of predictor variables to derive a probability distribution for all possible outcomes ^44^. We used Monte Carlo simulation to explore the combinatorial output space of the integrative analysis that is beyond the space of the transgenics, thereby estimating the range and distribution of all possible lignin and wood properties attainable by changing monolignol gene expression. Transcript abundances with ±25%, ±50%, and ±100% variation from the wildtype values were input to the integrative analysis (equations 1–84, **Supplementary Data 2**) and the resulting lignin and wood properties were calculated. The Monte Carlo simulations show that increasing transcript variation in monolignol gene expression is much more likely to reduce lignin as opposed to increase lignin (**Supplementary Fig. 4a**). In contrast, saccharification efficiency is more likely to be increased in response to increasing variation in transcript abundance (**Supplementary Fig. 4k**). The Monte Carlo simulations also show that a subset of the randomly sampled expression levels of the monolignol genes may produce wood with improved phenotypic traits, such as elevation of S/G ratio, wood density, mechanical strength, and saccharification efficiency (**Supplementary Fig. 4**).

The Monte Carlo simulations (**Supplementary Fig. 4**) show that a ±25% variation around wildtype monolignol transcript abundance results in phenotypic distributions that are similar to the variations observed in wildtypes, indicating that stronger gene perturbations (>25%) are needed to affect lignin and wood properties. The predicted lignin and wood properties for ±50% or ±100% variation of the wildtype transcript abundances (**Supplementary Fig. 4**) show significant differences in their distributions when compared to the wildtype. Despite the increased transcript variation, the phenotypic distributions remained approximately normal, with the mean value similar to the wildtype, indicating a preferred steady-state wildtype level of gene expression effects (**Supplementary Fig. 4**). This result suggests a homeostatic response of the major lignin and wood properties to changes in gene expression.

**Global sensitivity analysis:** To determine the extent to which variation in lignin and wood properties can be apportioned to specific monolignol gene expression, we performed a global sensitivity analysis using Latin Hypercube Sampling (LHS)^45^ and Partial Rank Correlation Coefficient (PRCC) (**Supplementary Table 4**). LHS was used to randomly sample 2,000 sets of monolignol transcript abundances within the range of expression levels observed in the transgenics and wildtype (e.g., 4–307% for *PtrPAL1*, GEO accession number: GSE78953). PRCC of the 2,000 monolignol transcript profiles and their corresponding lignin and wood properties (predicted by the integrative analysis) then estimate the influence of each monolignol gene transcript on lignin and wood. A PRCC of 1 indicates that the gene transcript has a strong positive influence on the property; a PRCC of -1 indicates a strong negative influence; and a PRCC of 0 indicates no influence.

All monolignol gene families significantly influence (PRCC>0.3 or <-0.3, *p*<0.05)^46^ one or more lignin and wood properties (**Supplementary Table 4**). The extent and direction of influence varies among each gene family. For example, wood density is negatively influenced by *PtrCCoAOMT*s (PRCC = -0.685 to -0.101), but positively influenced by *PtrCCR2* (PRCC = 0.726). Within a gene family, members can influence the same properties (such as *PtrCAld5H*s), which suggests functional redundancy^12^. In contrast, gene family members that are distinct in their enzyme activities (such as Ptr4CL3 and Ptr4CL5)^7,47^ tend to influence different lignin and wood properties (**Supplementary Table 4**).

Of all the 500 possible combinations of a single monolignol gene influencing a single lignin and wood property, only 61 (12.2%) are significant (PRCC>0.3 or <-0.3, *p*<0.05)^46^. This indicates that each lignin and wood property may be insensitive up to 87.8% of all variations in monolignol gene expression. The insensitivity to gene transcript variation is supported by our transgenic data (**Fig. 5**) and Monte Carlo simulations (**Supplementary Fig. 4**), where the majority of lignin and wood properties approximate the wildtype level, despite modifications to the monolignol gene expression. This result provides further evidence for a homeostatic regulation of lignin biosynthesis in wood formation.

**Metabolic-flux mechanisms are essential for accurate prediction of lignin and wood properties:** To illustrate that the wood phenotypic traits are not simply direct reflections of transcript variation, we compared the predictive capabilities of multiple linear regression equations using either **(1)** metabolic-fluxes and metabolite concentrations as predictors (black bars, **Fig. 8b**) or **(2)** absolute transcript abundances as predictors (red bars, **Fig. 8b**). For the comparison, an all-possible model approach was used to test the specific predictor variables, and the regressions exhibiting lowest AICc values were selected to predict the lignin and wood properties (**Methods**). For 84% (21 out of 25) of the key lignin and wood properties, the predictive capabilities of the integrative analysis are significantly better when using the metabolic-fluxes and metabolite concentrations as predictor variables (black bars, **Fig. 8b**). For 4 (out of 25) wood properties (G-subunits, end-groups, glucose, and total carbohydrates), similar adjusted R^2^ values were observed when either transcript abundances or metabolites and fluxes were used as predictors, indicating that for these 4 properties, the variation is sufficiently captured by monolignol transcript abundances. More accurate predictabilities for metabolite and flux predictors are seen for lignin content, S/G ratio, *p*-hydroxybenzoic acid, C:L ratio, aldehydes, lignin linkage structures, growth, relative density, MOE, and saccharification efficiency (**Fig. 8b**). This result indicates that the transcript abundances (without the metabolic-flux mechanisms) are not enough to sufficiently describe the variations in most wood phenotypic traits characterized, and that the mechanistic-based mass-balance equations are necessary to more accurately predict these lignin and major wood properties.

**
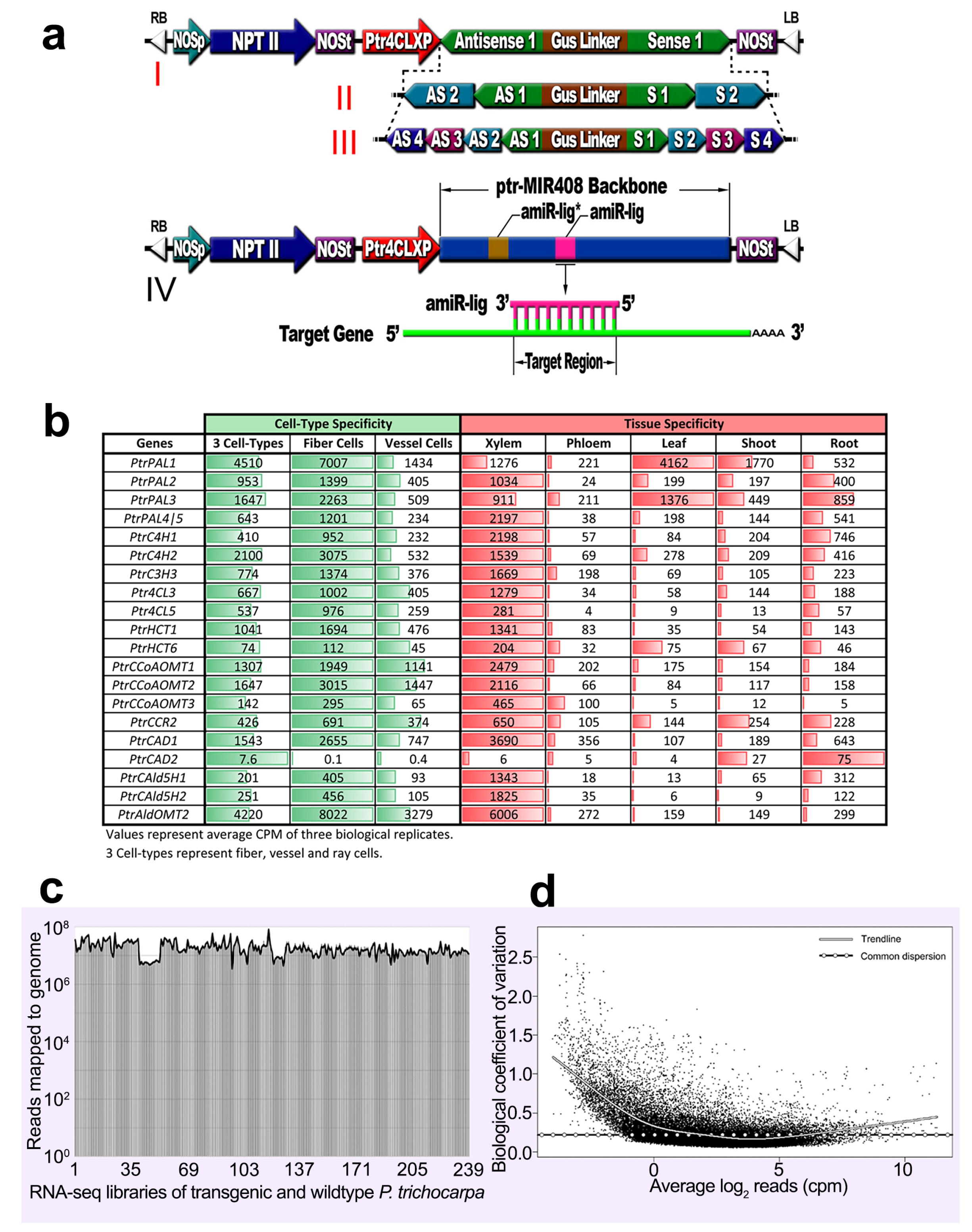
**

**Supplementary Figure 1 │ Transgene constructs, tissue and cell-type expression specificity of monolignol genes, and RNA-seq libraries of the transgenic and wildtype *P. trichocarpa*. a,** RNA interference constructs (types I to III; RNAi) and an artificial microRNA construct (IV; amiRNA) were used for gene-specific downregulation of the monolignol biosynthetic genes. See **Methods** for more details on the preparation of these constructs. Abbreviations: Nopaline synthase promoter (NOSp); neomycin phosphotransferase II (NPT II); nopaline synthase terminator (NOSt); *Ptr4CL3* native xylem-specific promoter (Ptr4CLXP)^13^; sense strand (S); antisense strand (AS); specific 21-nt mature amiRNA silencing sequence (amiR-lig). **b,** The tissue and cell-type expression specificity of the core monolignol biosynthetic genes in *P. trichocarpa*. Green bars show the transcript abundances of the monolignol genes in fiber cells, vessel cells, or a mixture of fiber, vessel, and ray cells (3 cell-types). Monolignol gene expression is generally higher in fiber cells compared to vessel cells. Red bars show the transcript abundances in xylem, phloem, leaves, shoots, and root tissues of wildtype *P. trichocarpa*. The values represent average count-per-million (CPM) reads of three biological replicates. The tissue and cell-type gene expression analysis is described in Chen et al. (2014)^7^. **c,** The transcriptomes of the stem-differentiating xylem of transgenic and wildtype *P. trichocarpa.* The number of sequencing reads that mapped to the *P. trichocarpa* genome v3.0 for each of the 239 RNA-seq libraries (GEO accession number: GSE78953). **d,** The biological coefficient of variation that describes the variation in transcript abundances between the RNA-seq libraries. Each dot represents the average expression and variance for each gene in the genome, with the trend line and the common dispersion overlaid.

**
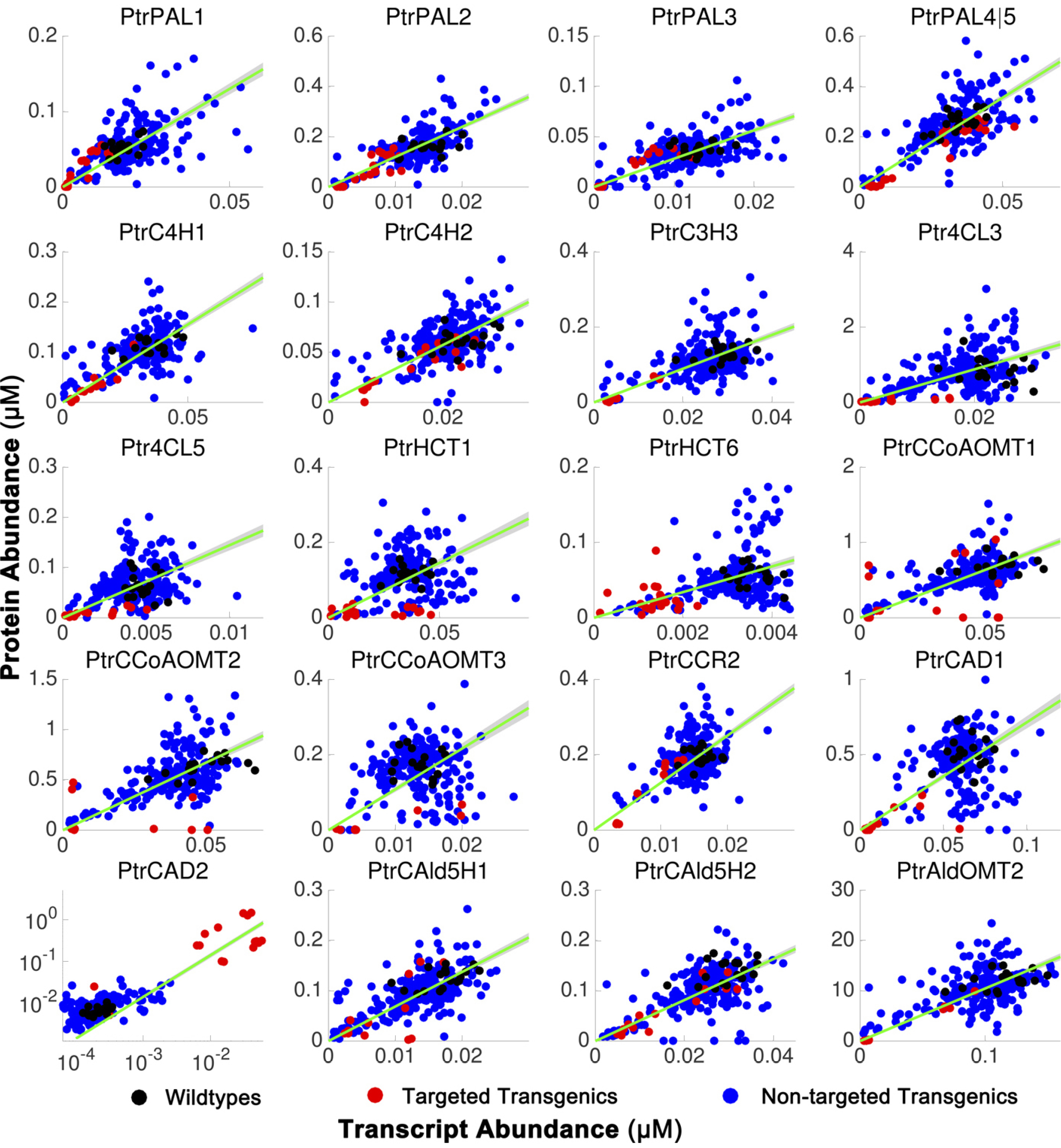
**

**Supplementary Figure 2 │ Relationships between transcript abundance and protein abundance for the monolignol biosynthetic genes in stem-differentiating xylem of wildtype and transgenic *P. trichocarpa* lines.** Black dots represent transcript and protein abundances in 18 wildtypes. Red dots represent transcript and protein abundances of the monolignol genes in transgenics that targeted their downregulation (targeted transgenics). Blue dots represent transcript and protein abundances in transgenics that did not target their downregulation (non-targeted transgenics). Green lines are linear regression fits, with gray shadings representing the 95% confidence intervals. Each dot represents the quantitative measurements of transcripts and proteins for a replicate pool of one wildtype or transgenic line containing 3 to 5 clonally propagated trees.

**
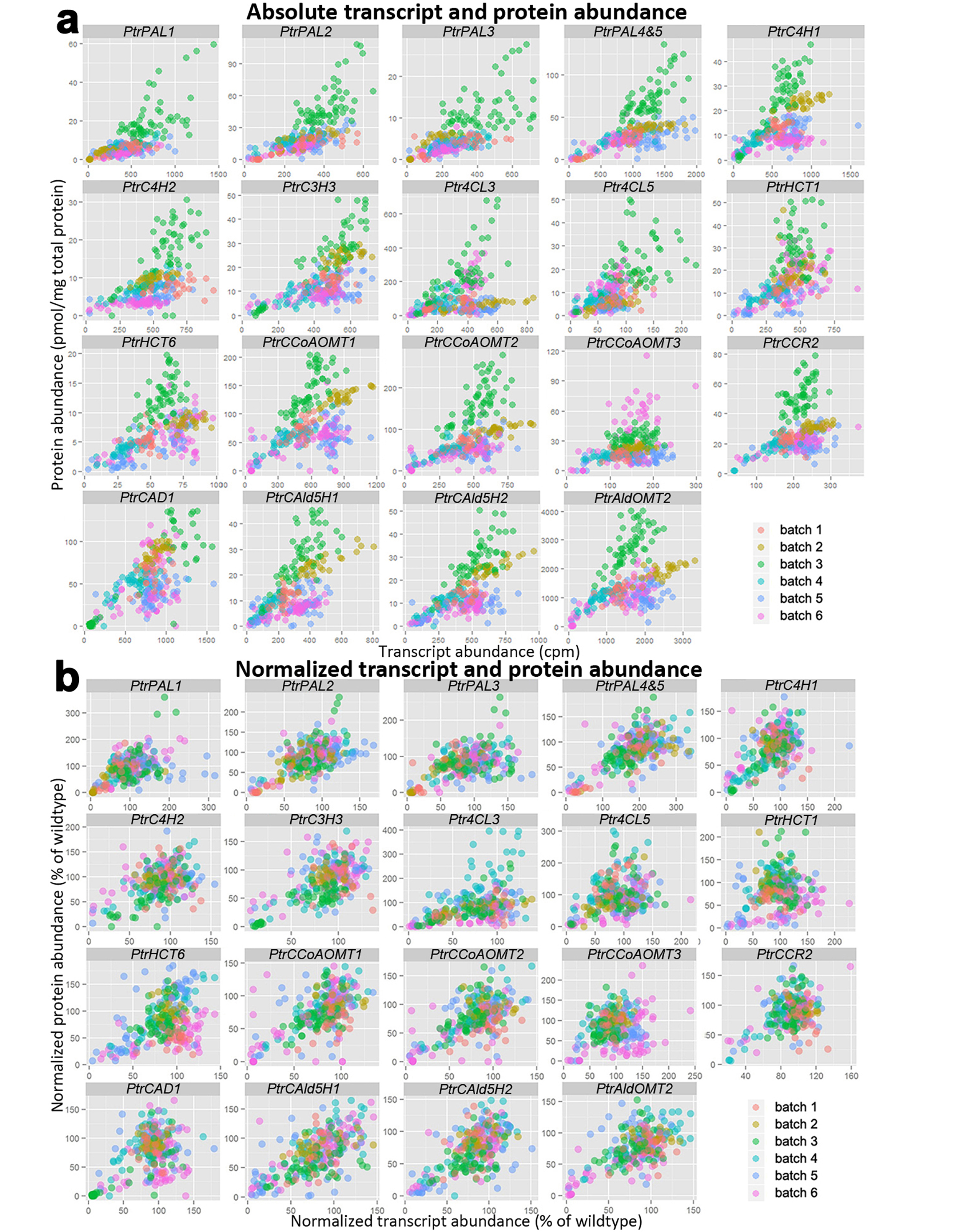
**

**Supplementary Figure 3 │ Normalization of transcript and protein abundances to correct for batch effects.** **a,** Scatterplots of the absolute transcript and protein abundances in wildtype and transgenic trees. **b,** Scatterplots of the normalized transcript and protein abundances in wildtype and transgenic trees. Each dot represents a replicate pool of one wildtype or transgenic line containing 3 to 5 clonally propagated trees. The dots are color coded by their experimental batch numbers (batches 1–6).

**
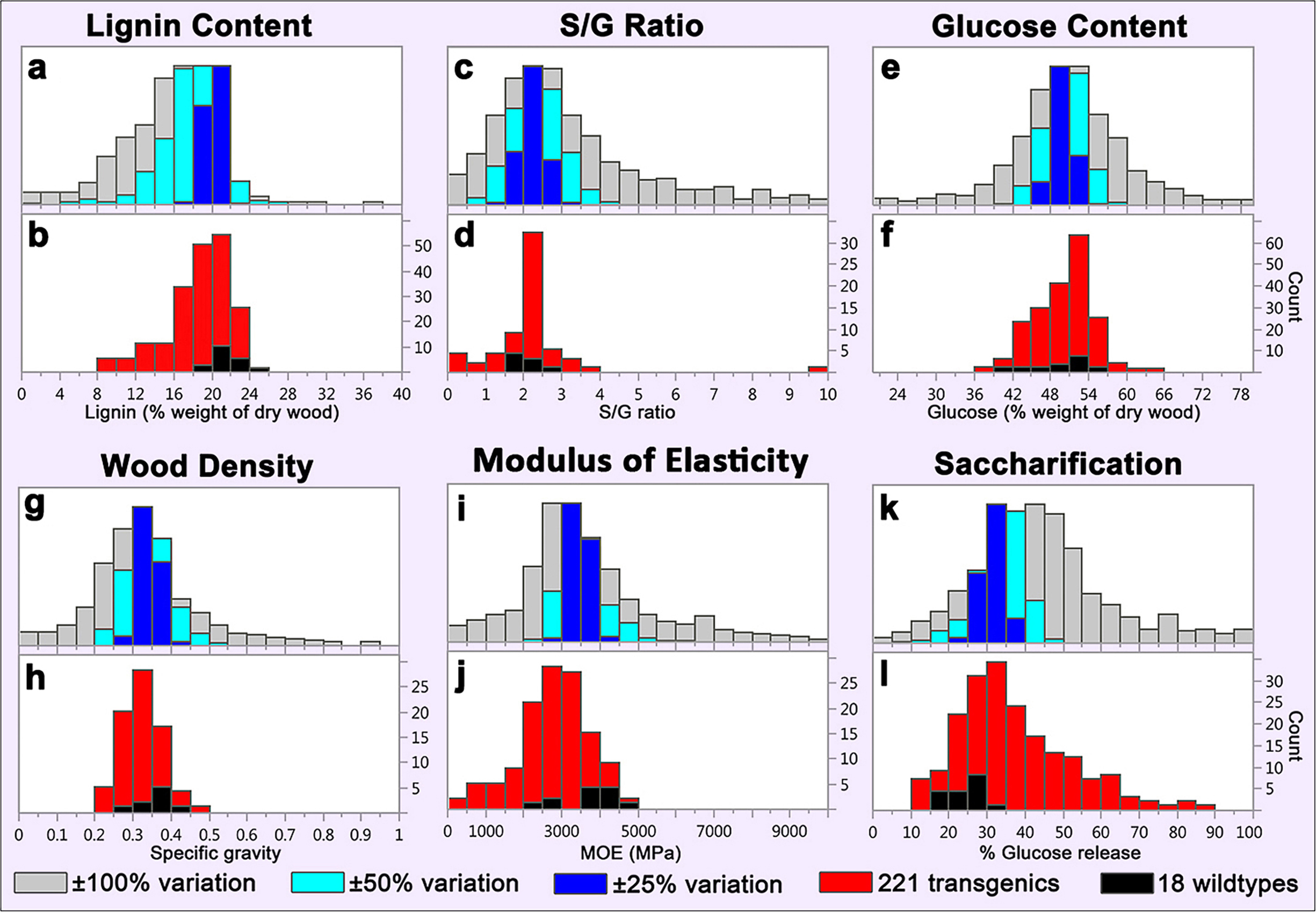
**

**Supplementary Figure 4 │ Overlaid distribution (histograms) of lignin content, S/G ratio, glucose content, wood density, modulus of elasticity, and saccharification efficiency.** Phenotypic distributions (**a**, **c**, **e**, **g**, **i** and **k**) predicted by Monte Carlo simulations with transcript abundances randomly sampled at wildtype level ±25% (blue), ±50% (turquoise), and ±100% (gray) variations (1,000 iterations per level). (**b**, **d**, **f**, **h**, **j** and **l**) Red bars represent distributions of the quantities of lignin and wood properties and saccharification efficiency for the 221 transgenic lines, and black bars represent those for the 18 wildtype samples.

**
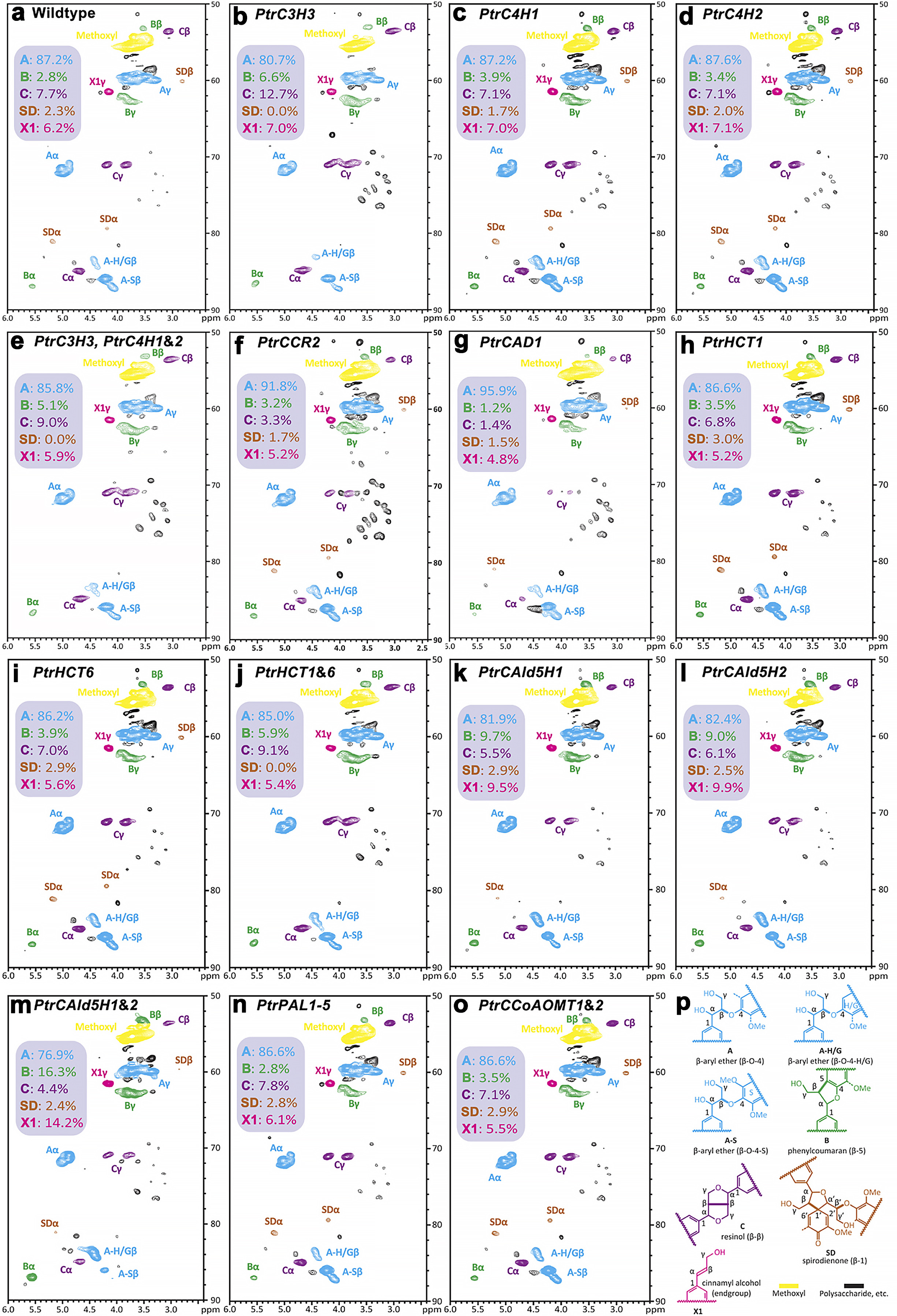
**

**Supplementary Figure 5 │ The interunit linkages of lignin measured by 2D NMR.** Partial (aliphatic region) short-range ^1^H–^13^C correlation (HSQC) spectra of enzyme lignin from wildtype **a,** and an example of transgenic *P. trichocarpa* line for each construct downregulated in the gene expression of **b,** *PtrC3H3*, **c,** *PtrC4H1*, **d,** *PtrC4H2*, **e,** *PtrC3H3* and *PtrC4H1*&*2*, **f,** *PtrCCR2*, **g,** *PtrCAD1*, **h,** *PtrHCT1*, **i,** *PtrHCT6*, **j,** *PtrHCT1&6*, **k,** *PtrCAld5H1*, **l,** *PtrCAld5H2*, **m,** *PtrCAld5H1&2*, **n,** *PtrPAL1*–*5*, **o,** *PtrCCoAOMT1*&*2*. Spectral contours are color-coded according to **p,** the major linkage structures. Percentage volume integrals of major lignin linkages are provided for each spectra. Abbreviations: **A**: β-aryl ether (β–*O*–4); **B**: phenylcoumaran (β–5); **C**: resinol (β–β); **SD**: spirodienone (β–1); **X1**: cinnamyl alcohol end-groups. See **Supplementary Data 4** for all 2D NMR data.

**
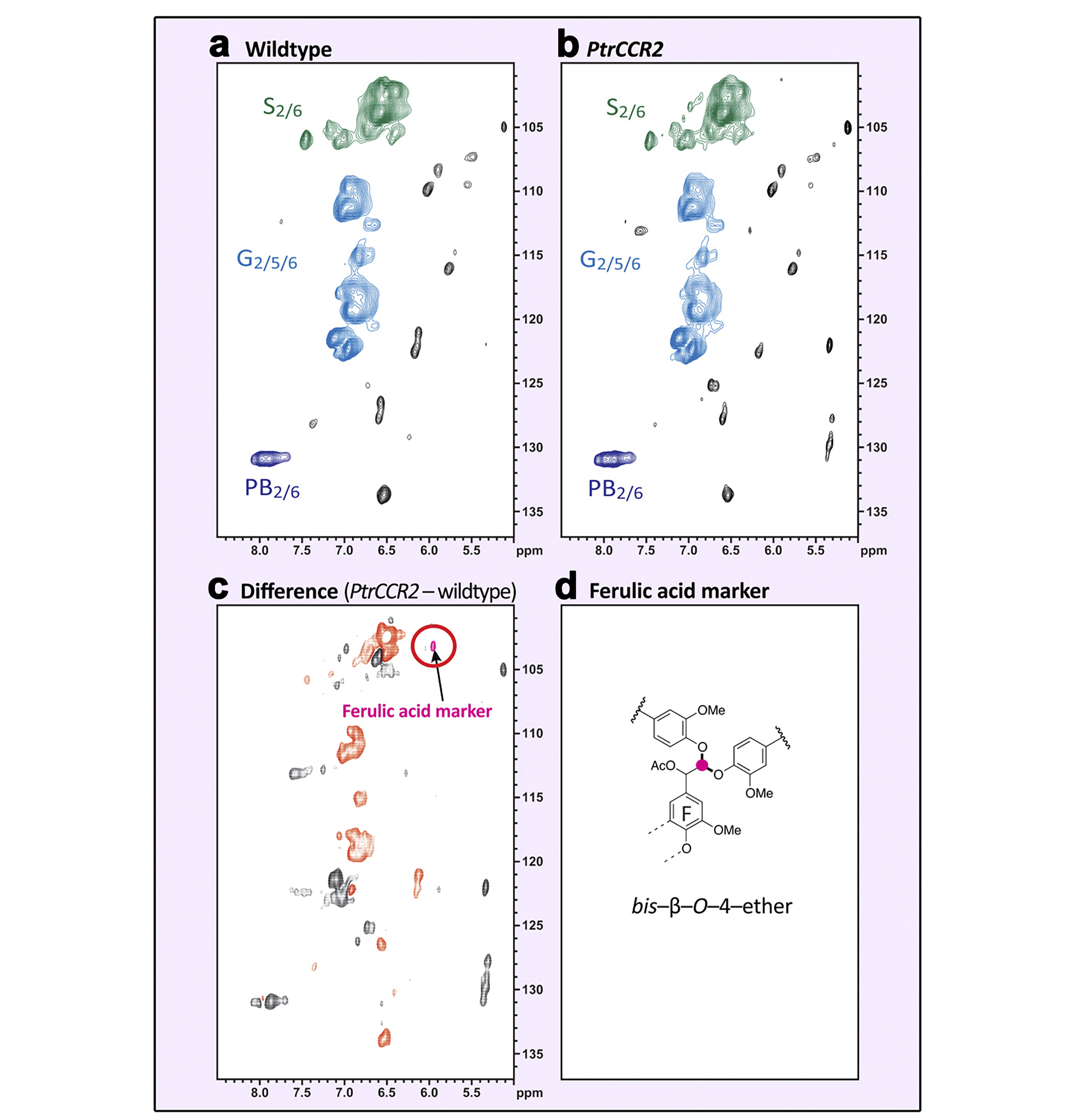
**

**Supplementary Figure 6 │ Ferulic acid marker (*bis*–β–*O*–4–ether) in lignin of *PtrCCR2* downregulated transgenic *P. trichocarpa*.** Partial HSQC NMR spectra for enzyme lignin of **a,** wildtype and **b,** *PtrCCR2* downregulated transgenics. **c,** Difference spectra shows the ferulic acid marker (arrow and magenta contours) resulting from the plant’s use of ferulic acid as a (low-level) monomer. The red contours indicate a higher level of G-subunits in the transgenic sample compared to the wildtype. **d,** The chemical structure of the ferulic acid marker^40^.

**Supplementary Table 1 │** Transformation constructs and the number of *P. trichocarpa* transgenic lines.

| **Gene families** | **Construct types** | **Target gene(s)** | **Number of transgenic lines** |
| --- | --- | --- | --- |
| *PtrPAL* | IV | *PtrPAL1* | 3 |
|  | IV | *PtrPAL2* | 3 |
|  | IV | *PtrPAL4* | 3 |
|  | IV | *PtrPAL5* | 3 |
|  | II | *PtrPAL1* | 9 |
|  |  | *PtrPAL3* |  |
|  | II | *PtrPAL2* | 12 |
|  |  | *PtrPAL4* |  |
|  |  | *PtrPAL5* |  |
|  | III | *PtrPAL1* | 9 |
|  |  | *PtrPAL2* |  |
|  |  | *PtrPAL3* |  |
|  |  | *PtrPAL4* |  |
|  |  | *PtrPAL5* |  |
| *PtrC4H* | IV | *PtrC4H2* | 9 |
|  | IV | *PtrC4H1* | 8 |
|  | II | *PtrC4H1* | 7 |
|  |  | *PtrC4H2* |  |
| *PtrC3H* |  | *PtrC3H3* |  |
|  | I | *PtrC3H3* | 8 |
| *Ptr4CL* | IV | *Ptr4CL3* | 8 |
|  | IV | *Ptr4CL5* | 8 |
|  | I | *Ptr4CL3* | 6 |
|  |  | *Ptr4CL5* |  |
| *PtrHCT* | IV | *PtrHCT1* | 9 |
|  | IV | *PtrHCT6* | 11 |
|  | I | *PtrHCT1* | 11 |
|  |  | *PtrHCT6* |  |
| *PtrCCoAOMT* | IV | *PtrCCoAOMT1* | 6 |
|  | IV | *PtrCCoAOMT3* | 8 |
|  | I | *PtrCCoAOMT1* | 12 |
|  |  | *PtrCCoAOMT2* |  |
| *PtrCCR* | I | *PtrCCR2* | 9 |
| *PtrCAD* | I | *PtrCAD1* | 9 |
|  | II | *PtrCAD1* | 9 |
|  |  | *PtrCAD2* |  |
|  | O | *PtrCAD1* | 14 |
|  |  | *PtrCAD2* (OE) |  |
| *PtrCAld5H* | IV | *PtrCAld5H1* | 3 |
|  | IV | *PtrCAld5H2* | 6 |
|  | II | *PtrCAld5H1* | 9 |
|  |  | *PtrCAld5H2* |  |
| *PtrAldOMT* | I | *PtrAldOMT2* | 9 |
|  |  | **Total** | **221** |

Construct types are described in **Methods** and in **Supplementary Figure 1a**. Types I, II, and III are RNA interference constructs containing 1, 2, and 4 monolignol gene fragments, respectively (**Supplementary Figure 1a**). Type IV is an artificial microRNA construct targeting individual genes within gene families (**Supplementary Figure 1a**). Type O construct downregulates *PtrCAD1* (by RNAi) and overexpresses (OE) *PtrCAD2* (**Supplementary Note**). Three to fourteen transgenic lines exhibiting varying levels of transgene expression were selected for each construct.

**Supplementary Table 2 │** Simple linear regression parameters for the relationship between transcript abundance and protein abundance of the *P. trichocarpa* monolignol biosynthetic genes.

| **Genes** | ***p*-values of the F tests** | **R^2^** | ***β*_i_** (10^3^) ± **SE** | **RMSE** |
| --- | --- | --- | --- | --- |
| *PtrPAL1* | < 0.001 | 0.35 | 2.61 ± 0.07 | 24.1 |
| *PtrPAL2* | < 0.001 | 0.53 | 11.94 ± 0.23 | 48.8 |
| *PtrPAL3* | < 0.001 | 0.37 | 2.82 ± 0.07 | 13.3 |
| *PtrPAL4\|5* | < 0.001 | 0.48 | 7.12 ± 0.14 | 73.7 |
| *PtrC4H1* | < 0.001 | 0.41 | 3.10 ± 0.07 | 33.1 |
| *PtrC4H2* | < 0.001 | 0.32 | 2.85 ± 0.06 | 18.1 |
| *PtrC3H3* | < 0.001 | 0.31 | 4.47 ± 0.11 | 44.4 |
| *Ptr4CL3* | < 0.001 | 0.27 | 43.89 ± 1.57 | 449.0 |
| *Ptr4CL5* | < 0.001 | 0.14 | 14.47 ± 0.52 | 36.6 |
| *PtrHCT1* | < 0.001 | 0.07 | 2.92 ± 0.11 | 61.3 |
| *PtrHCT6* | < 0.001 | 0.15 | 17.04 ± 0.62 | 30.4 |
| *PtrCCoAOMT1* | < 0.001 | 0.32 | 12.72 ± 0.29 | 208.4 |
| *PtrCCoAOMT2* | < 0.001 | 0.33 | 13.44 ± 0.33 | 205.9 |
| *PtrCCoAOMT3* | < 0.001 | 0.03 | 10.82 ± 0.35 | 76.1 |
| *PtrCCR2* | < 0.001 | 0.20 | 12.56 ± 0.22 | 50.4 |
| *PtrCAD1* | < 0.001 | 0.34 | 7.18 ± 0.20 | 182.2 |
| *PtrCAD2* | < 0.001 | 0.45 | 13.94 ± 0.95 | 122.4 |
| *PtrCAld5H1* | < 0.001 | 0.41 | 6.87 ± 0.15 | 34.1 |
| *PtrCAld5H2* | < 0.001 | 0.39 | 4.08 ± 0.09 | 34.6 |
| *PtrAldOMT2* | < 0.001 | 0.34 | 104.73 ± 2.18 | 3391.9 |
| **Average** |  | **0.31** | **14.98 ± 5.15** |  |

R^2^ values represent variance explained by the regressions. ***β***_i_ are the slopes, which represent the increase in the number of protein molecules per molecule of transcript increased. One standard error of regression slopes is given. RMSE represents the root mean squared errors. The *p*-values of the F tests show that transcript abundance is a significant predictor explaining variation in protein abundance for different genes.

**Supplementary Table 3 │** Linear regression statistics for the integrative analysis predictability of lignin and wood properties.

| **Properties** | **Slope** | **RMSE** |
| --- | --- | --- |
| Lignin content | 0.99 | 1.665 |
| S/G ratio | 0.97 | 0.404 |
| S-subunits | 0.99 | 6.300 |
| G-subunits | 0.98 | 5.853 |
| H-subunits | 0.89 | 2.837 |
| *p*-Hydroxybenzoic acid | 0.97 | 1.125 |
| C:L ratio | 0.99 | 0.466 |
| Aldehydes | 0.84 | 0.316 |
| β–*O*–4 | 1.00 | 1.245 |
| β–5 | 0.96 | 1.064 |
| β–β | 0.99 | 0.547 |
| β–1 | 0.96 | 0.005 |
| End-groups | 0.98 | 0.821 |
| Plant height | 0.90 | 17.25 |
| Plant diameter | 0.90 | 0.066 |
| Stem volume | 0.90 | 22.78 |
| Glucose content | 1.00 | 3.453 |
| Xylose content | 0.99 | 1.487 |
| Total carbohydrate | 1.00 | 4.463 |
| Relative density | 0.99 | 0.027 |
| Modulus of elasticity | 0.98 | 493.0 |
| Glucose: unpretreated | 0.96 | 7.965 |
| Xylose: unpretreated | 0.93 | 5.084 |
| Glucose: pretreated | 0.99 | 6.403 |
| Xylose: pretreated | 0.98 | 8.830 |
| **Average** | **0.96** |  |

Slope represents the deviation from 1.00 of the slopes between the predicted and observed values. RMSE (root-mean-square-error) represents the estimate of the standard deviation of the random error.

**Supplementary Table 4│** Global sensitivity analysis.

| **Genes** | **Three most influenced lignin and wood properties (PRCC)** |
| --- | --- |
| *PtrPAL1* | Glucose: pretreated (-0.138); lignin content (0.107); C:L ratio (-0.100) |
| *PtrPAL2* | Glucose: pretreated (-0.175); xylose content (0.151); lignin content (0.151) |
| *PtrPAL3* | Xylose content (0.047) |
| *PtrPAL4\|5* | Glucose: pretreated (-0.358); lignin content (0.287); xylose: pretreated (-0.264) |
| *PtrC4H1* | β–1 (0.669); stem volume (-0.282); H-subunits (-0.272) |
| *PtrC4H2* | β–1 (0.405); lignin content (0.138); glucose: pretreated (-0.130) |
| *PtrC3H3* | Total sugar (-0.733); xylose content (-0.710); β–1 (0.669) |
| *Ptr4CL3* | G-subunits (0.235); stem volume (0.218); MOE (0.195) |
| *Ptr4CL5* | Total sugar (0.586); xylose content (0.581); S/G ratio (-0.497) |
| *PtrHCT1* | Height (0.407); xylose: pretreated (0.356); stem volume (0.264) |
| *PtrHCT6* | Height (0.600); diameter (0.568); glucose: pretreated (0.434) |
| *PtrCCoAOMT1* | Density (-0.341); *p*-hydroxybenzoic acid (-0.097); β–*O*–4 (0.088) |
| *PtrCCoAOMT2* | Density (-0.685); *p*-hydroxybenzoic acid (-0.244); β–*O*–4 (0.167) |
| *PtrCCoAOMT3* | Density (-0.101); β–*O*–4 (0.047) |
| *PtrCCR2* | Glucose (0.771); density (0.726); total sugar (0.705) |
| *PtrCAD1* | Xylose content (-0.332); aldehydes (-0.220); glucose (0.180) |
| *PtrCAD2* | β–1 (0.092); total sugar (-0.058); S/G ratio (-0.054) |
| *PtrCAld5H1* | End-groups (-0.603); G-subunits (-0.569); β–5 (-0.437) |
| *PtrCAld5H2* | End-groups (-0.550); G-subunits (-0.524); β–5 (-0.366) |
| *PtrAldOMT2* | End-groups (0.783); aldehydes (-0.705); G-subunits (0.680) |

Values denote PRCC (partial rank correlation coefficient), which represent the sensitivity of lignin and wood properties to changes in gene transcript abundances. Blue values indicate positive influence; red values indicate negative influence. All listed PRCC values are significantly different from zero (*p*<0.05).

**Supplementary References**

1. Higuchi, T. “Biosynthesis of Wood Components” in *Biochemistry and Molecular Biology of Wood* (Springer-Verlag, Berlin, Heidelberg, NY, 1997), pp. 93-262.
2. Boerjan, W., Ralph, J. & Baucher, M. Lignin biosynthesis. *Annu. Rev. Plant Biol.* **54**, 519-546 (2003).
3. Shi, R. *et al.* Regulation of phenylalanine ammonia-lyase (*PAL*) gene family in wood forming tissue of *Populus trichocarpa*. *Planta* **238**, 487-497 (2013).
4. Shi, R. *et al.* Towards a systems approach for lignin biosynthesis in *Populus trichocarpa*: Transcript abundance and specificity of the monolignol biosynthetic genes. *Plant Cell Physiol.* **51**, 144-163 (2010).
5. Higuchi, T. “Biosynthesis of Lignin” in *Biosynthesis and biodegradation of wood components* (Academic Press Inc., Orlando, FL, 1985), pp. 141-160.
6. Chen, H. *et al.* Membrane protein complexes catalyze both 4- and 3-hydroxylation of cinnamic acid derivatives in monolignol biosynthesis. *Proc. Natl. Acad. Sci. USA* **108**, 21253-21258 (2011).
7. Chen, H. *et al.* Systems biology of lignin biosynthesis in *Populus trichocarpa*: Heteromeric 4-coumaric acid:coenzyme A ligase protein complex formation, regulation, and numerical modeling. *Plant Cell* **26**, 876-893 (2014).
8. Hoffmann, L., Maury, S., Martz, F., Geoffroy, P. & Legrand, M. Purification, cloning, and properties of an acyltransferase controlling shikimate and quinate ester intermediates in phenylpropanoid metabolism. *J. Biol. Chem.* **278**, 95-103 (2003).
9. Ha, C. M. *et al.* An essential role of caffeoyl shikimate esterase in monolignol biosynthesis in *Medicago truncatula*. *Plant J.* **86,** 363-375 (2016).
10. Vargas, L. *et al.* Improving total saccharification yield of Arabidopsis plants by vessel-specific complementation of *caffeoyl shikimate esterase* (*cse*) mutants. *Biotechnol. Biofuels* **9**, 139 (2016).
11. Vanholme, R. *et al.* Caffeoyl shikimate esterase (CSE) is an enzyme in the lignin biosynthetic pathway in Arabidopsis. *Science* **341**, 1103-1106 (2013).
12. Wang, J. P. *et al.* Functional redundancy of the two 5-hydroxylases in monolignol biosynthesis of *Populus trichocarpa*: LC-MS/MS based protein quantification and metabolic-flux analysis. *Planta* **236**, 795-808 (2012).
13. Wang, J. P. *et al.* Complete proteomic-based enzyme reaction and inhibition kinetics reveal how monolignol biosynthetic enzyme families affect metabolic-flux and lignin in *Populus trichocarpa*. *Plant Cell* **26**, 894-914 (2014).
14. Osakabe, K. *et al.* Coniferyl aldehyde 5-hydroxylation and methylation direct syringyl lignin biosynthesis in angiosperms. *Proc. Natl. Acad. Sci. USA* **96**, 8955-8960 (1999).
15. Maier, T., Guell, M. & Serrano, L. Correlation of mRNA and protein in complex biological samples. *FEBS Lett.* **583**, 3966-3973 (2009).
16. Greenbaum, D., Colangelo, C., Williams, K. & Gerstein, M. Comparing protein abundance and mRNA expression levels on a genomic scale. *Genome Biol.* **4**, 117 (2003).
17. Shi, R., Yang, C., Lu, S., Sederoff, R. & Chiang, V. L. Specific down-regulation of *PAL* genes by artificial microRNAs in *Populus trichocarpa*. *Planta* **232**, 1281-1288 (2010).
18. Schmidt, M. *et al*. Label-free in situ imaging of lignification in the cell wall of low lignin transgenic *Populus trichocarpa*. *Planta* **230**, 589-597 (2009).
19. Lin, C. Y. *et al.* 4-Coumaroyl and caffeoyl shikimic acids inhibit 4-coumaric acid:coenzyme A ligases and modulate metabolic-flux for 3-hydroxylation in monolignol biosynthesis of *Populus trichocarpa*. *Mol. Plant* **8**, 176-187 (2015).
20. Wang, J. P. *et al*. “A proteomic based quantitative analysis of the relationship between monolignol biosynthetic protein abundance and lignin content using transgenic *Populus trichocarpa*” in *Recent Advances in Polyphenol Research Volume 5* (John Wiley & Sons, Ltd, Chichester, UK, 2017), pp. 89-107.
21. Dixon, R. A., Srinivasa Reddy, M. S. & Gallego-Giraldo, L. “Monolignol biosynthesis and its genetic manipulation: the good, the bad, and the ugly” in *Recent Advances in Polyphenol Research Volume 4* (John Wiley & Sons, Ltd, Chichester, UK, 2014), pp. 1-38.
22. Schwab, R., Ossowski, S., Riester, M., Warthmann, N. & Weigel, D. Highly specific gene silencing by artificial microRNAs in Arabidopsis. *Plant Cell* **18**, 1121-1133 (2006).
23. Vogel, C. & Marcotte, E. M. Insights into the regulation of protein abundance from proteomic and transcriptomic analyses. *Nat. Rev. Genet.* **13**, 227-232 (2012).
24. SEQC/MAQC-III Consortium. A comprehensive assessment of RNA-seq accuracy, reproducibility and information content by the Sequencing Quality Control Consortium. *Nat. Biotechnol.* **32**, 903-914 (2014).
25. Li, Q. *et al.* Splice variant of the SND1 transcription factor is a dominant negative of SND1 members and their regulation in *Populus trichocarpa*. *Proc. Natl. Acad. Sci. USA* **109**, 14699-14704 (2012).
26. McCarthy, D. J., Chen, Y. & Smyth, G. K. Differential expression analysis of multifactor RNA-Seq experiments with respect to biological variation. *Nucleic Acids Res.* **40**, 4288-4297 (2012).
27. Shuford, C. M. et al. Comprehensive quantification of monolignol-pathway enzymes in *Populus trichocarpa* by protein cleavage isotope dilution mass spectrometry. *J. Proteome Res.* **11**, 3390-3404 (2012).
28. Leek, J. T. *et al.* Tackling the widespread and critical impact of batch effects in high-throughput data. *Nat. Rev. Genet.* **11**, 733-739 (2010).
29. Qin, S., Kim, J., Arafat, D. & Gibson, G. Effect of normalization on statistical and biological interpretation of gene expression profiles. *Front. Genet.* **3**, 160 (2013).
30. de Sousa Abreu, R., Penalva, L. O., Marcotte, E. M. & Vogel, C. Global signatures of protein and mRNA expression levels. *Mol. Biosyst.* **5**, 1512-1526 (2009).
31. Ghaemmaghami, S. *et al.* Global analysis of protein expression in yeast. *Nature* **425**, 737-741 (2003).
32. Ishihama, Y. *et al.* Exponentially modified protein abundance index (emPAI) for estimation of absolute protein amount in proteomics by the number of sequenced peptides per protein. *Mol. Cell. Proteomics* **4**, 1265-1272 (2005).
33. Nie, L., Wu, G. & Zhang, W. Correlation of mRNA expression and protein abundance affected by multiple sequence features related to translational efficiency in *Desulfovibrio vulgaris*: A quantitative analysis. *Genetics* **174**, 2229-2243 (2006).
34. Schrimpf, S. P. *et al.* Comparative functional analysis of the *Caenorhabditis elegans* and *Drosophila melanogaster* proteomes. *PLoS Biol.* **7**, e1000048 (2009).
35. Vogel, C. *et al.* Sequence signatures and mRNA concentration can explain two‐thirds of protein abundance variation in a human cell line. *Mol. Syst. Biol.* **6**, 400 (2010).
36. Wang, J. P. *et al*. Phosphorylation is an on/off switch for 5-hydroxyconiferaldehyde O-methyltransferase activity in poplar monolignol biosynthesis. *Proc. Natl. Acad. Sci. USA* **112**, 8481-8486 (2015).
37. Loziuk, P. L., Hecht, E. S. & Muddiman, D. C. N-linked glycosite profiling and use of Skyline as a platform for characterization and relative quantification of glycans in differentiating xylem of *Populus trichocarpa*. *Anal. Bioanal. Chem*. **409,** 487-497 (2017).
38. Mansfield, S. D., Kim, H., Lu, F. & Ralph, J. Whole plant cell wall characterization using solution-state 2D NMR. *Nat. Protoc.* **7**, 1579-1589 (2012).
39. Ralph, J., Akiyama, T., Coleman, H. D. & Mansfield, S. D. Effects on lignin structure of coumarate 3-hydroxylase downregulation in poplar. *Bioenerg. Res.* **5**, 1009-1019 (2012).
40. Ralph, J. *et al.* Identification of the structure and origin of a thioacidolysis marker compound for ferulic acid incorporation into angiosperm lignins (and an indicator for cinnamoyl CoA reductase deficiency). *Plant J.* **53**, 368-379 (2008).
41. Ralph, J. *et al.* Effects of coumarate 3-hydroxylase down-regulation on lignin structure. *J. Biol. Chem.* **281**, 8843-8853 (2006).
42. Bowyer, J. L., Shmulsky, R. & Haygreen, J. G. in *Forest Products and Wood Science: An Introduction* (Wiley-Blackwell, Ames, Iowa, 1998).
43. Horvath, B., Li, L., Kasal, B., Peralta, P. & Peszlen, I. Effect of lignin genetic modification on wood anatomy of aspen trees. *IAWA J.* **31**, 29-38 (2010).
44. Rubinstein, R. Y. & Kroese, D. P. in *Simulation and the Monte Carlo method* (John Wiley & Sons, Hoboken, N.J., 2008).
45. McKay, M. D., Beckman, R. J. & Conover, W. J. Comparison of three methods for selecting values of input variables in the analysis of output from a computer code. *Technometrics* **21**, 239-245 (1979).
46. Mukaka, M. M. Statistics corner: A guide to appropriate use of correlation coefficient in medical research. *Malawi Med. J.* **24**, 69-71 (2012).
47. Chen, H. *et al.* Monolignol pathway 4-coumaric acid:coenzyme A ligases in *Populus trichocarpa*: Novel specificity, metabolic regulation, and simulation of coenzyme A ligation fluxes. *Plant Physiol.* **161**, 1501-1516 (2013).
