# Supplementary material for: Improving wood properties for wood utilization through multi-omics integration in lignin biosynthesis
Source: Nat Commun. 2018 Apr 20;9:1579. doi: 10.1038/s41467-018-03863-z (PMC5910405; doi:10.1038/s41467-018-03863-z)
Supplement: Supplementary file 2 — Description of Additional Supplementary Files [file 41467_2018_3863_MOESM2_ESM.pdf]

**Descriptions of Additional Supplementary Files**

File Name: Supplementary Data 1
Descriptions: Targeted transgene suppression in transgenic and wildtype plants

File Name: Supplementary Data 2
Descriptions: Integrative analysis equations

File Name: Supplementary Data 3
Descriptions: Wood composition of transgenic and wildtype P. trichocarpa

File Name: Supplementary Data 4
Descriptions: Lignin composition and linkages of transgenic and wildtype P. trichocarpa

File Name: Supplementary Data 5
Descriptions: Growth of the transgenic and wildtype P. trichocarpa

File Name: Supplementary Data 6
Descriptions: Modulus of elasticity of the transgenic and wildtype P. trichocarpa

File Name: Supplementary Data 7
Descriptions: Wood density of the transgenic and wildtype P. trichocarpa

File Name: Supplementary Data 8
Descriptions: Saccharification of the transgenic and wildtype P. trichocarpa

File Name: Supplementary Data 9
Descriptions: MATLAB codes for the integrative analysis

File Name: Supplementary Data 10
Descriptions: Simulations of the effects of monolignol gene perturbation on lignin and wood
