# Supplementary material for: Improving wood properties for wood utilization through multi-omics integration in lignin biosynthesis
Source: Nat Commun. 2018 Apr 20;9:1579. doi: 10.1038/s41467-018-03863-z (PMC5910405; doi:10.1038/s41467-018-03863-z)
Supplement: Supplementary file 3 — Supplementary Data 1 [file 41467_2018_3863_MOESM3_ESM.pdf]

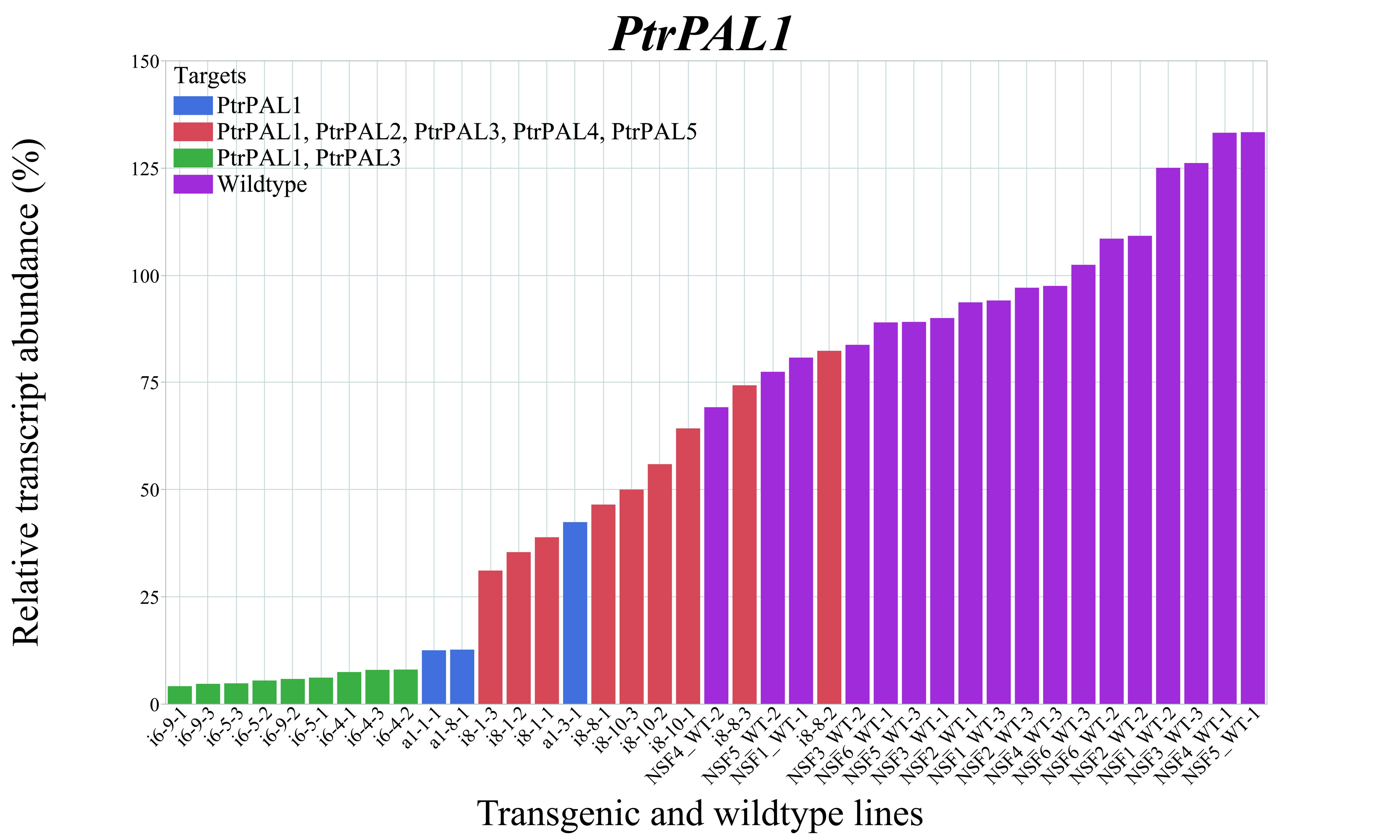
PtrPAL1
Relative transcript abundance (%)
150
125
100
75
50
25
0
Targets
PtrPAL1
PtrPAL1, PtrPAL2, PtrPAL3, PtrPAL4, PtrPAL5
PtrPAL1, PtrPAL3
Wildtype
i6-9-1
i6-9-3
i6-5-3
i6-5-2
i6-9-2
i6-5-1
i6-4-1
i6-4-3
i6-4-2
a1-1-1
a1-8-1
i8-1-3
i8-1-2
i8-1-1
a1-3-1
i8-8-1
i8-10-3
i8-10-2
i8-10-1
NSF4_WT-2
i8-8-3
NSF5_WT-2
NSF1_WT-1
i8-8-2
NSF3_WT-2
NSF6_WT-1
NSF5_WT-3
NSF3_WT-1
NSF2_WT-1
NSF1_WT-3
NSF2_WT-3
NSF4_WT-3
NSF6_WT-3
NSF6_WT-2
NSF2_WT-2
NSF1_WT-2
NSF3_WT-3
NSF4_WT-1
NSF5_WT-1
Transgenic and wildtype lines

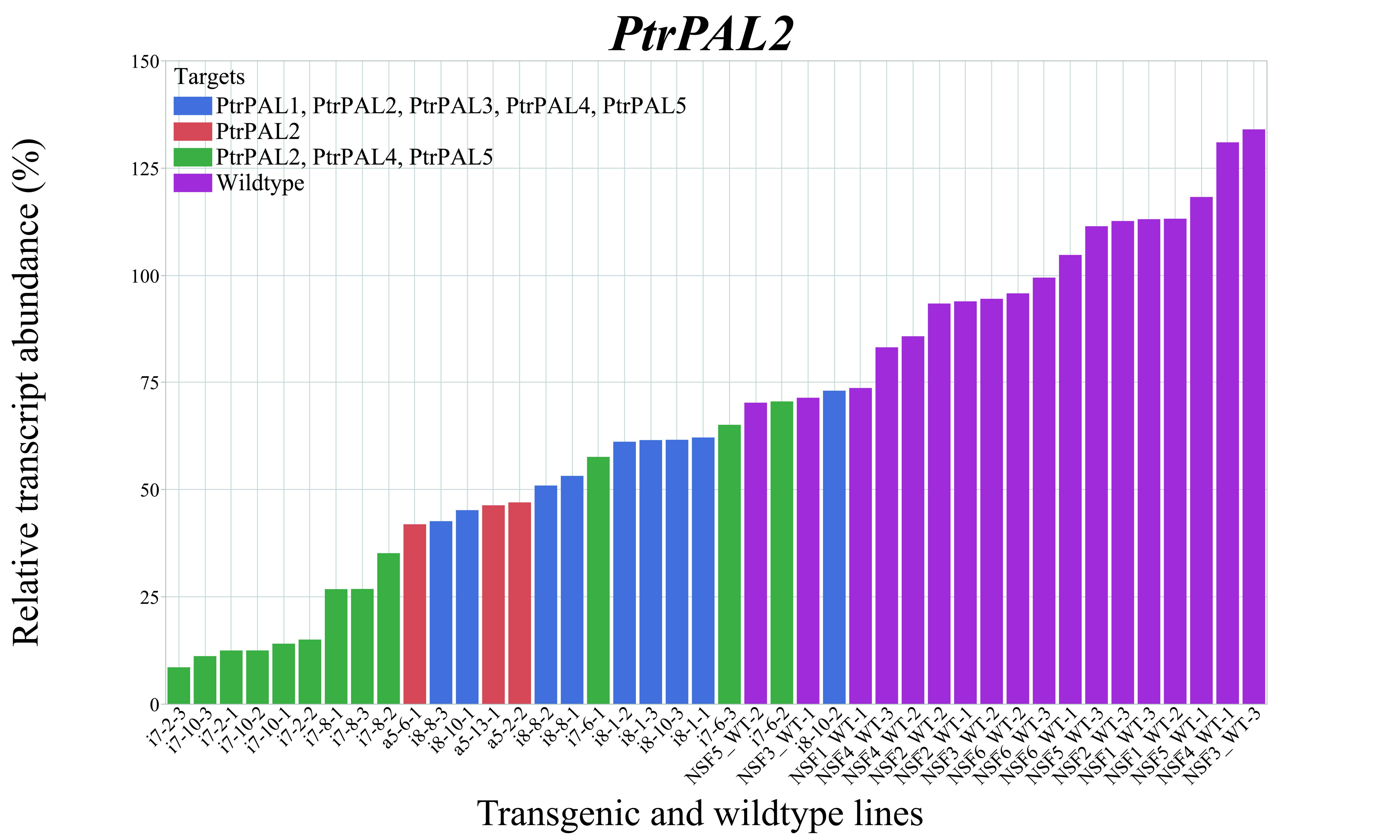

PtrPAL2
Targets
PtrPAL1, PtrPAL2, PtrPAL3, PtrPAL4, PtrPAL5
PtrPAL2
PtrPAL2, PtrPAL4, PtrPAL5
Wildtype
Relative transcript abundance (%)
0
25
50
75
100
125
150
i7-2-3
i7-10-3
i7-2-1
i7-10-2
i7-10-1
i7-2-2
i7-8-1
i7-8-3
i7-8-2
a5-6-1
i8-8-3
i8-10-1
a5-13-1
a5-2-2
i8-8-2
i8-8-1
i7-6-1
i8-1-2
i8-1-3
i8-10-3
i8-1-1
i7-6-3
NSF5_WT-2
i7-6-2
NSF3_WT-1
i8-10-2
NSF1_WT-1
NSF4_WT-3
NSF4_WT-2
NSF2_WT-2
NSF2_WT-1
NSF3_WT-2
NSF6_WT-2
NSF6_WT-3
NSF6_WT-1
NSF5_WT-3
NSF2_WT-3
NSF1_WT-3
NSF1_WT-2
NSF5_WT-1
NSF4_WT-1
NSF3_WT-3
Transgenic and wildtype lines

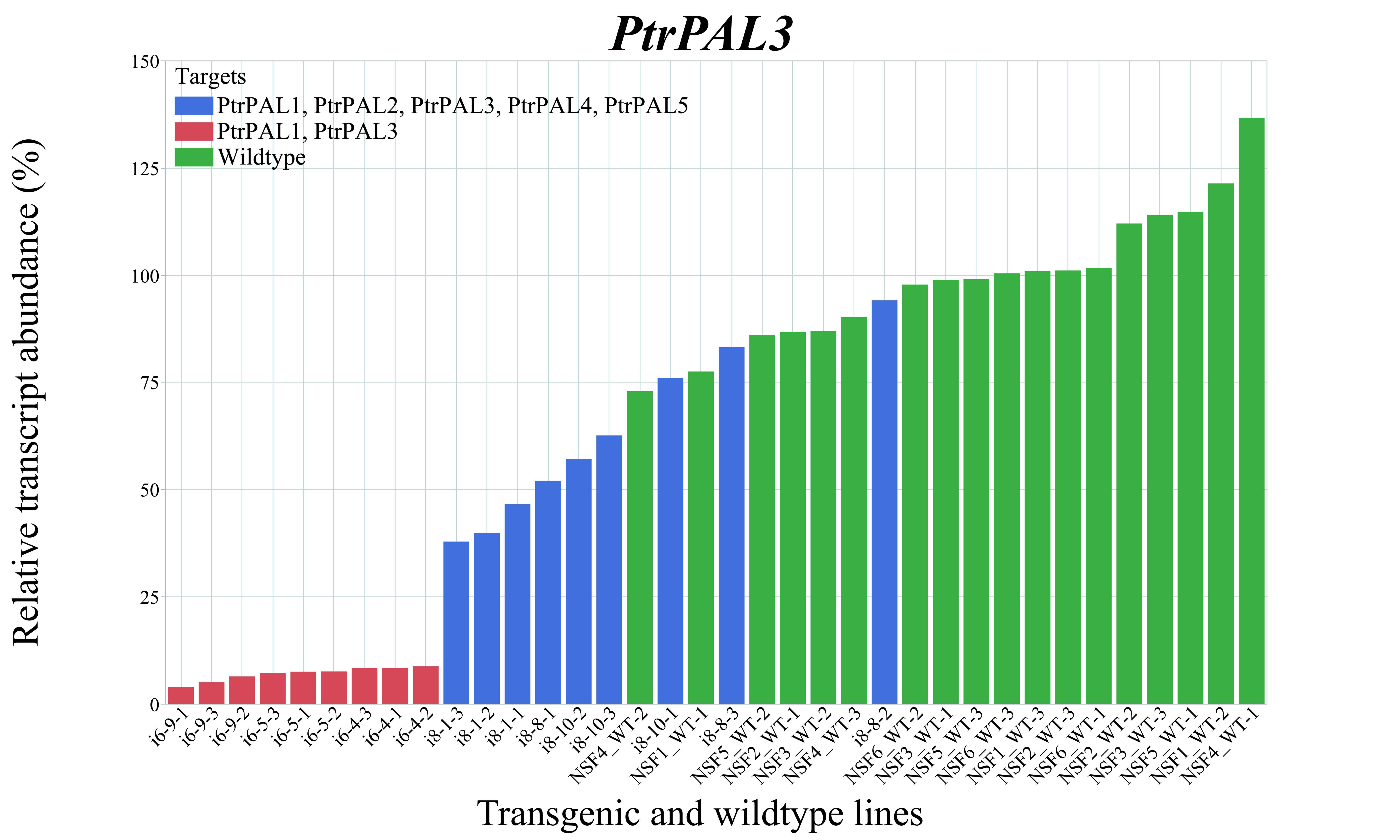
PtrPAL3
Targets
PtrPAL1, PtrPAL2, PtrPAL3, PtrPAL4, PtrPAL5
PtrPAL1, PtrPAL3
Wildtype
Relative transcript abundance (%)
0
25
50
75
100
125
150
i6-9-1
i6-9-3
i6-9-2
i6-5-3
i6-5-1
i6-5-2
i6-4-3
i6-4-1
i6-4-2
i8-1-3
i8-1-2
i8-1-1
i8-8-1
i8-10-2
i8-10-3
NSF4_WT-2
i8-10-1
NSF1_WT-1
i8-8-3
NSF5_WT-2
NSF2_WT-1
NSF3_WT-2
NSF4_WT-3
i8-8-2
NSF6_WT-2
NSF3_WT-1
NSF5_WT-3
NSF6_WT-3
NSF1_WT-3
NSF2_WT-3
NSF6_WT-1
NSF2_WT-2
NSF3_WT-3
NSF5_WT-1
NSF1_WT-2
NSF4_WT-1
Transgenic and wildtype lines

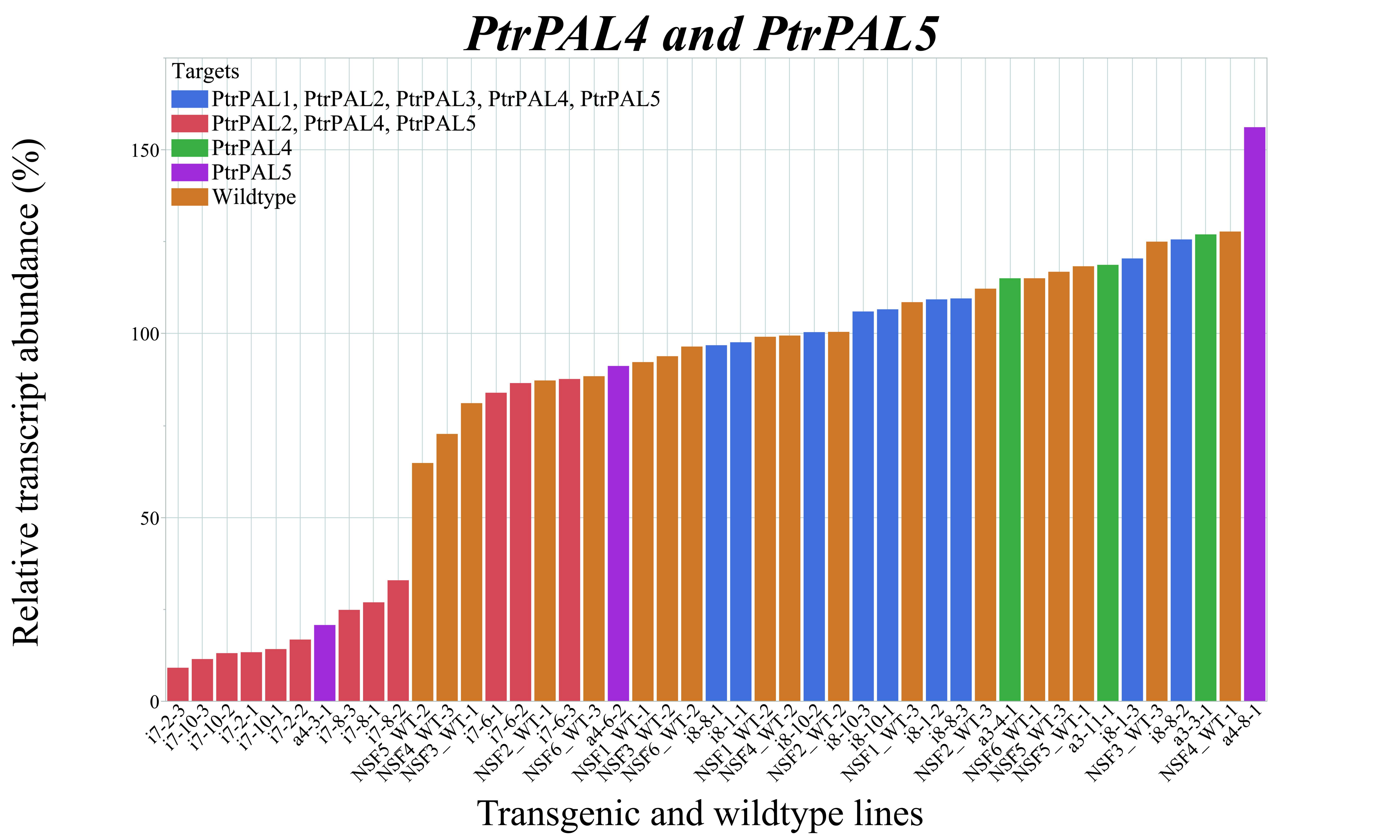
PtrPAL4 and PtrPAL5
Targets
PtrPAL1, PtrPAL2, PtrPAL3, PtrPAL4, PtrPAL5
PtrPAL2, PtrPAL4, PtrPAL5
PtrPAL4
PtrPAL5
Wildtype
Relative transcript abundance (%)
150
100
50
0
i7-2-3
i7-10-3
i7-10-2
i7-2-1
i7-10-1
i7-2-2
a4-3-1
i7-8-3
i7-8-1
i7-8-2
NSF5_WT-2
NSF4_WT-3
NSF3_WT-1
i7-6-1
i7-6-2
NSF2_WT-1
i7-6-3
NSF6_WT-3
a4-6-2
NSF1_WT-1
NSF3_WT-2
NSF6_WT-2
i8-8-1
i8-1-1
NSF1_WT-2
NSF4_WT-2
i8-10-2
NSF2_WT-2
i8-10-3
i8-10-1
NSF1_WT-3
i8-1-2
i8-8-3
NSF2_WT-3
a3-4-1
NSF6_WT-1
NSF5_WT-3
NSF5_WT-1
a3-11-1
i8-1-3
NSF3_WT-3
i8-8-2
a3-3-1
NSF4_WT-1
a4-8-1
Transgenic and wildtype lines

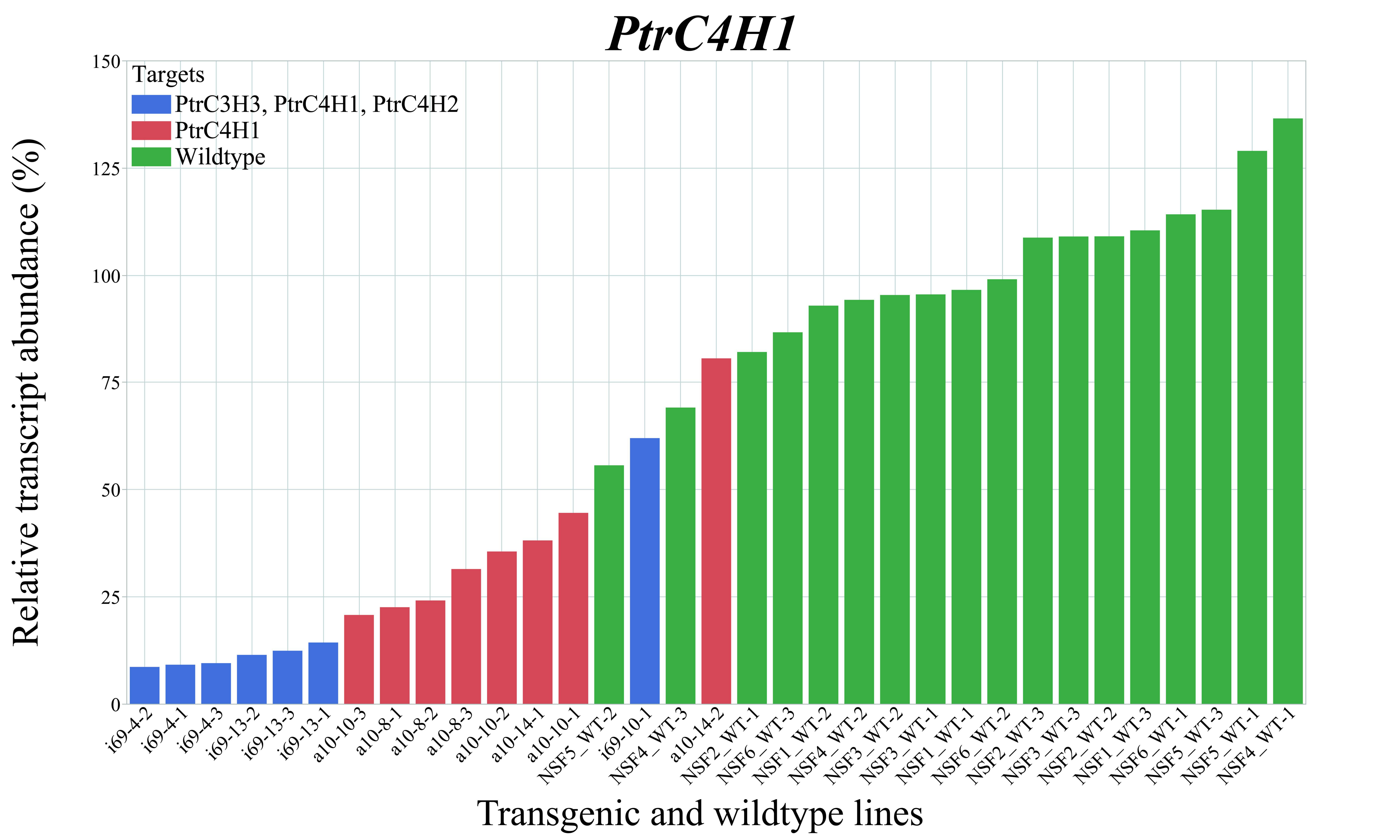

PtrC4H1
Targets
PtrC3H3, PtrC4H1, PtrC4H2
PtrC4H1
Wildtype
Relative transcript abundance (%)
150
125
100
75
50
25
0
i69-4-2
i69-4-1
i69-4-3
i69-13-2
i69-13-3
i69-13-1
a10-10-3
a10-8-1
a10-8-2
a10-8-3
a10-10-2
a10-14-1
a10-10-1
NSF5_WT-2
i69-10-1
NSF4_WT-3
a10-14-2
NSF2_WT-1
NSF6_WT-3
NSF1_WT-2
NSF4_WT-2
NSF3_WT-2
NSF3_WT-1
NSF1_WT-1
NSF6_WT-2
NSF2_WT-3
NSF3_WT-3
NSF2_WT-2
NSF1_WT-3
NSF6_WT-1
NSF5_WT-3
NSF5_WT-1
NSF4_WT-1
Transgenic and wildtype lines

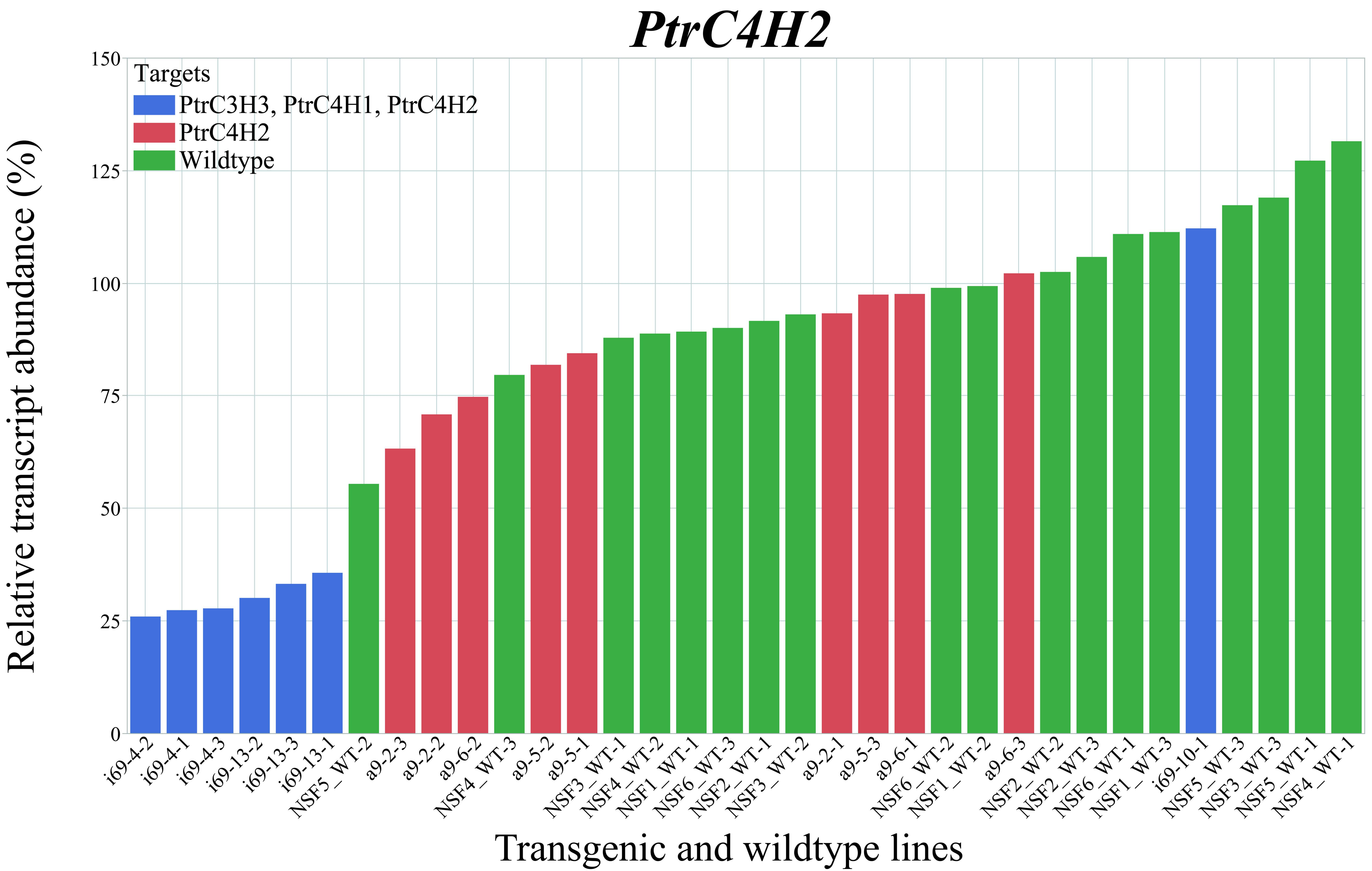
PtrC4H2
Targets
PtrC3H3, PtrC4H1, PtrC4H2
PtrC4H2
Wildtype
Relative transcript abundance (%)
0
25
50
75
100
125
150
i69-4-2
i69-4-1
i69-4-3
i69-13-2
i69-13-3
i69-13-1
NSF5_WT-2
a9-2-3
a9-2-2
a9-6-2
NSF4_WT-3
a9-5-2
a9-5-1
NSF3_WT-1
NSF4_WT-2
NSF1_WT-1
NSF6_WT-3
NSF2_WT-1
NSF3_WT-2
a9-2-1
a9-5-3
a9-6-1
NSF6_WT-2
NSF1_WT-2
a9-6-3
NSF2_WT-2
NSF2_WT-3
NSF6_WT-1
NSF1_WT-3
i69-10-1
NSF5_WT-3
NSF3_WT-3
NSF5_WT-1
NSF4_WT-1
Transgenic and wildtype lines

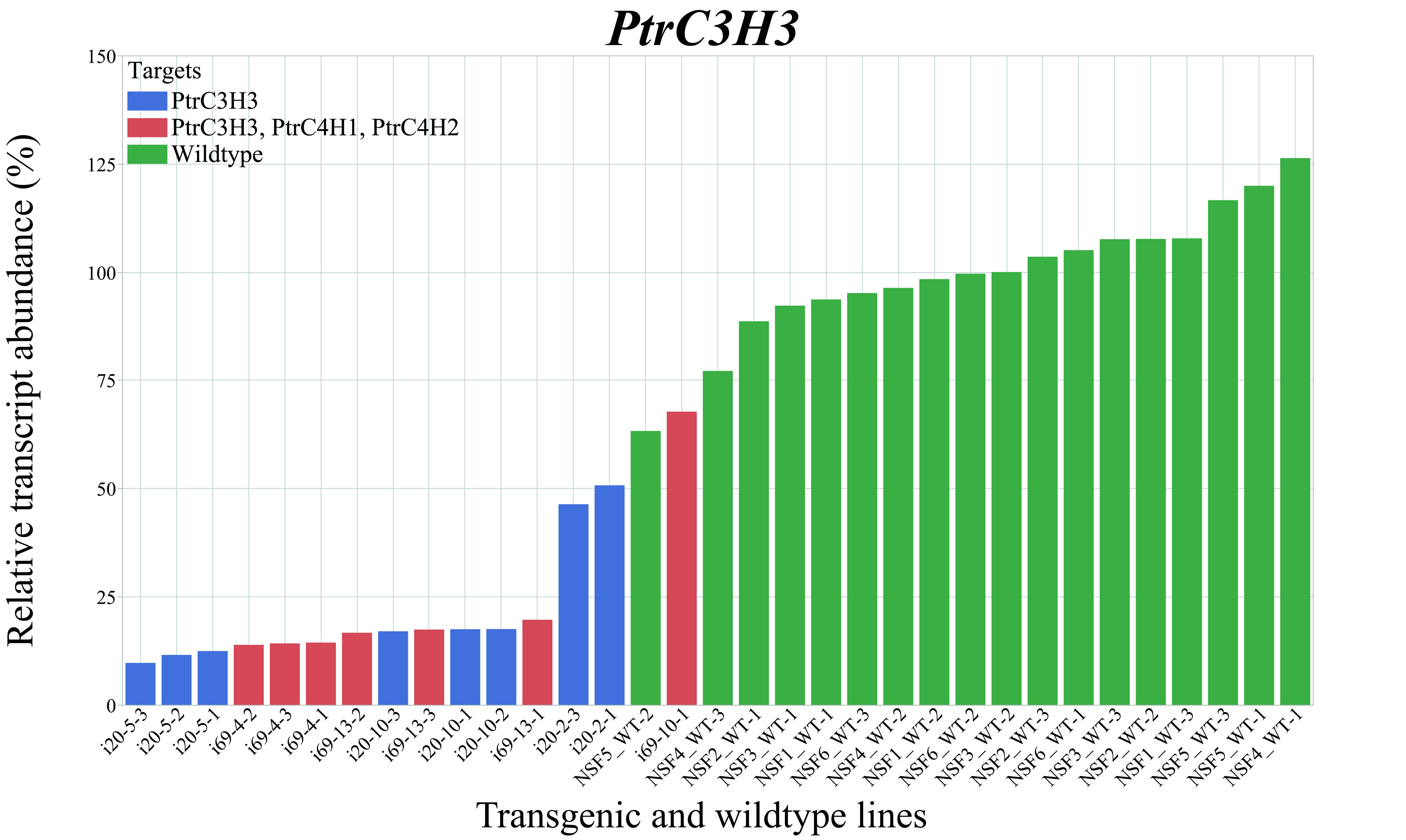

PtrC3H3
Relative transcript abundance (%)
150
125
100
75
50
25
0
Targets
PtrC3H3
PtrC3H3, PtrC4H1, PtrC4H2
Wildtype
i20-5-3
i20-5-2
i20-5-1
i69-4-2
i69-4-3
i69-4-1
i69-13-2
i20-10-3
i69-13-3
i20-10-1
i20-10-2
i69-13-1
i20-2-3
i20-2-1
NSF5_WT-2
i69-10-1
NSF4_WT-3
NSF2_WT-1
NSF3_WT-1
NSF1_WT-1
NSF6_WT-3
NSF4_WT-2
NSF1_WT-2
NSF6_WT-2
NSF3_WT-2
NSF2_WT-3
NSF6_WT-1
NSF3_WT-3
NSF2_WT-2
NSF1_WT-3
NSF5_WT-3
NSF5_WT-1
NSF4_WT-1
Transgenic and wildtype lines

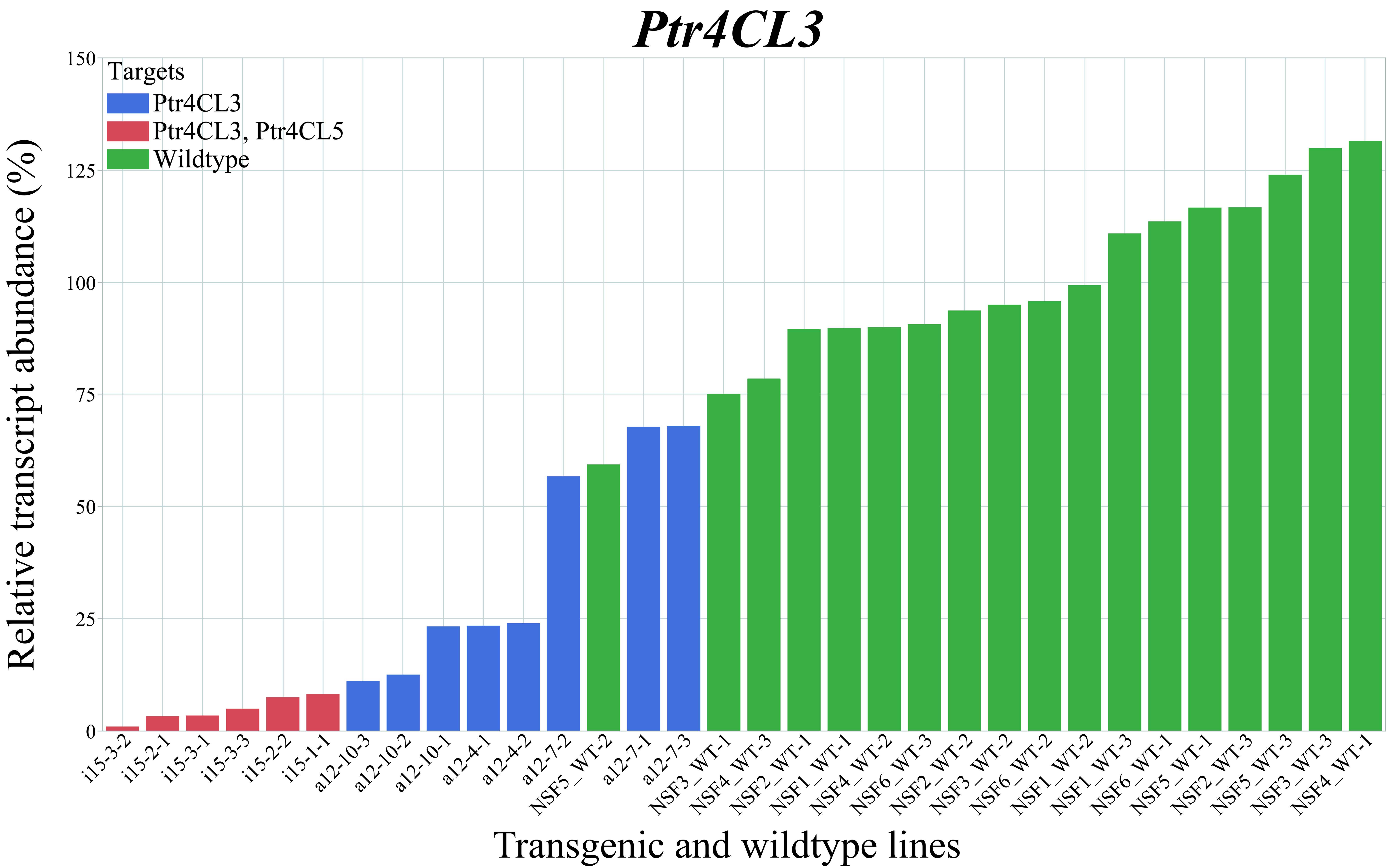
Ptr4CL3
Targets
Ptr4CL3
Ptr4CL3, Ptr4CL5
Wildtype
Relative transcript abundance (%)
0
25
50
75
100
125
150
i15-3-2
i15-2-1
i15-3-1
i15-3-3
i15-2-2
i15-1-1
a12-10-3
a12-10-2
a12-10-1
a12-4-1
a12-4-2
a12-7-2
NSF5_WT-2
a12-7-1
a12-7-3
NSF3_WT-1
NSF4_WT-3
NSF2_WT-1
NSF1_WT-1
NSF4_WT-2
NSF6_WT-3
NSF2_WT-2
NSF3_WT-2
NSF6_WT-2
NSF1_WT-2
NSF1_WT-3
NSF6_WT-1
NSF5_WT-1
NSF2_WT-3
NSF5_WT-3
NSF3_WT-3
NSF4_WT-1
Transgenic and wildtype lines

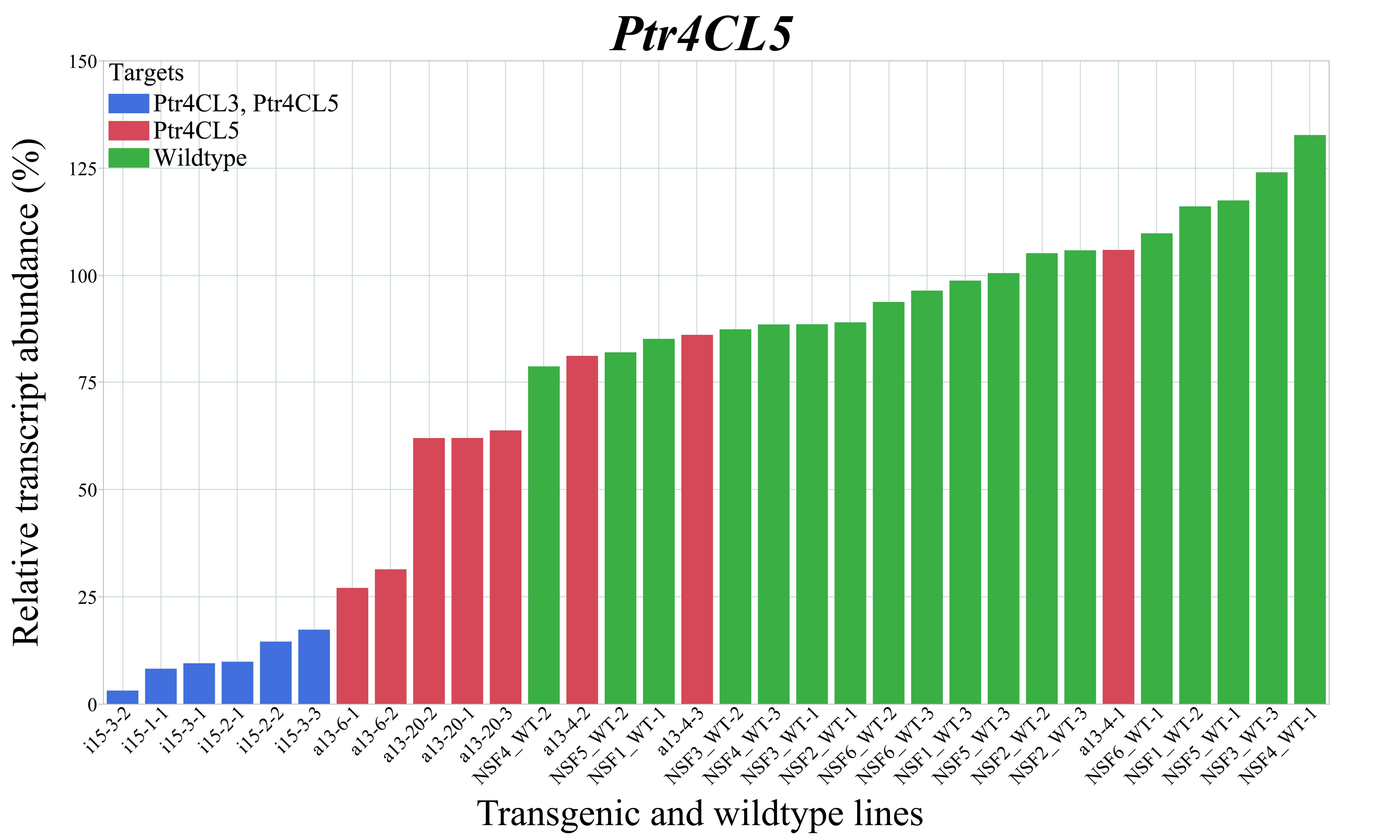

Ptr4CL5
Targets
Ptr4CL3, Ptr4CL5
Ptr4CL5
Wildtype
Relative transcript abundance (%)
0
25
50
75
100
125
150
i15-3-2
i15-1-1
i15-3-1
i15-2-1
i15-2-2
i15-3-3
a13-6-1
a13-6-2
a13-20-2
a13-20-1
a13-20-3
NSF4_WT-2
a13-4-2
NSF5_WT-2
NSF1_WT-1
a13-4-3
NSF3_WT-2
NSF4_WT-3
NSF3_WT-1
NSF2_WT-1
NSF6_WT-2
NSF6_WT-3
NSF1_WT-3
NSF5_WT-3
NSF2_WT-2
NSF2_WT-3
a13-4-1
NSF6_WT-1
NSF1_WT-2
NSF5_WT-1
NSF3_WT-3
NSF4_WT-1
Transgenic and wildtype lines

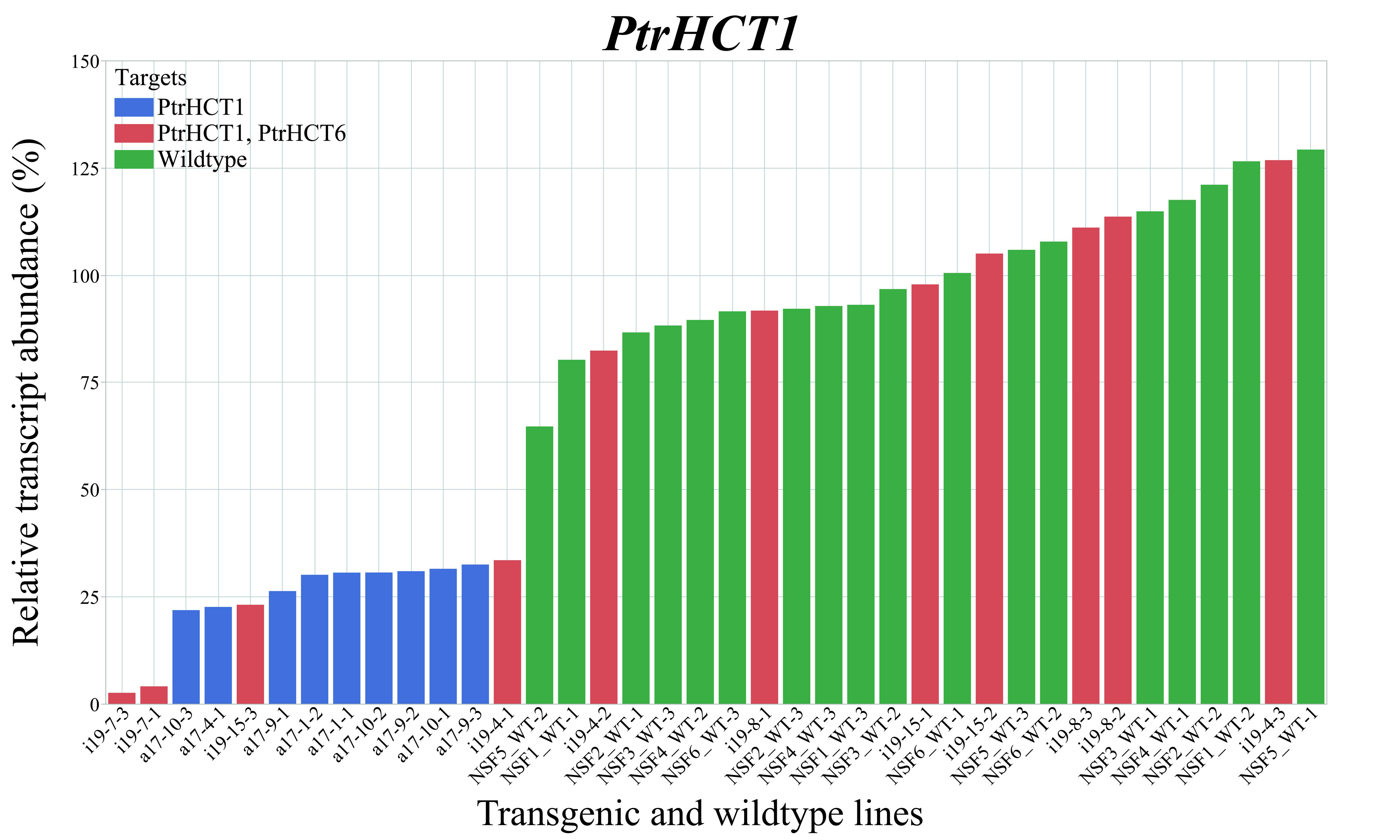

PtrHCT1
Relative transcript abundance (%)
150
125
100
75
50
25
0
Targets
PtrHCT1
PtrHCT1, PtrHCT6
Wildtype
i19-7-3
i19-7-1
a17-10-3
a17-4-1
i19-15-3
a17-9-1
a17-1-2
a17-1-1
a17-10-2
a17-9-2
a17-10-1
a17-9-3
i19-4-1
NSF5_WT-2
NSF1_WT-1
i19-4-2
NSF2_WT-1
NSF3_WT-3
NSF4_WT-2
NSF6_WT-3
i19-8-1
NSF2_WT-3
NSF4_WT-3
NSF1_WT-3
NSF3_WT-2
i19-15-1
NSF6_WT-1
i19-15-2
NSF5_WT-3
NSF6_WT-2
i19-8-3
i19-8-2
NSF3_WT-1
NSF4_WT-1
NSF2_WT-2
NSF1_WT-2
i19-4-3
NSF5_WT-1
Transgenic and wildtype lines

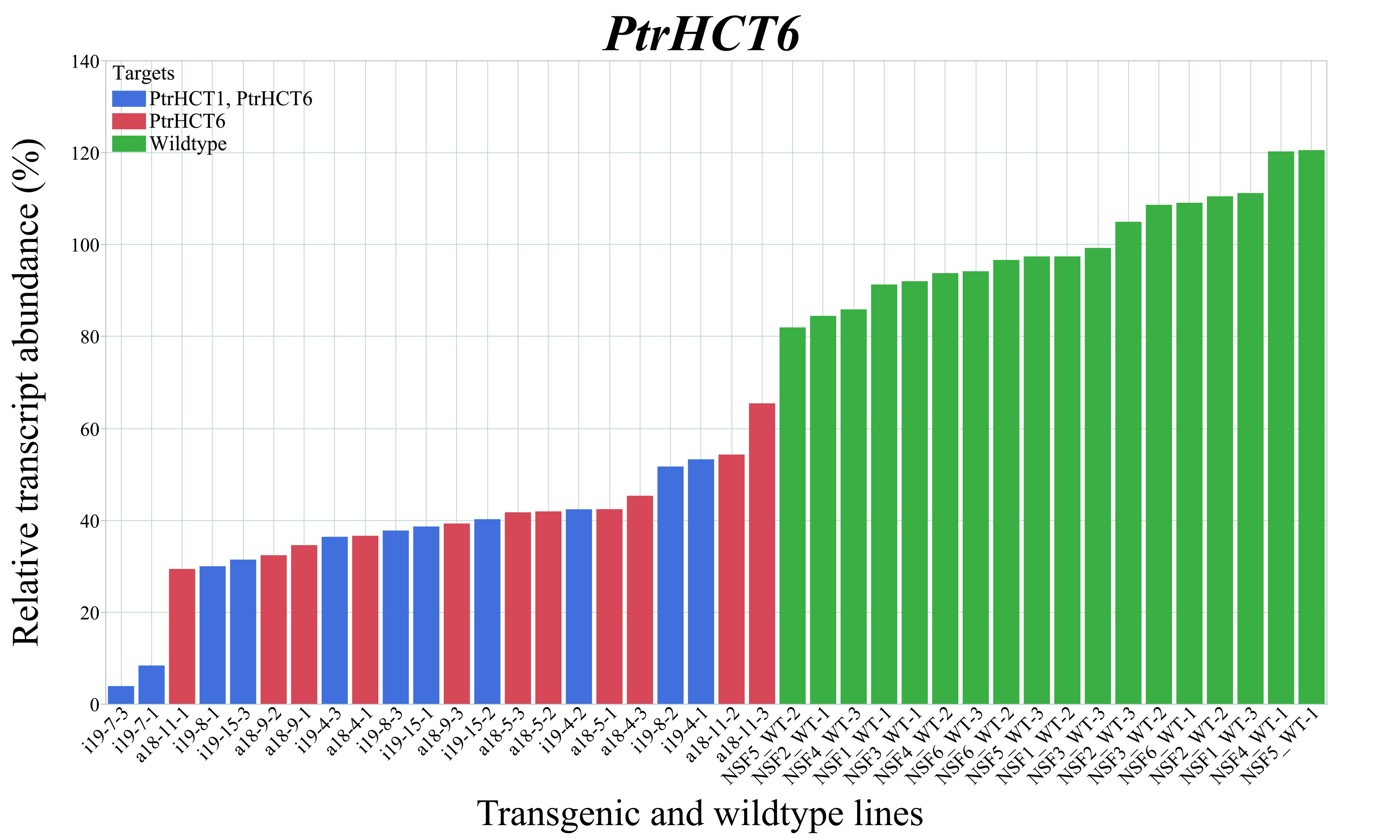

PtrHCT6
Relative transcript abundance (%)
Transgenic and wildtype lines
Targets
PtrHCT1, PtrHCT6
PtrHCT6
Wildtype
0
20
40
60
80
100
120
140
i19-7-3
i19-7-1
a18-11-1
i19-8-1
i19-15-3
a18-9-2
a18-9-1
i19-4-3
a18-4-1
i19-8-3
i19-15-1
a18-9-3
i19-15-2
a18-5-3
a18-5-2
i19-4-2
a18-5-1
a18-4-3
i19-8-2
i19-4-1
a18-11-2
a18-11-3
NSF5_WT-2
NSF2_WT-1
NSF4_WT-3
NSF1_WT-1
NSF3_WT-1
NSF4_WT-2
NSF6_WT-3
NSF6_WT-2
NSF5_WT-3
NSF1_WT-2
NSF3_WT-3
NSF2_WT-3
NSF3_WT-2
NSF6_WT-1
NSF2_WT-2
NSF1_WT-3
NSF4_WT-1
NSF5_WT-1

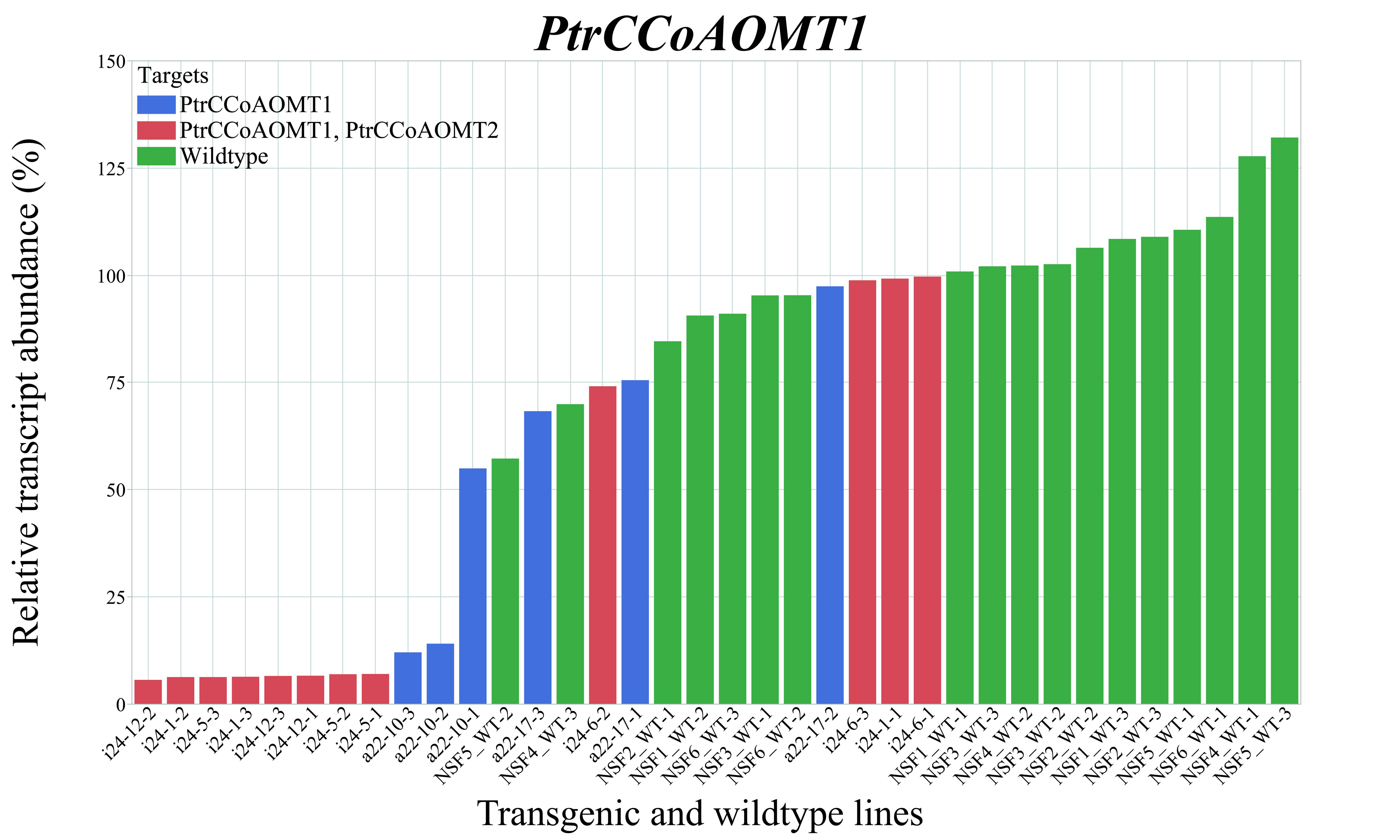

PtrCCoAOMT1
Targets
PtrCCoAOMT1
PtrCCoAOMT1, PtrCCoAOMT2
Wildtype
Relative transcript abundance (%)
0
25
50
75
100
125
150
i24-12-2
i24-1-2
i24-5-3
i24-1-3
i24-12-3
i24-12-1
i24-5-2
i24-5-1
a22-10-3
a22-10-2
a22-10-1
NSF5_WT-2
a22-17-3
NSF4_WT-3
i24-6-2
a22-17-1
NSF2_WT-1
NSF1_WT-2
NSF6_WT-3
NSF3_WT-1
NSF6_WT-2
a22-17-2
i24-6-3
i24-1-1
i24-6-1
NSF1_WT-1
NSF3_WT-3
NSF4_WT-2
NSF3_WT-2
NSF2_WT-2
NSF1_WT-3
NSF2_WT-3
NSF5_WT-1
NSF6_WT-1
NSF4_WT-1
NSF5_WT-3
Transgenic and wildtype lines

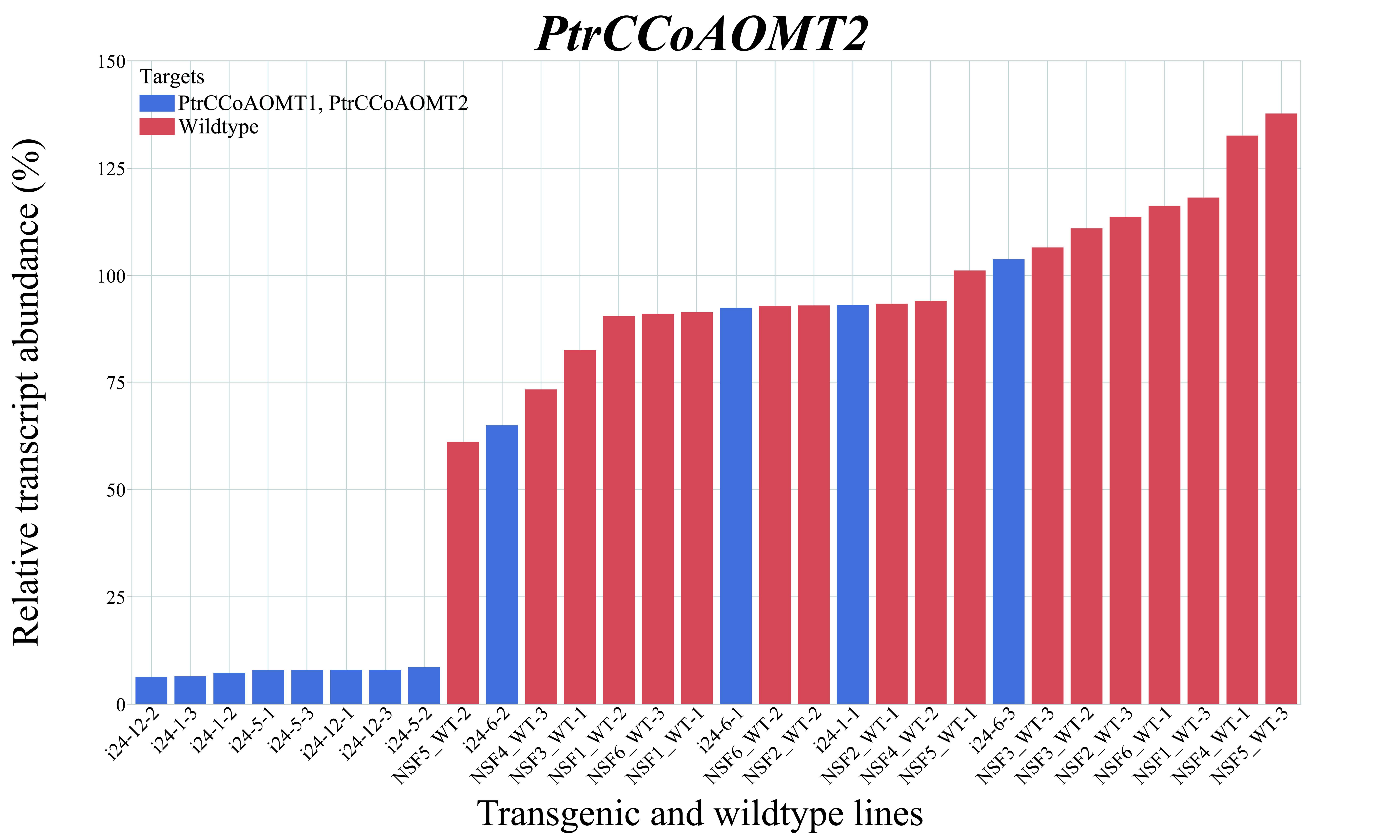
PtrCCoAOMT2
Targets
PtrCCoAOMT1, PtrCCoAOMT2
Wildtype
Relative transcript abundance (%)
0
25
50
75
100
125
150
i24-12-2
i24-1-3
i24-1-2
i24-5-1
i24-5-3
i24-12-1
i24-12-3
i24-5-2
NSF5_WT-2
i24-6-2
NSF4_WT-3
NSF3_WT-1
NSF1_WT-2
NSF6_WT-3
NSF1_WT-1
i24-6-1
NSF6_WT-2
NSF2_WT-2
i24-1-1
NSF2_WT-1
NSF4_WT-2
NSF5_WT-1
i24-6-3
NSF3_WT-3
NSF3_WT-2
NSF2_WT-3
NSF6_WT-1
NSF1_WT-3
NSF4_WT-1
NSF5_WT-3
Transgenic and wildtype lines

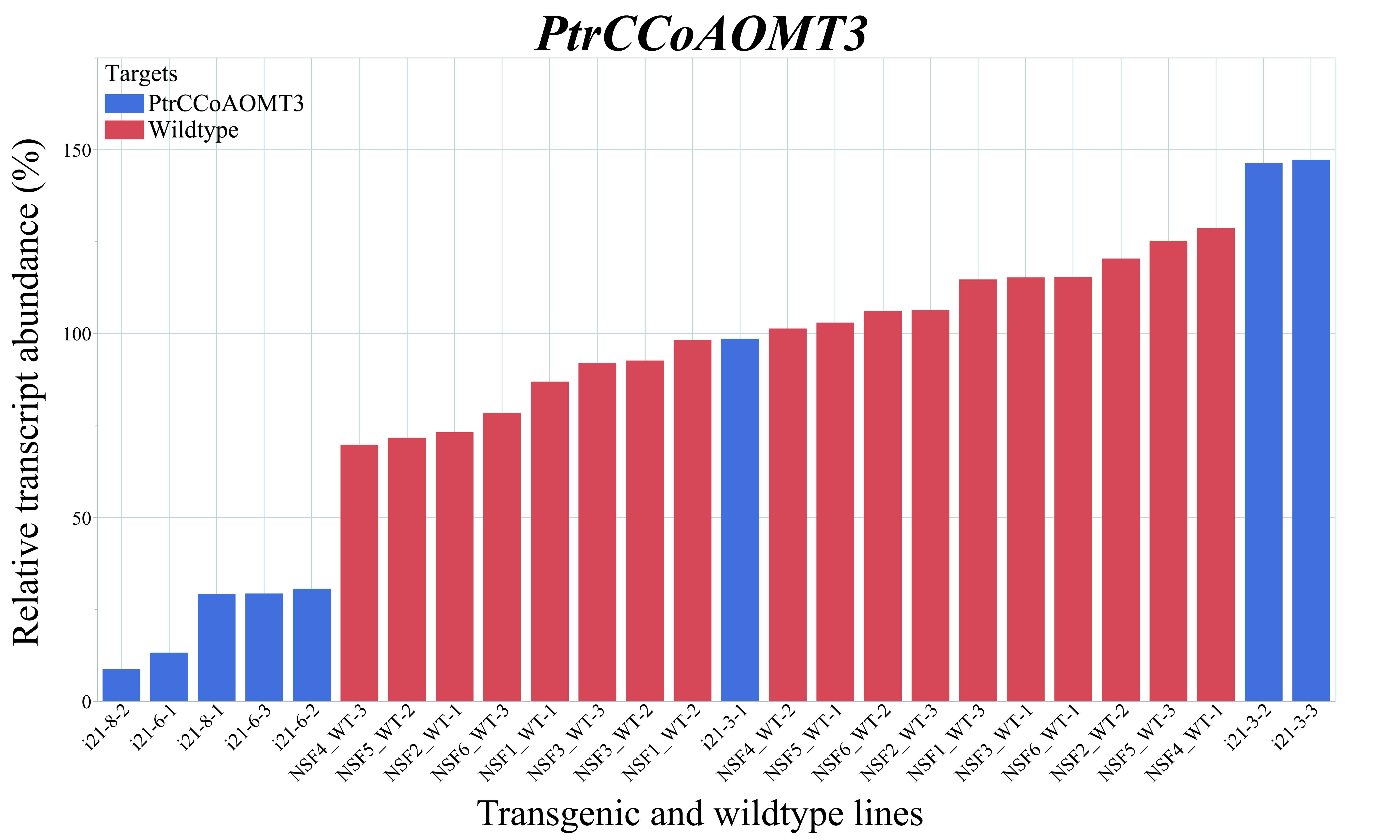
PtrCCoAOMT3
Targets
PtrCCoAOMT3
Wildtype
Relative transcript abundance (%)
0
50
100
150
i21-8-2
i21-6-1
i21-8-1
i21-6-3
i21-6-2
NSF4_WT-3
NSF5_WT-2
NSF2_WT-1
NSF6_WT-3
NSF1_WT-1
NSF3_WT-3
NSF3_WT-2
NSF1_WT-2
i21-3-1
NSF4_WT-2
NSF5_WT-1
NSF6_WT-2
NSF2_WT-3
NSF1_WT-3
NSF3_WT-1
NSF6_WT-1
NSF2_WT-2
NSF5_WT-3
NSF4_WT-1
i21-3-2
i21-3-3
Transgenic and wildtype lines

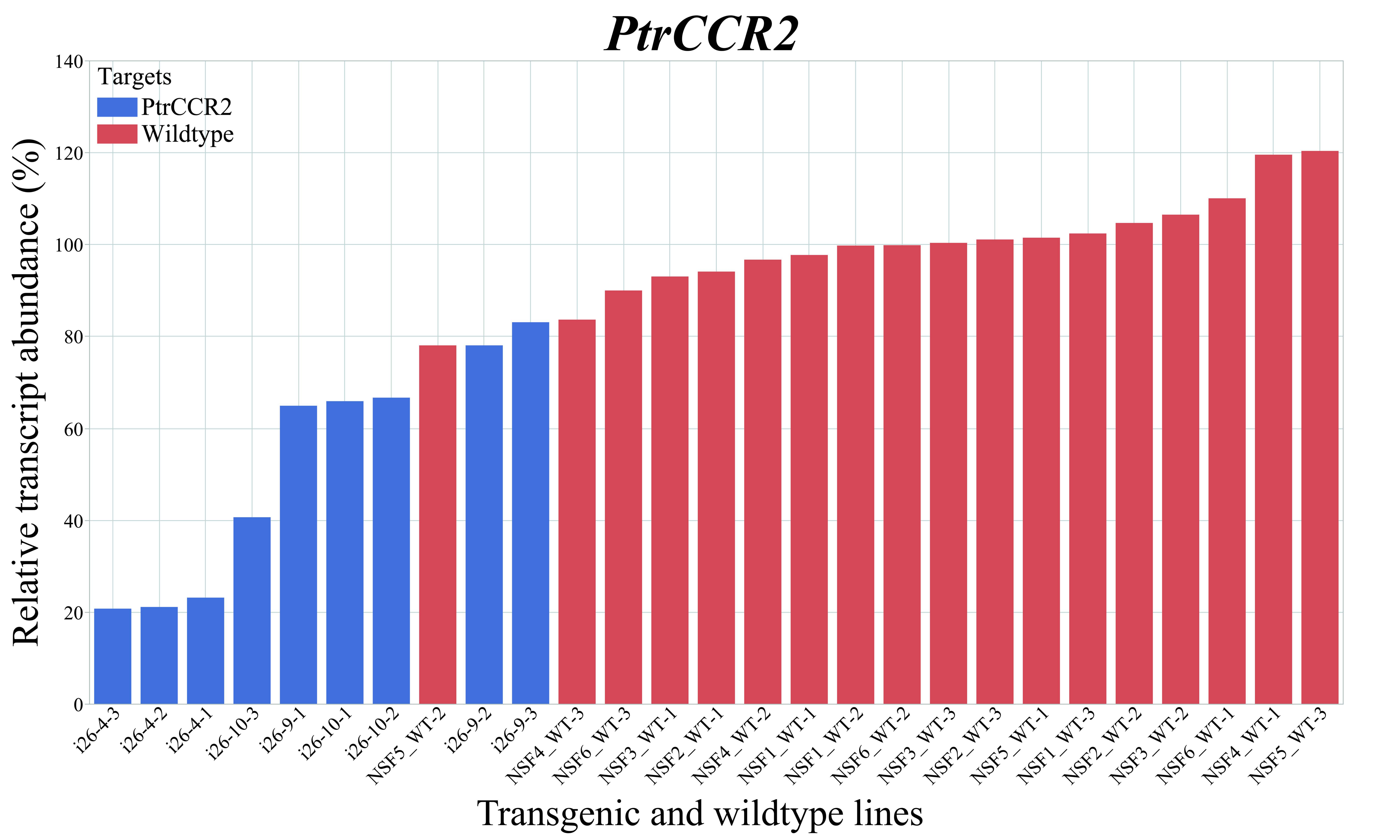
PtrCCR2
Targets
PtrCCR2
Wildtype
Relative transcript abundance (%)
0
20
40
60
80
100
120
140
i26-4-3
i26-4-2
i26-4-1
i26-10-3
i26-9-1
i26-10-1
i26-10-2
NSF5_WT-2
i26-9-2
i26-9-3
NSF4_WT-3
NSF6_WT-3
NSF3_WT-1
NSF2_WT-1
NSF4_WT-2
NSF1_WT-1
NSF1_WT-2
NSF6_WT-2
NSF3_WT-3
NSF2_WT-3
NSF5_WT-1
NSF1_WT-3
NSF2_WT-2
NSF3_WT-2
NSF6_WT-1
NSF4_WT-1
NSF5_WT-3
Transgenic and wildtype lines

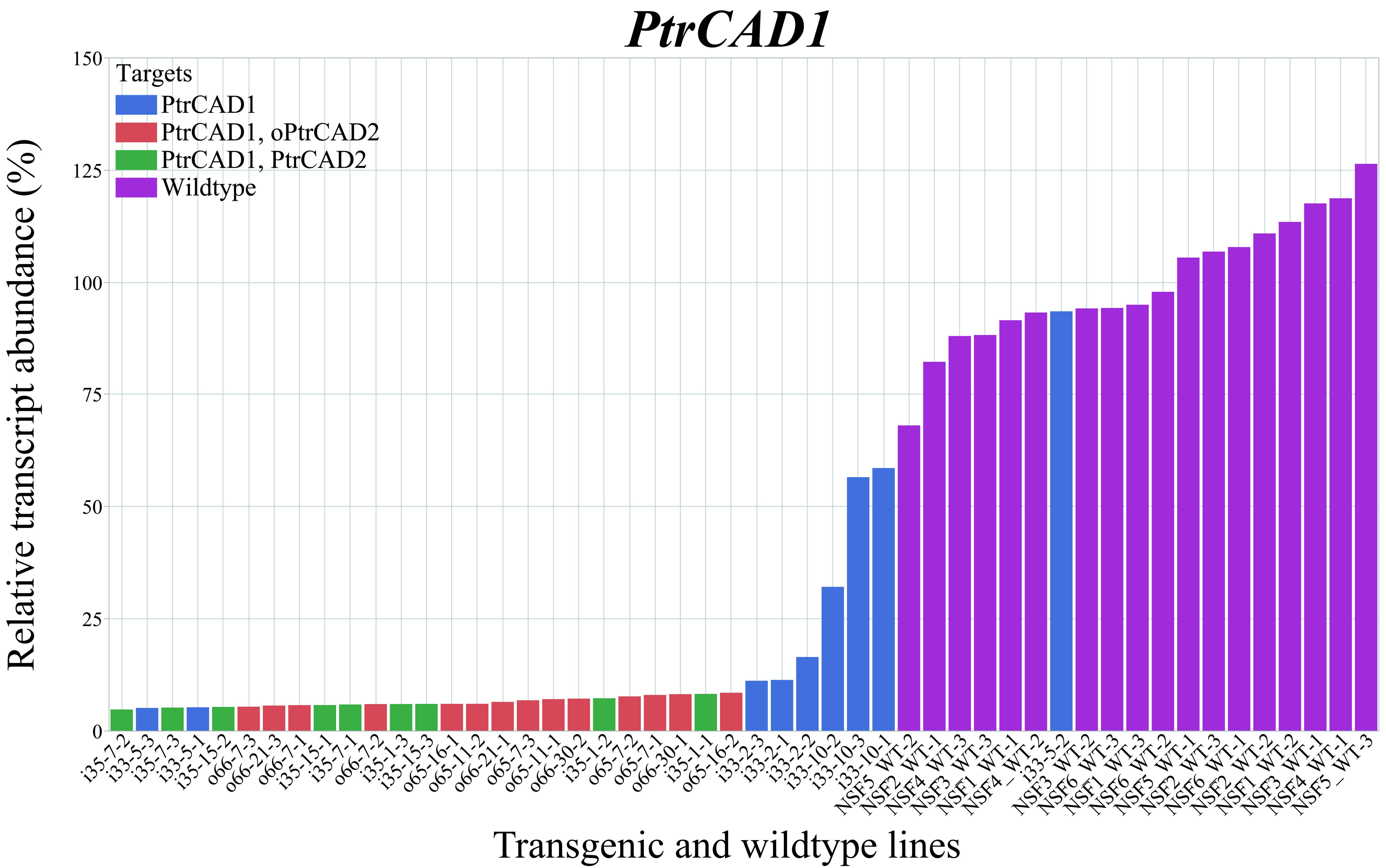
PtrCAD1
Targets
PtrCAD1
PtrCAD1, oPtrCAD2
PtrCAD1, PtrCAD2
Wildtype
Relative transcript abundance (%)
0
25
50
75
100
125
150
i35-7-2
i33-5-3
i35-7-3
i33-5-1
i35-15-2
o66-7-3
o66-21-3
o66-7-1
i35-15-1
i35-7-1
o66-7-2
i35-1-3
i35-15-3
o65-16-1
o65-11-2
o66-21-1
o65-7-3
o65-11-1
o66-30-2
i35-1-2
o65-7-2
o65-7-1
o66-30-1
i35-1-1
o65-16-2
i33-2-3
i33-2-1
i33-2-2
i33-10-2
i33-10-3
i33-10-1
NSF5_WT-2
NSF2_WT-1
NSF4_WT-3
NSF3_WT-3
NSF1_WT-1
NSF4_WT-2
i33-5-2
NSF3_WT-2
NSF6_WT-3
NSF1_WT-3
NSF6_WT-2
NSF5_WT-1
NSF2_WT-3
NSF6_WT-1
NSF2_WT-2
NSF1_WT-2
NSF3_WT-1
NSF4_WT-1
NSF5_WT-3
Transgenic and wildtype lines

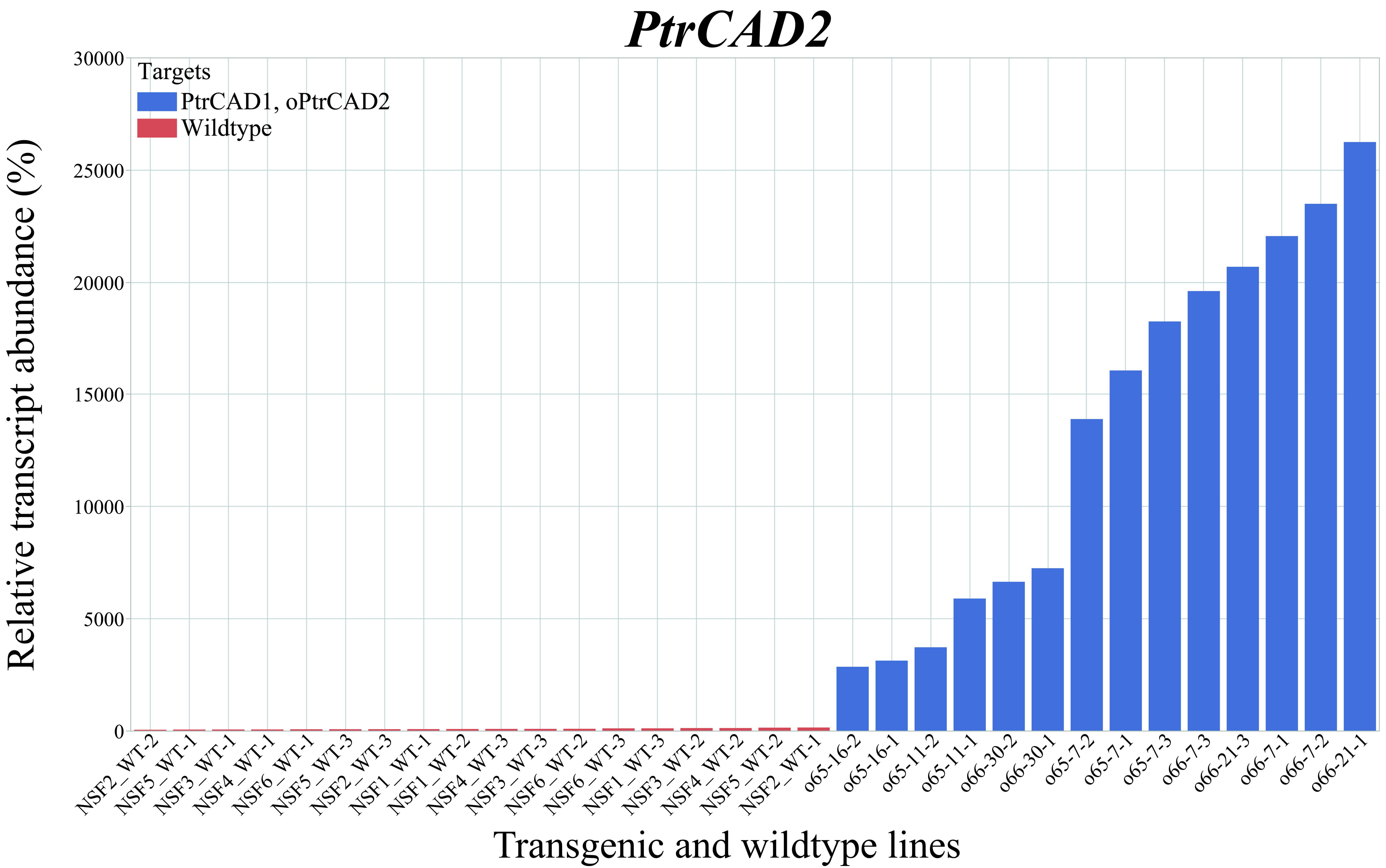
PtrCAD2
Targets
PtrCAD1, oPtrCAD2
Wildtype
Relative transcript abundance (%)
30000
25000
20000
15000
10000
5000
0
NSF2_WT-2
NSF5_WT-1
NSF3_WT-1
NSF4_WT-1
NSF6_WT-1
NSF5_WT-3
NSF2_WT-3
NSF1_WT-1
NSF1_WT-2
NSF4_WT-3
NSF3_WT-3
NSF6_WT-2
NSF6_WT-3
NSF1_WT-3
NSF3_WT-2
NSF4_WT-2
NSF5_WT-2
NSF2_WT-1
o65-16-2
o65-16-1
o65-11-2
o65-11-1
o66-30-2
o66-30-1
o65-7-2
o65-7-1
o65-7-3
o66-7-3
o66-21-3
o66-7-1
o66-7-2
o66-21-1
Transgenic and wildtype lines

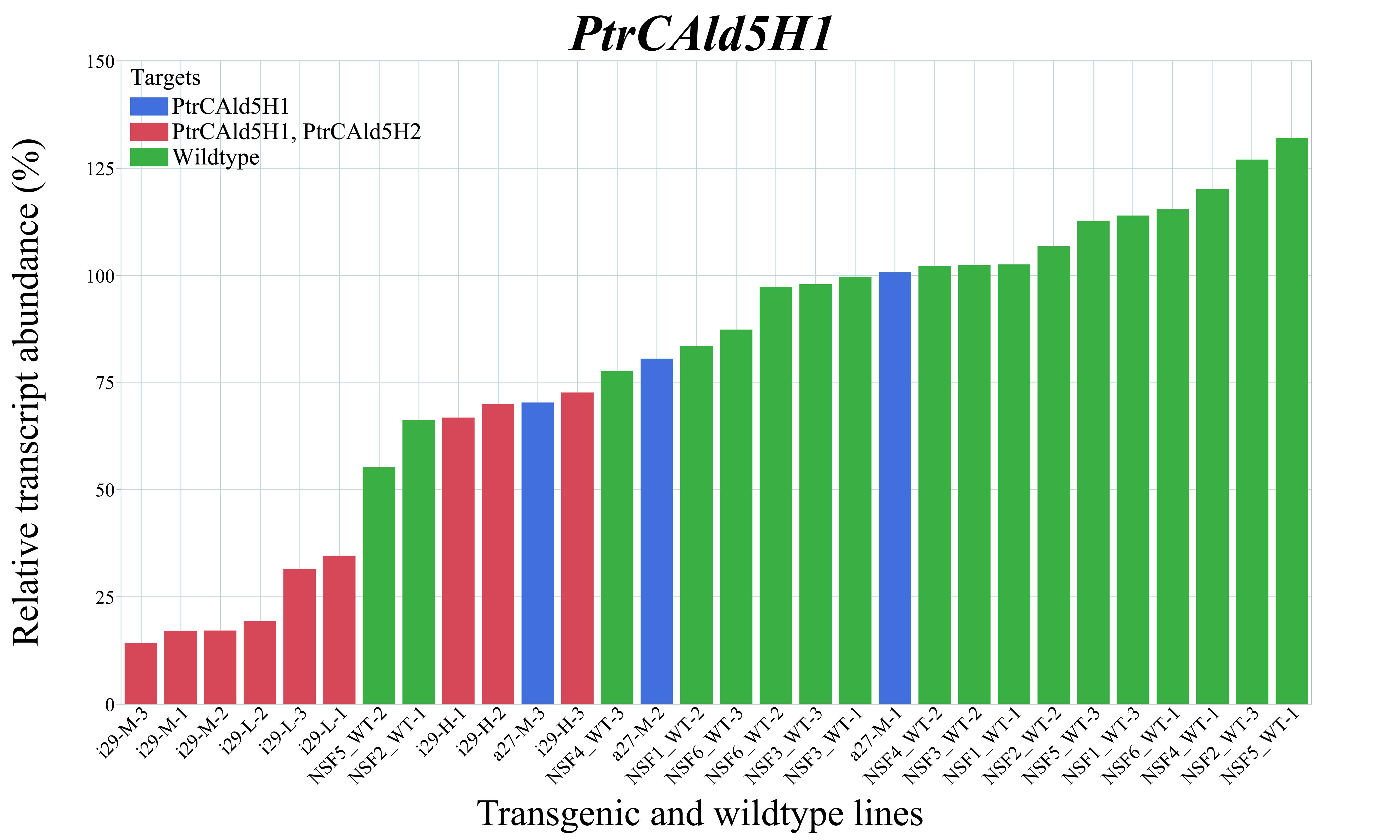
PtrCAld5H1
Relative transcript abundance (%)
150
125
100
75
50
25
0
Targets
PtrCAld5H1
PtrCAld5H1, PtrCAld5H2
Wildtype
i29-M-3
i29-M-1
i29-M-2
i29-L-2
i29-L-3
i29-L-1
NSF5_WT-2
NSF2_WT-1
i29-H-1
i29-H-2
a27-M-3
i29-H-3
NSF4_WT-3
a27-M-2
NSF1_WT-2
NSF6_WT-3
NSF6_WT-2
NSF3_WT-3
NSF3_WT-1
a27-M-1
NSF4_WT-2
NSF3_WT-2
NSF1_WT-1
NSF2_WT-2
NSF5_WT-3
NSF1_WT-3
NSF6_WT-1
NSF4_WT-1
NSF2_WT-3
NSF5_WT-1
Transgenic and wildtype lines

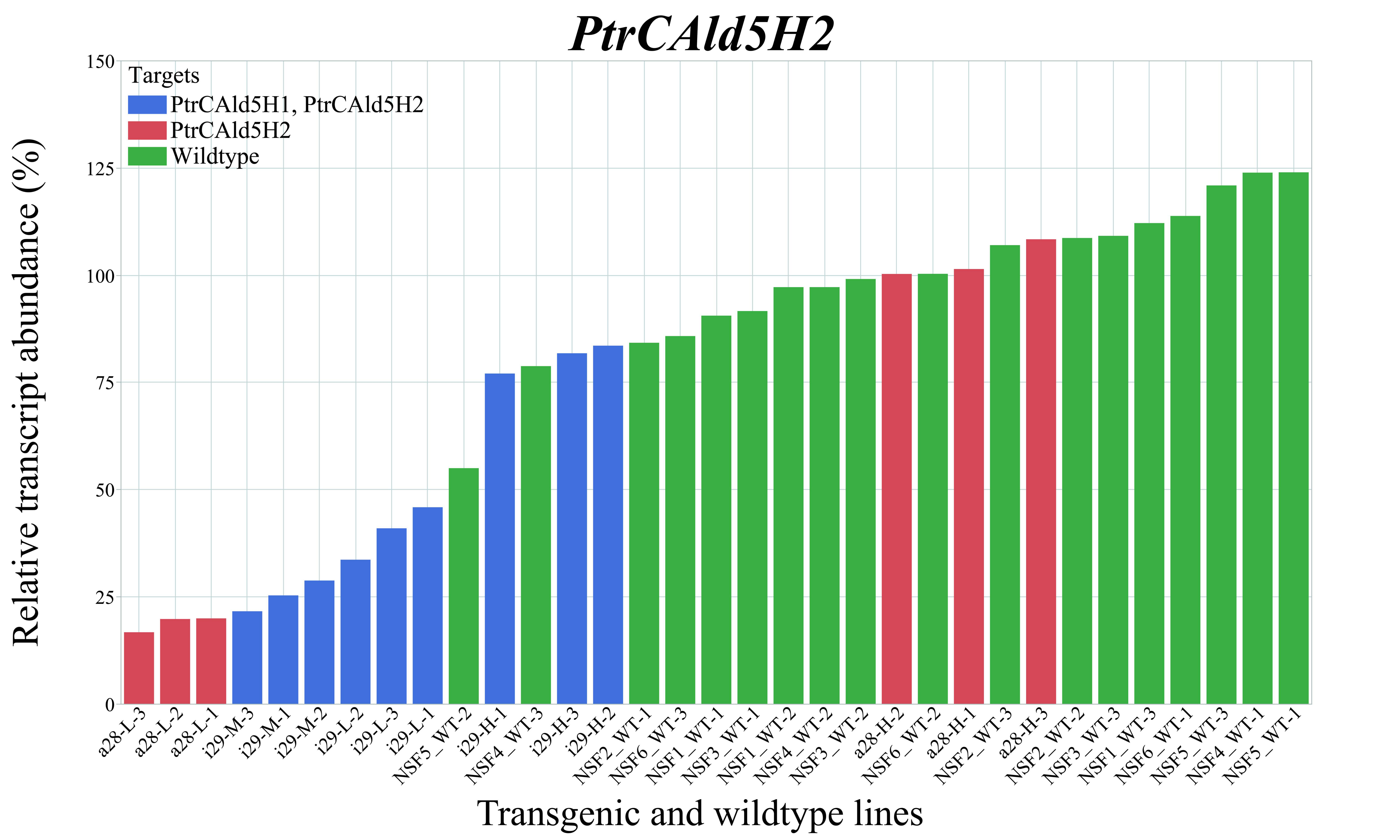

PtrCAld5H2
Relative transcript abundance (%)
150
125
100
75
50
25
0
Targets
PtrCAld5H1, PtrCAld5H2
PtrCAld5H2
Wildtype
a28-L-3
a28-L-2
a28-L-1
i29-M-3
i29-M-1
i29-M-2
i29-L-2
i29-L-3
i29-L-1
NSF5_WT-2
i29-H-1
NSF4_WT-3
i29-H-3
i29-H-2
NSF2_WT-1
NSF6_WT-3
NSF1_WT-1
NSF3_WT-1
NSF1_WT-2
NSF4_WT-2
NSF3_WT-2
a28-H-2
NSF6_WT-2
a28-H-1
NSF2_WT-3
a28-H-3
NSF2_WT-2
NSF3_WT-3
NSF1_WT-3
NSF6_WT-1
NSF5_WT-3
NSF4_WT-1
NSF5_WT-1
Transgenic and wildtype lines

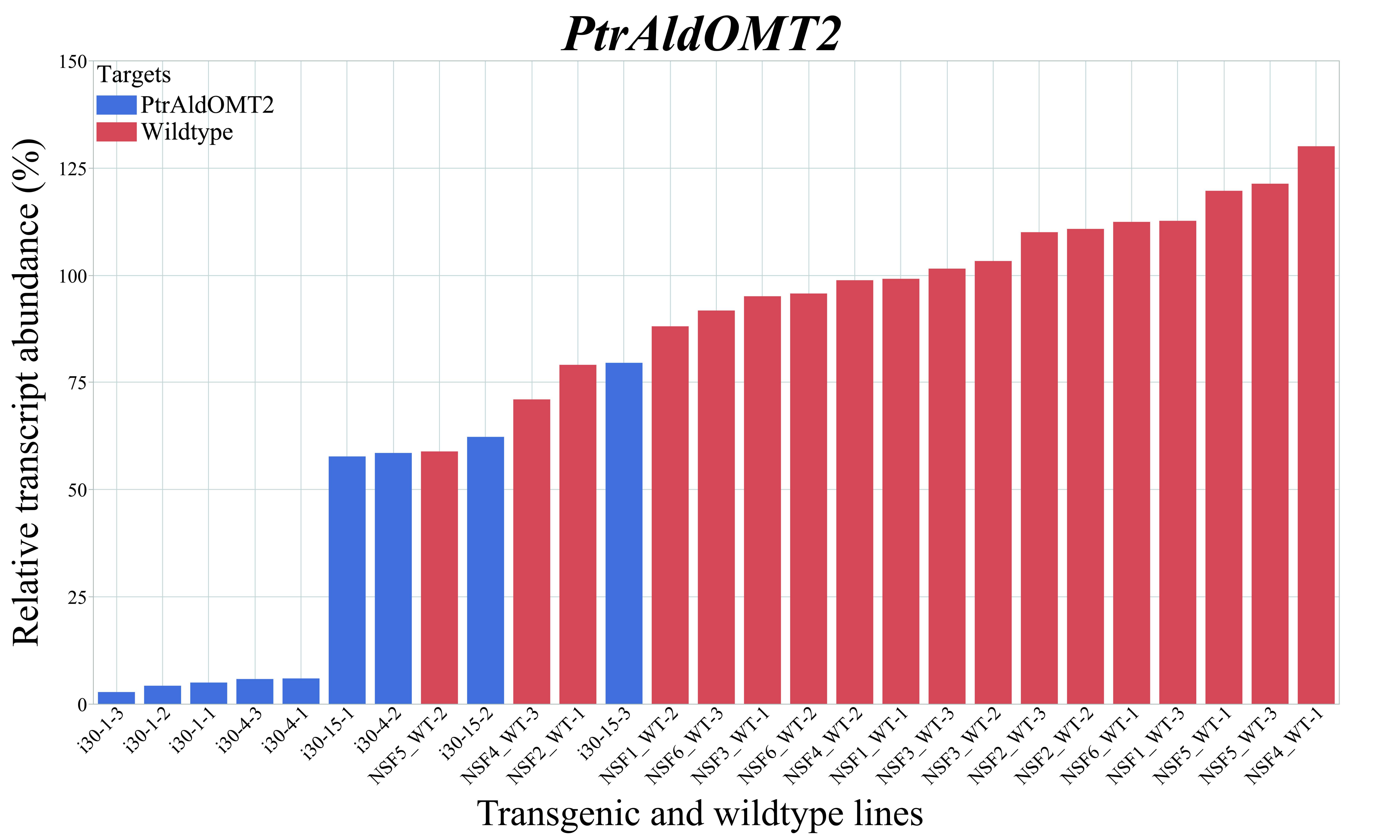
PtrAldOMT2
Targets
PtrAldOMT2
Wildtype
Relative transcript abundance (%)
150
125
100
75
50
25
0
i30-1-3
i30-1-2
i30-1-1
i30-4-3
i30-4-1
i30-15-1
i30-4-2
NSF5_WT-2
i30-15-2
NSF4_WT-3
NSF2_WT-1
i30-15-3
NSF1_WT-2
NSF6_WT-3
NSF3_WT-1
NSF6_WT-2
NSF4_WT-2
NSF1_WT-1
NSF3_WT-3
NSF3_WT-2
NSF2_WT-3
NSF2_WT-2
NSF6_WT-1
NSF1_WT-3
NSF5_WT-1
NSF5_WT-3
NSF4_WT-1
Transgenic and wildtype lines
