# Supplementary material for: Improving wood properties for wood utilization through multi-omics integration in lignin biosynthesis
Source: Nat Commun. 2018 Apr 20;9:1579. doi: 10.1038/s41467-018-03863-z (PMC5910405; doi:10.1038/s41467-018-03863-z)
Supplement: Supplementary file 4 — Supplementary Data 2 [file 41467_2018_3863_MOESM4_ESM.pdf]

Supplementary Data 2 - Equations

Transcript to Protein Equations

Equation #

$$\text{Protein(PtrPAL1)} = 2610 \cdot \text{Transcript}(\textit{PtrPAL1}) \quad (1)$$
$$\text{Protein(PtrPAL2)} = 11940 \cdot \text{Transcript}(\textit{PtrPAL2}) \quad (2)$$
$$\text{Protein(PtrPAL3)} = 2820 \cdot \text{Transcript}(\textit{PtrPAL3}) \quad (3)$$
$$\text{Protein(PtrPAL45)} = 7120 \cdot \text{Transcript}(\textit{PtrPAL45}) \quad (4)$$
$$\text{Protein(PtrC4H1)} = 3100 \cdot \text{Transcript}(\textit{PtrC4H1}) \quad (5)$$
$$\text{Protein(PtrC4H2)} = 2850 \cdot \text{Transcript}(\textit{PtrC4H2}) \quad (6)$$
$$\text{Protein(PtrC3H3)} = 4470 \cdot \text{Transcript}(\textit{PtrC3H3}) \quad (7)$$
$$\text{Protein(Ptr4CL3)} = 43890 \cdot \text{Transcript}(\textit{Ptr4CL3}) \quad (8)$$
$$\text{Protein(Ptr4CL5)} = 14470 \cdot \text{Transcript}(\textit{Ptr4CL5}) \quad (9)$$
$$\text{Protein(PtrHCT1)} = 2920 \cdot \text{Transcript}(\textit{PtrHCT1}) \quad (10)$$
$$\text{Protein(PtrHCT6)} = 17040 \cdot \text{Transcript}(\textit{PtrHCT6}) \quad (11)$$
$$\text{Protein(PtrCCoAOMT1)} = 12720 \cdot \text{Transcript}(\textit{PtrCCoAOMT1}) \quad (12)$$
$$\text{Protein(PtrCCoAOMT2)} = 13440 \cdot \text{Transcript}(\textit{PtrCCoAOMT2}) \quad (13)$$
$$\text{Protein(PtrCCoAOMT3)} = 10820 \cdot \text{Transcript}(\textit{PtrCCoAOMT3}) \quad (14)$$
$$\text{Protein(PtrCCR2)} = 12560 \cdot \text{Transcript}(\textit{PtrCCR2}) \quad (15)$$
$$\text{Protein(PtrCAD1)} = 7180 \cdot \text{Transcript}(\textit{PtrCAD1}) \quad (16)$$
$$\text{Protein(PtrCAD2)} = 13940 \cdot \text{Transcript}(\textit{PtrCAD2}) \quad (17)$$
$$\text{Protein(PtrCAld5H1)} = 6870 \cdot \text{Transcript}(\textit{PtrCAld5H1}) \quad (18)$$
$$\text{Protein(PtrCAld5H2)} = 4080 \cdot \text{Transcript}(\textit{PtrCAld5H2}) \quad (19)$$
$$\text{Protein(PtrAldOMT2)} = 104730 \cdot \text{Transcript}(\textit{PtrAldOMT2}) \quad (20)$$

Metabolic Flux Model Equations

$$V1 = \frac{\mathit{kcat11}\,\mathrm{PtrPAL1}\,y_1}{y_1(1+\frac{y_4}{\mathit{kiu1}}) + \mathit{km11}(1+\frac{y_2}{\mathit{kic11}}+\frac{y_4}{\mathit{kic111}})} + \frac{\mathit{kcat12}\,\mathrm{PtrPAL2}\,y_1}{y_1(1+\frac{y_2}{\mathit{kiu12}}+\frac{y_4}{\mathit{kiu121}}) + \mathit{km12}(1+\frac{y_2}{\mathit{kic12}}+\frac{y_4}{\mathit{kic121}})}$$
$$+ \frac{\mathit{kcat13}\,\mathrm{PtrPAL3}\,y_1}{y_1(1+\frac{y_2}{\mathit{kiu13}}+\frac{y_4}{\mathit{kiu131}}) + \mathit{km13}(1+\frac{y_2}{\mathit{kic13}}+\frac{y_4}{\mathit{kic131}})} + \frac{\mathit{kcat14}\,\mathrm{PtrPAL45}\,y_1}{y_1(1+\frac{y_4}{\mathit{kiu14}}) + \mathit{km14}(1+\frac{y_2}{\mathit{kic14}}+\frac{y_4}{\mathit{kic141}})} \quad (21)$$

$$V2 = \frac{\mathit{kcat21}\,\mathrm{PtrC4H1}\,y_2}{y_2 + \mathit{km21}} + \frac{\mathit{kcat22}\,\mathrm{PtrC4H2}\,y_2}{y_2 + \mathit{km22}} \quad (22)$$

$$V3 = \frac{\mathit{kcat3}\,\mathrm{PtrC3H3}\,y_3}{y_3 + \mathit{km3}} \quad (23)$$

$$V4 = \frac{\mathit{kcat4}\,\mathrm{PtrAldOMT2}\,y_4}{y_4(1+\frac{y_{16}}{\mathit{kiu41}}+\frac{y_{21}}{\mathit{kiu42}}+\frac{y_6}{\mathit{kiu43}}+\frac{y_{18}}{\mathit{kiu44}}+\frac{y_{23}}{\mathit{kiu45}}) + \mathit{km4}(1+\frac{y_{16}}{\mathit{kic41}}+\frac{y_{21}}{\mathit{kic42}}+\frac{y_6}{\mathit{kic43}}+\frac{y_{18}}{\mathit{kic44}}+\frac{y_{23}}{\mathit{kic45}})} \quad (24)$$

$$V5 = \frac{\mathit{kcat51}\,\mathrm{PtrCAld5H1}\,y_5}{y_5(1+\frac{y_{17}}{\mathit{kiu51}}) + \mathit{km51}(1+\frac{y_{17}}{\mathit{kic51}})} + \frac{\mathit{kcat52}\,\mathrm{PtrCAld5H2}\,y_5}{y_5(1+\frac{y_{17}}{\mathit{kiu52}}) + \mathit{km52}(1+\frac{y_{17}}{\mathit{kic52}})} \quad (25)$$

$$V6 = \frac{\mathit{kcat6}\,\mathrm{PtrAldOMT2}\,y_6}{y_6 + \mathit{km6}} \quad (26)$$

$$V7 = \frac{\mathit{kcat71}\,\mathrm{Ptr4CL3}\,y_3}{\mathit{km71}(1+\frac{y_4}{\mathit{k73c1}}+\frac{y_5}{\mathit{k73c2}}+\frac{y_6}{\mathit{k73c3}}+\frac{y_7}{\mathit{k73c4}}+\frac{y_9}{\mathit{k73c5}}+\frac{y_{10}}{\mathit{k73c6}}) + y_3(1+\frac{y_9}{\mathit{k73u1}}+\frac{y_{10}}{\mathit{k73u2}})} \cdots \quad (27)$$
$$\cdots \overline{+\frac{3\mathit{km71}\,\mathrm{Ptr4CL3}^2\mathrm{Ptr4CL5}}{\mathit{K71}^3}(1+\frac{y_4}{\mathit{k75c1}}+\frac{y_5}{\mathit{k75c2}}+\frac{y_6}{\mathit{k75c3}}+\frac{y_7}{\mathit{k75c4}}+\frac{y_9}{\mathit{k75c5}}+\frac{y_{10}}{\mathit{k75c6}}+\frac{y_3}{\mathit{km72}}(1+\frac{y_4}{\mathit{k75u1}}+\frac{y_5}{\mathit{k75u2}}+\frac{y_6}{\mathit{k75u3}}+\frac{y_7}{\mathit{k75u4}}+\frac{y_9}{\mathit{k75u5}}+\frac{y_{10}}{\mathit{k75u6}})}$$
$$+ \frac{\mathit{kcat72}\,\mathrm{Ptr4CL5}\,y_3(1+\gamma\mathit{1}(\frac{\mathrm{Ptr4CL3}}{\mathit{K72}})^3)}{\mathit{km72}(1+\frac{y_4}{\mathit{k75c1}}+\frac{y_5}{\mathit{k75c2}}+\frac{y_6}{\mathit{k75c3}}+\frac{y_7}{\mathit{k75c4}}+\frac{y_9}{\mathit{k75c5}}+\frac{y_{10}}{\mathit{k75c6}}) + y_3(1+\frac{y_4}{\mathit{k75u1}}+\frac{y_5}{\mathit{k75u2}}+\frac{y_6}{\mathit{k75u3}}+\frac{y_7}{\mathit{k75u4}}+\frac{y_9}{\mathit{k75u5}}+\frac{y_{10}}{\mathit{k75u6}})(1+\frac{\mathrm{Ptr4CL3}}{\mathit{K72}})^3}$$

$$V8 = \frac{\mathit{kcat81}\,\mathrm{Ptr4Cl3}\,y_4}{\mathit{km81}(1+\frac{y_3}{\mathit{k83c1}}+\frac{y_5}{\mathit{k83c2}}+\frac{y_6}{\mathit{k83c3}}+\frac{y_9}{\mathit{k83c4}}) + y_4(1+\frac{y_9}{\mathit{k83u1}})} \cdots \quad (28)$$
$$\cdots \overline{+\frac{3\mathit{km81}\,\mathrm{Ptr4CL3}^2\mathrm{Ptr4CL5}}{\mathit{K81}^3}(1+\frac{y_3}{\mathit{k85c1}}+\frac{y_5}{\mathit{k85c2}}+\frac{y_6}{\mathit{k85c3}}+\frac{y_9}{\mathit{k85c4}}+\frac{y_4}{\mathit{km82}}(1+\frac{y_3}{\mathit{k85u1}}+\frac{y_5}{\mathit{k85u2}}+\frac{y_6}{\mathit{k85u3}}+\frac{y_7}{\mathit{k85u4}}+\frac{y_9}{\mathit{k85u5}}+\frac{y_4}{\mathit{k8is}}))}$$
$$+ \frac{\mathit{kcat82}\,\mathrm{Ptr4CL5}\,y_4(1+\gamma\mathit{2}(\frac{\mathrm{Ptr4CL3}}{\mathit{K82}})^3)}{\mathit{km82}(1+\frac{y_3}{\mathit{k85c1}}+\frac{y_5}{\mathit{k85c2}}+\frac{y_6}{\mathit{k85c3}}+\frac{y_9}{\mathit{k85c4}}) + y_4(1+\frac{y_3}{\mathit{k85u1}}+\frac{y_5}{\mathit{k85u2}}+\frac{y_6}{\mathit{k85u3}}+\frac{y_7}{\mathit{k85u4}}+\frac{y_9}{\mathit{k85u5}}+\frac{y_4}{\mathit{k8is}})(1+(\frac{\mathrm{Ptr4CL3}}{\mathit{K82}})^3)}$$

$$V9 = \frac{kcat91 \mathrm{Ptr4CL3} y_5}{km91(1 + \frac{y_3}{k93c1} + \frac{y_4}{k93c2} + \frac{y_6}{k93c3} + \frac{y_7}{k93c4}) + y_5} \tag{29}$$

$$+ \frac{kcat92 \mathrm{Ptr4CL5} y_5}{km92(1 + \frac{y_3}{k95c1} + \frac{y_4}{k95c2} + \frac{y_6}{k95c3} + \frac{y_7}{k95c4}) + y_5(1 + \frac{y_4}{k95u1} + \frac{y_6}{k95u2} + \frac{y_7}{k95u3})}$$

$$V10 = \frac{kcat101 \mathrm{Ptr4CL3} y_6}{km101 + y_6} + \frac{kcat102 \mathrm{Ptr4CL5} y_6}{km102 + y_6} \tag{30}$$

$$V11 = \frac{kcat111 \mathrm{Ptr4CL5} y_7}{km111 + y_7} \tag{31}$$

$$V12 = \frac{kcat121 \mathrm{PtrHCT1} y_8}{y_8 + km121} + \frac{kcat122 \mathrm{PtrHCT6} y_8}{y_8 + km122} \tag{32}$$

$$V12R = \frac{kcat121R \mathrm{PtrHCT1} y_9}{y_9 + km121R} + \frac{kcat122R \mathrm{PtrHCT6} y_9}{y_9 + km122R} \tag{33}$$

$$V13 = \frac{kcat131 \mathrm{PtrC3H3} y_9}{y_9 + km131} \tag{34}$$

$$V14 = \frac{kcat141 \mathrm{PtrHCT1} y_{10}}{y_{10} + km141} + \frac{kcat142 \mathrm{PtrHCT6} y_{10}}{y_{10} + km142} \tag{35}$$

$$V14R = \frac{kcat141R \mathrm{PtrHCT1} y_{11}}{y_{11} + km141R} + \frac{kcat142R \mathrm{PtrHCT6} y_{11}}{y_{11} + km142R} \tag{36}$$

$$V15 = \frac{kcat151 \mathrm{PtrCCoAOMT1} y_{11}}{y_{11} + km151} + \frac{kcat152 \mathrm{PtrCCoAOMT2} y_{11}}{y_{11} + km152} + \frac{kcat153 \mathrm{PtrCCoAOMT3} y_{11}}{y_{11} + km153} \tag{37}$$

$$V16 = 0 \tag{38}$$

$$V17 = \frac{kcat181 \mathrm{PtrCCR2} y_8}{y_8 + km181} \tag{39}$$

$$V18 = \frac{kcat191 \mathrm{PtrCCR2} y_{11}}{y_{11} + km191} \tag{40}$$

$$V19 = \frac{kcat201 \mathrm{PtrCCR2} y_{12}}{y_{12} + km201} \tag{41}$$

$$V20 = 0 \tag{42}$$

$$V21 = 0 \tag{43}$$

$$V22 = \frac{kcat241\,\mathrm{PtrAldOMT2}\,y_{16}}{y_{16}(1 + \frac{y_{18}}{kiu241} + \frac{y_{16}}{kis241}) + km241(1 + \frac{y_4}{kic241} + \frac{y_6}{kic242} + \frac{y_{18}}{kic243} + \frac{y_{23}}{kic244})} \tag{44}$$

$$V23 = \frac{kcat251\,\mathrm{PtrCAld5H1}\,y_{17}}{y_{17} + km251} + \frac{kcat252\,\mathrm{PtrCAld5H2}\,y_{17}}{y_{17} + km252} \tag{45}$$

$$V24 = \frac{kcat261\,\mathrm{PtrAldOMT2}\,y_{18}}{y_{18}(1 + \frac{y_{21}}{kiu261} + \frac{y_{18}}{kis261}) + km261(1 + \frac{y_{16}}{kic261} + \frac{y_{21}}{kic262} + \frac{y_{23}}{kic263})} \tag{46}$$

$$V25 = \frac{kcat271\,\mathrm{PtrCAD1}\,y_{15}}{y_{15} + km271} \tag{47}$$

$$V26 = 0 \tag{48}$$

$$V27 = \frac{kcat291\,\mathrm{PtrCAD1}\,y_{17}}{y_{17}(1 + \frac{y_{19}}{kiu291}) + km291(1 + \frac{y_{19}}{kic291})} + \frac{kcat292\,\mathrm{PtrCAD2}\,y_{17}}{y_{17} + km292} \tag{49}$$

$$V28 = \frac{kcat281\,\mathrm{PtrCAD1}\,y_{18}}{y_{18} + km281} \tag{50}$$

$$V29 = \frac{kcat311\,\mathrm{PtrCAD1}\,y_{19}}{y_{19}(1 + \frac{y_{17}}{kiu311}) + km311(1 + \frac{y_{17}}{kic311})} + \frac{kcat312\,\mathrm{PtrCAD2}\,y_{19}}{y_{19} + km312} \tag{51}$$

$$V30 = \frac{kcat331\,\mathrm{PtrAldOMT2}\,y_{21}}{y_{21}(1 + \frac{y_{16}}{kiu331} + \frac{y_{18}}{kiu332} + \frac{y_{23}}{kiu333} + \frac{y_{21}}{kis331}) + km331(1 + \frac{y_{16}}{kic331} + \frac{y_{18}}{kic332})} \tag{52}$$

$$V31 = \frac{kcat341\,\mathrm{PtrCAld5H1}\,y_{22}}{y_{22}(1 + \frac{y_{17}}{kiu341}) + km341(1 + \frac{y_{17}}{kic341})} + \frac{kcat342\,\mathrm{PtrCAld5H2}\,y_{22}}{y_{22} + km342(1 + \frac{y_{17}}{kic342})} \tag{53}$$

$$V32 = \frac{kcat351\,\mathrm{PtrAldOMT2}\,y_{23}}{y_{23}(1 + \frac{y_{23}}{kis351}) + km351} \tag{54}$$

$$V33 = V25 \tag{55}$$

$$V34 = V27 + V30 - V31 \tag{56}$$

$$V35 = V29 + V32 \tag{57}$$

$$V36 = V19 + V22 - V23 - V27 \tag{58}$$

$$V37 = V21 + V24 - V29 \tag{59}$$

Multiple Linear Regression Equations

$$\text{Lignin content} = 11.883484066567 + 0.0615160893227361 \cdot y_2 + 0.430775696181125 \cdot y_3 + 12.9331022489554 \cdot y_4 - 1510958.74672428 \cdot y_6 + 0.0245385806501869 \cdot y_{10} - 0.155450728780881 \cdot y_{12} + 98.4523529683073 \cdot y_{15} - 171.334283931017 \cdot V_9 - 26163166.4690442 \cdot V_{10} + 816571.202252856 \cdot V_{11} - 276.483774555191 \cdot V_{12R} + 17.361214501387 \cdot V_{14} - 127.913448294397 \cdot V_{22} - 788.338526656292 \cdot V_{28} - 553.474602033791 \cdot V_{33} \tag{60}$$

$$\text{S/G ratio} = 3.1570276042466 - 0.0274456587679616 \cdot y_2 + 0.126857492675907 \cdot y_3 - 0.012565495544623 \cdot y_{10} + 45.9299050501466 \cdot V_{12R} - 4.82667873491386 \cdot V_{19} + 18.7284099558843 \cdot V_{24} + 10534.8599066237 \cdot V_{28} + 170.541719415153 \cdot V_{33} - 3.02655953685301 \cdot V_{35} - 7.55232058799794 \cdot V_{37} \tag{61}$$

$$\text{S-subunits} = 66.362453585523 - 0.142414154817709 \cdot y_2 + 0.982676235224877 \cdot y_3 - 122.252554678186 \cdot y_4 + 586.345817026914 \cdot y_{15} - 59.4354897874603 \cdot V_8 + 1204.73818121718 \cdot V_9 - 642.451768901524 \cdot V_{12R} + 4064751.98736214 \cdot V_{24} + 4064647.60234122 \cdot V_{27} + 4176236.00315314 \cdot V_{28} - 825.140551408177 \cdot V_{33} - 4064665.84656898 \cdot V_{34} - 4064642.90336438 \cdot V_{35} - 4064657.46203729 \cdot V_{37} \tag{62}$$

$$\text{G-subunits} = 24.9333908693591 - 172.695869367026 \cdot V_9 - 106.045202018152 \cdot V_{12R} + 61.6897059860666 \cdot V_{19} - 202.741777492097 \cdot V_{24} - 124312.502003196 \cdot V_{28} \tag{63}$$

$$\text{H-subunits} = 5.90091370623289 + 0.19483368749435 \cdot y_2 + 175.584250588986 \cdot y_4 - 89.7624828206843 \cdot y_5 - 0.0360622385554641 \cdot y_{10} - 1274365864.26485 \cdot V_{10} + 20167378.4235956 \cdot V_{11} + 501.603326960271 \cdot V_{12R} - 37.2627044987101 \cdot V_{14} - 3274260.66695319 \cdot V_{27} - 3247529.33792158 \cdot V_{28} - 3274217.88943963 \cdot V_{29} + 942.543781532183 \cdot V_{33} + 3274260.34700786 \cdot V_{34} + 3274248.40755471 \cdot V_{35} \tag{64}$$

$$p\text{-Hydroxybenzoic acid} = 5.45079151188417 - 43.9088121523739 \cdot y_5 + 0.00676991555397219 \cdot y_{10} + 0.070548037623962 \cdot y_{12} + 208.769681557457 \cdot y_{15} + 917.24012258393 \cdot V_9 - 506818086.727017 \cdot V_{10} + 7377307.63148245 \cdot V_{11} - 7.67803565021953 \cdot V_{19} + 453.262746723218 \cdot V22 + 5105.24360947262 \cdot V_{28} \tag{65}$$

$$\text{C:L ratio} = 5.61581360130528 - 0.0152293286030458 \cdot y_2 - 0.151047984245847 \cdot y_3 - 4.14292404177484 \cdot y_4 + 508228.060172331 \cdot y_6 - 0.00472230646148822 \cdot y_{10} + 0.036277592219895 \cdot y_{12} - 25.8825858983041 \cdot y_{15} + 55.5280609499425 \cdot V_9 + 10461990.2457532 \cdot V_{10} - 280841.52481246 \cdot V_{11} + 104.699410014405 \cdot V_{12R} - 5.14000773443813 \cdot V_{14} + 34.2947351255568 \cdot V_{22} + 186.65811319424 \cdot V_{33} \tag{66}$$

$$\text{Aldehydes} = -0.0421120117513878 - 0.0444778017427485 \cdot y_{10} + 1377.773182781 \cdot y_{15} + 45359.8117456874 \cdot V_{28} + 5.52316297586782 \cdot V_{34} + 64.0651007793339 \cdot V_{36} - 167.937473057631 \cdot V_{37} \tag{67}$$

$$\beta\text{-O-4 linkages} = 92.3423375623908 - 0.0332063596474041 \cdot y_2 - 16.130431028688 \cdot y_5 + 0.0650829184618269 \cdot y_{12}$$
$$+ 769.694359207219 \cdot y_{15} - 437544.322388889 \cdot y_{16} + 417.737372522682 \cdot V_8 + 791.566150496345 \cdot V_9$$
$$- 126.400277598401 \cdot V_{12R} + 423.921152751101 \cdot V_{14} - 436.202965205955 \cdot V_{19} + 41831.1849387552 \cdot V_{28}$$
$$+ 10.6825704700725 \cdot V_{36} \quad (68)$$

$$\beta\text{-5 linkages} = 3.39164364140183 - 0.169452664632931 \cdot y_3 + 20.9772415816131 \cdot y_4 - 240.923514745077 \cdot y_{15}$$
$$+ 10.9609692960252 \cdot V_8 - 205.631782160621 \cdot V_9 + 121.47242963438 \cdot V_{12R} - 21.3218773686486 \cdot V_{24}$$
$$- 26121.148561243 \cdot V_{28} + 247.037419382746 \cdot V_{33} + 5.45245403568805 \cdot V_{34} \quad (69)$$

$$\beta\text{-}\beta\text{ linkages} = 4.30703483808592 + 0.0366962685358232 \cdot y_2 + 0.195650357937761 \cdot y_3 + 5863652.23866847 \cdot y_6$$
$$- 0.0817543549617492 \cdot y_{12} - 306.778739733446 \cdot y_{15} + 160314.473992819 \cdot y_{16} - 104156839.255285 \cdot V_{10}$$
$$+ 2.5180532932565 \cdot V_{14} - 534.273543552229 \cdot V_{22} + 51.6172255545255 \cdot V_{24} - 38.4809623268958 \cdot V_{29}$$
$$- 2.52922662637061 \cdot V_{34} - 16.0238753299495 \cdot V_{36} \quad (70)$$

$$\beta\text{-1 linkages} = 1.39579152213555 - 0.0163005734222925 \cdot y_2 - 26.8426477691316 \cdot V_{12R} + 1.52200331093853 \cdot V_{27}$$
$$+ 0.629787850157707 \cdot V_{35} \quad (71)$$

$$\text{End groups} = 5.6140947921364 - 116.900826282251 \cdot y_{15} + 31.1374390125443 \cdot V_{12R} + 6.40796107990149 \cdot V_{27}$$
$$- 21361.5277590907 \cdot V_{28} - 14.4693809339611 \cdot V_{29} + 102.233813423251 \cdot V_{33} \quad (72)$$

$$\text{Height} = 55.217222366891 - 147.76909184373 \cdot y_4 + 39.2538187061372 \cdot y_5 - 0.156568016514816 \cdot y_{10}$$
$$- 1.34492375694259 \cdot y_{12} + 3647.76497632402 \cdot y_{15} - 284.808929027467 \cdot V_8 + 2324755954.58088 \cdot V_{10}$$
$$- 45170718.3124317 \cdot V_{11} + 309.449697944976 \cdot V_{19} - 286.710136340745 \cdot V_{24} - 9267.46094109885 \cdot V_{33}$$
$$+ 45.1704086286282 \cdot V_{35} \quad (73)$$

$$\text{Diameter} = 0.193737734688007 + 247115.678280773 \cdot y_6 - 0.00067108723691074 \cdot y_{10} - 0.003245375306696 \cdot y_{12}$$
$$+ 6.93149620764165 \cdot y_{15} - 17.42482861001 \cdot V_8 - 18.5087320019907 \cdot V_9 - 6654290.80792817 \cdot V_{10}$$
$$+ 1.21059433882589 \cdot V_{12R} - 16.9923927551725 \cdot V_{14} + 17.7832320861399 \cdot V_{19} + 53.8939287864137 \cdot V_{22}$$
$$- 1.10775234073647 \cdot V_{24} - 34.2830997133729 \cdot V_{33} + 0.155555624014883 \cdot V_{35} \quad (74)$$

$$\text{Stem volume} = 45.2101603589951 + 83385926.9183007 \cdot y_6 - 0.146880676634673 \cdot y_{10} - 0.549318751362996 \cdot y_{12}$$
$$+ 2234.86006839747 \cdot y_{15} - 110709.876983525 \cdot y_{16} - 260.371210766318 \cdot V_9 - 2273254241.74673 \cdot V_{10}$$
$$- 902.719915430231 \cdot V_{22} + 116.719097160165 \cdot V_{27} - 83.3319333598196 \cdot V_{34} \quad (75)$$

$$\begin{aligned}\text{Glucose} &= 49.5207670644154 + 409733.761012481 \cdot y_6 - 0.0197838367663708 \cdot y_{10} - 497.38948658234 \cdot y_{15} \\ &+ 73.4799365261709 \cdot V_{12R} + 8.67468242950127 \cdot V_{14} + 90.3837231533935 \cdot V_{22} - 15.8002308535066 \cdot V_{27} \\ &+ 1196.52885488995 \cdot V_{33} + 4.20137980367653 \cdot V_{34} - 60.6138792290271 \cdot V_{37}\end{aligned} \tag{76}$$

$$\begin{aligned}\text{Xylose} &= 13.2340544358712 + 0.0450032261670726 \cdot y_2 + 0.417033084141651 \cdot y_5 - 7.91759240819401 \cdot V_8 \\ &- 107355.940257537 \cdot V_{11} + 50.0003342627561 \cdot V_{12R} - 24.4596771428879 \cdot V_{24} + 613.491204266368 \cdot V_{28} \\ &+ 40.1582998707743 \cdot V_{29} + 318.389292925185 \cdot V_{33} + 2.3031034821691 \cdot V_{34} + 12.0741715290212 \cdot V_{36}\end{aligned} \tag{77}$$

$$\begin{aligned}\text{Total carbohydrates} &= 64.8061680750841 + 0.0851207782825051 \cdot y_2 + 3.32679232194521 \cdot y_4 - 415.878839205454 \cdot y_{15} \\ &+ 86.3980840880056 \cdot V_{12R} + 18.0952265711422 \cdot V_{14} - 16.2961533691979 \cdot V_{27} + 1106.92971751506 \cdot V_{33} \\ &- 66.9280131510257 \cdot V_{37}\end{aligned} \tag{78}$$

$$\begin{aligned}\text{Relative density} &= 0.225001190246316 - 3.16296419918166 \cdot y_{15} - 1.85156619642189 \cdot V_{12R} + 0.237696041232694 \cdot V_{14} \\ &+ 39.1706788655453 \cdot V_{22} - 0.291813398311222 \cdot V_{24} - 0.235224539397824 \cdot V_{34}\end{aligned} \tag{79}$$

$$\begin{aligned}\text{MOE} &= 2053.90452909974 + 63.0317047320743 \cdot y_3 - 90705.3328067901 \cdot y_{15} - 3516.7773221497 \cdot V_8 \\ &- 12667.2770557616 \cdot V_9 - 59013.3315301986 \cdot V_{12R} + 12814.6784468892 \cdot V_{24} + 5124969.65333501 \cdot V_{28} \\ &+ 1350.00153464048 \cdot V_{34}\end{aligned} \tag{80}$$

$$\begin{aligned}\text{Glucose: unpretreated} &= 59.9039107111165 - 0.176436032690008 \cdot y_2 - 1.26535870794446 \cdot y_3 - 27.770864466219 \cdot y_4 \\ &+ 3821691.11581983 \cdot y_6 - 0.104456111874291 \cdot y_{10} + 0.904474251281786 \cdot y_{12} - 538.883329452768 \cdot y_{15} \\ &+ 448.501848362333 \cdot V_9 + 149017444.485561 \cdot V_{10} - 2829213.6608253 \cdot V_{11} + 1116.35923039934 \cdot V_{12R} \\ &- 89.0537982589155 \cdot V_{14} + 531.122088693195 \cdot V_{22} + 9180.29595852483 \cdot V_{28} + 1729.27144714913 \cdot V_{33} \\ &+ 23.8734104102483 \cdot V_{34}\end{aligned} \tag{81}$$

$$\begin{aligned}\text{Xylose: unpretreated} &= 29.9052382167019 - 0.138940920026091 \cdot y_2 - 1.24258864263844 \cdot y_3 - 20.2631932248452 \cdot y_4 \\ &+ 3624704.15929145 \cdot y_6 - 0.103905383089876 \cdot y_{10} + 1.04462008500161 \cdot y_{12} + 112.43165099887 \cdot V_8 \\ &+ 571.04145280866 \cdot V_9 - 2266755.33417554 \cdot V_{11} + 1236.49901273553 \cdot V_{12R} - 72.9260368882047 \cdot V_{19} \\ &+ 14466.40674879 \cdot V_{28} + 2660.31413772912 \cdot V_{33} - 32.8688145703174 \cdot V_{35} - 78.725444825655 \cdot V_{37}\end{aligned} \tag{82}$$

$$\begin{aligned}\text{Glucose: pretreated} &= 88.1676753891115 - 0.32885184583179 \cdot y_2 - 1.40231640140802 \cdot y_3 - 0.140400926894907 \cdot y_{10} \\ &+ 0.725270052240393 \cdot y_{12} + 96.2523005967218 \cdot V_9 - 378781.654373939 \cdot V_{11} + 937.754516698647 \cdot V_{12R} \\ &- 90.1805569095505 \cdot V_{14} + 617.542613061244 \cdot V_{22} + 46.6166763751242 \cdot V_{24} + 6522.80009121018 \cdot V_{28} \\ &+ 2620.23551779516 \cdot V_{33}\end{aligned} \tag{83}$$

$$
\begin{aligned}
\text{Xylose: pretreated} = {} & 92.9696249391059 - 0.339135686063301 \cdot y_2 - 1.74754890214763 \cdot y_3 - 41.4787525339963 \cdot y_4 \\
& + 5082182.16822917 \cdot y_6 - 0.121310557513274 \cdot y_{10} + 0.797988499615414 \cdot y_{12} + 1330.15230248297 \cdot y_{15} \\
& + 3340.82085146819 \cdot V_8 + 3897.36685785626 \cdot V_9 + 131087450.062186 \cdot V_{10} - 2986650.30803981 \cdot V_{11} \\
& + 863.634642837035 \cdot V_{12R} + 3205.30235991669 \cdot V_{14} + 6468.52882403263 \cdot V_{19} - 9898.55741248951 \cdot V_{24} \\
& - 9758.85708349056 \cdot V_{27} + 3642.64363622496 \cdot V_{33} - 45.5972867250081 \cdot V_{34} - 9772.58980472387 \cdot V_{36}
\end{aligned} \tag{84}
$$
