# Supplementary figures and images for: Improving wood properties for wood utilization through multi-omics integration in lignin biosynthesis
Source: Nat Commun. 2018 Apr 20;9:1579. doi: 10.1038/s41467-018-03863-z (PMC5910405; doi:10.1038/s41467-018-03863-z)

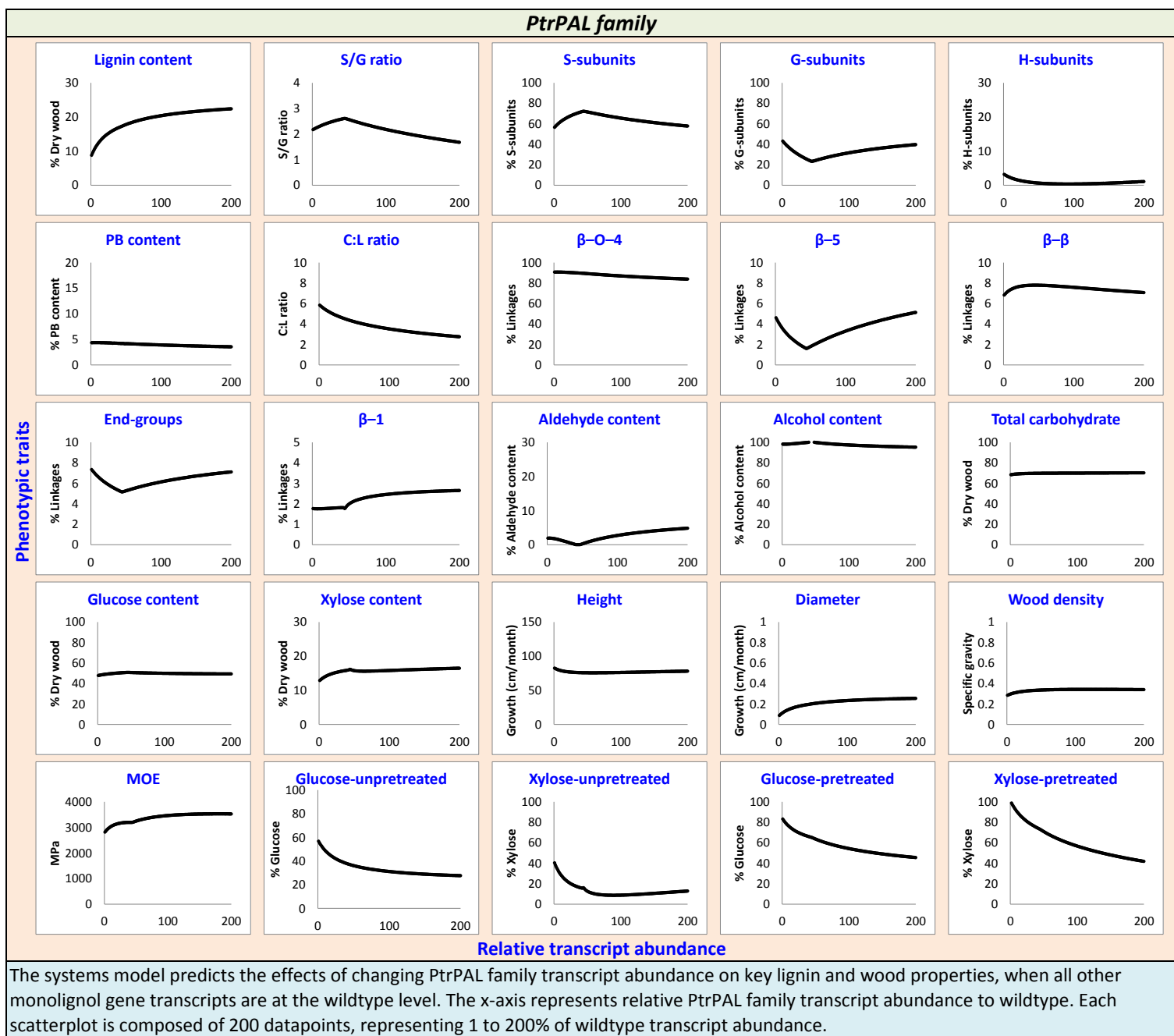

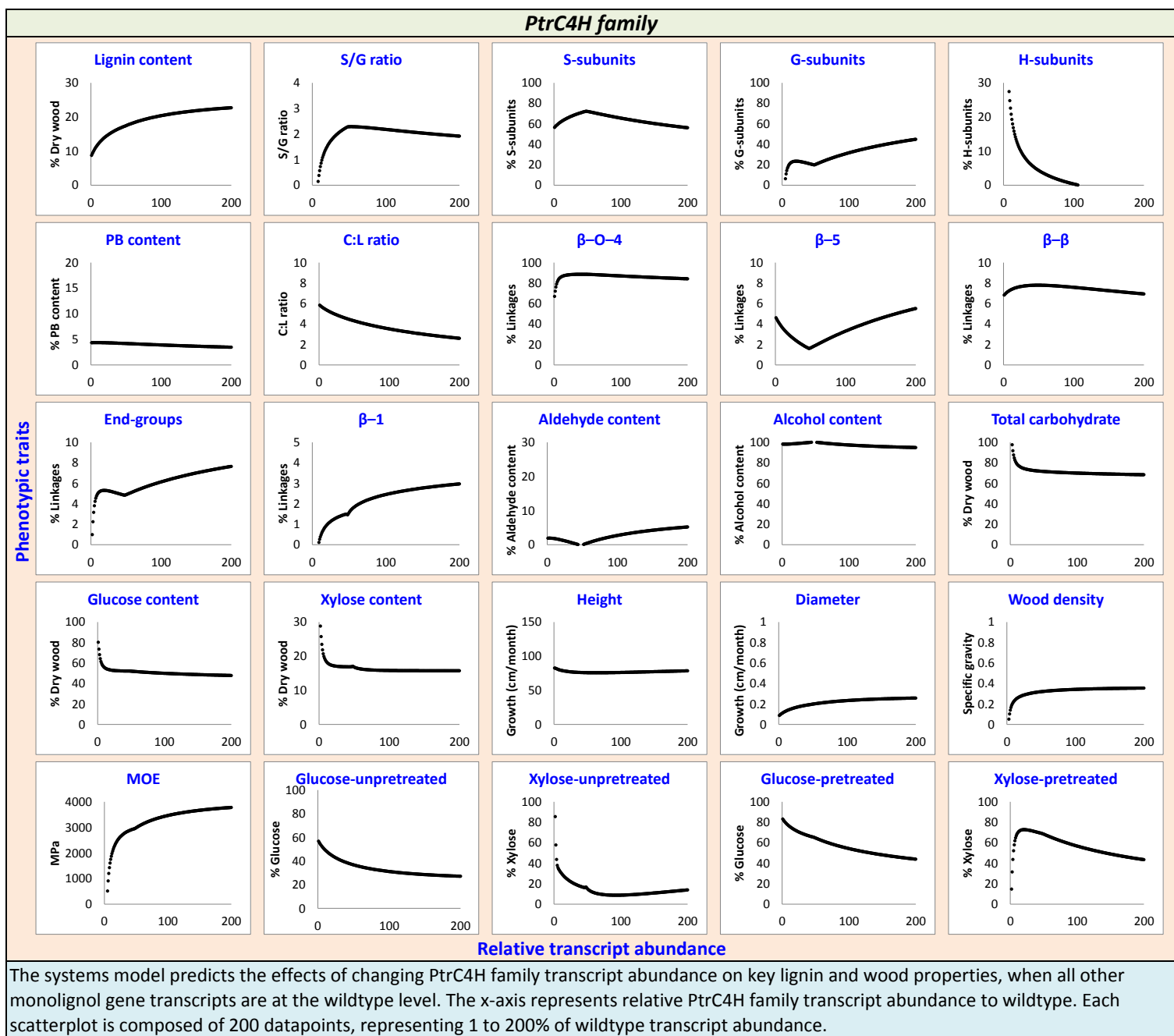

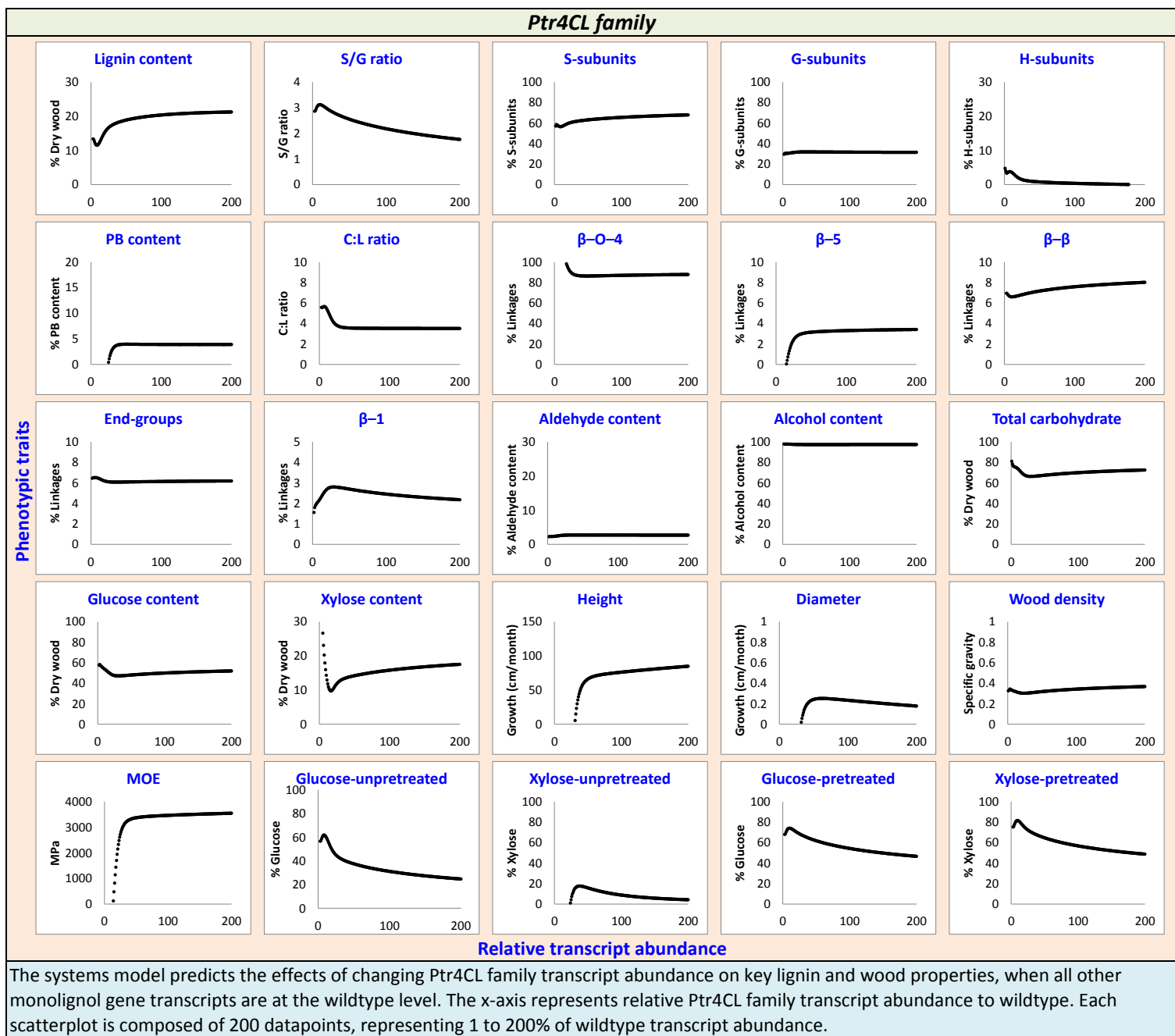

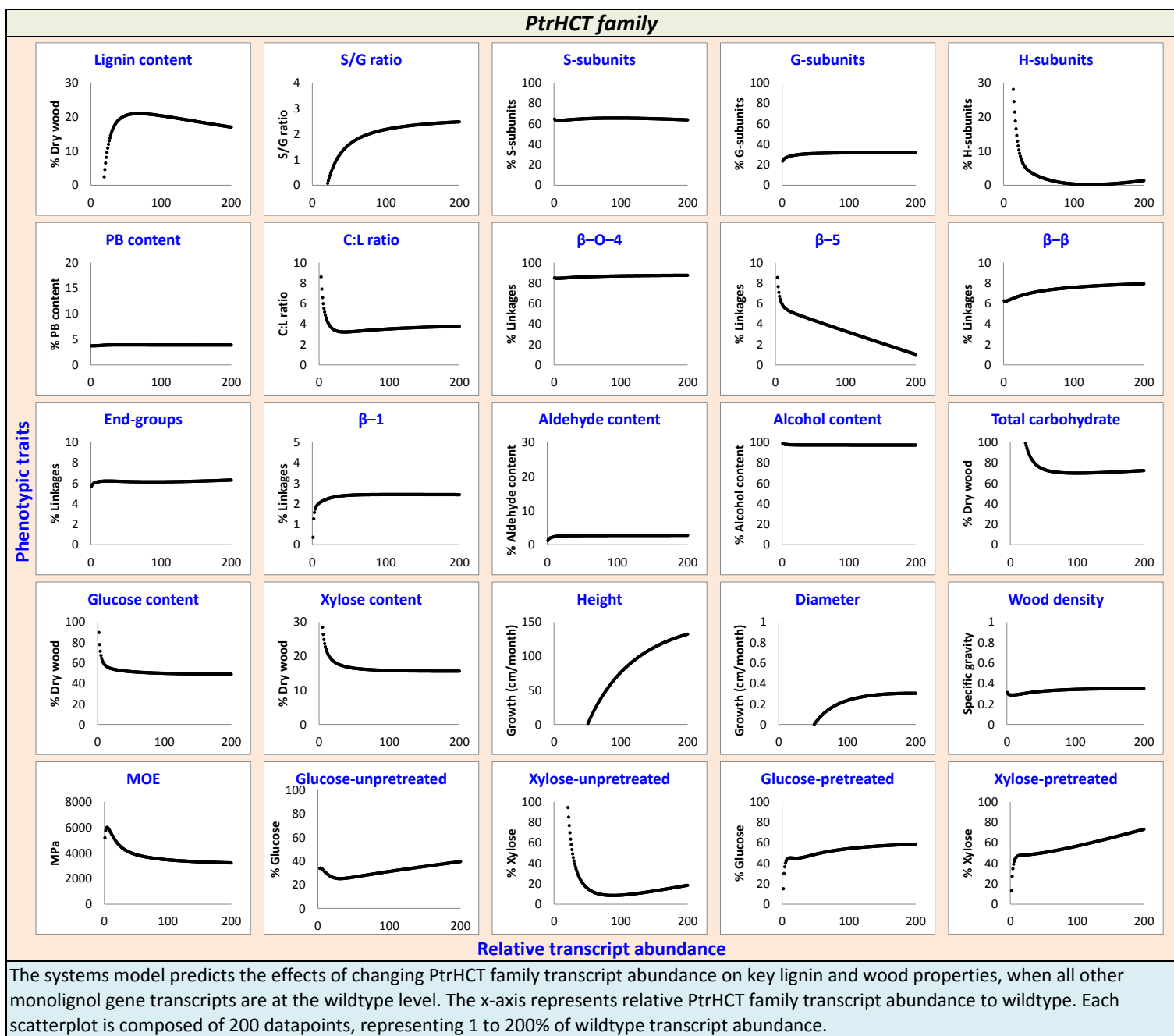

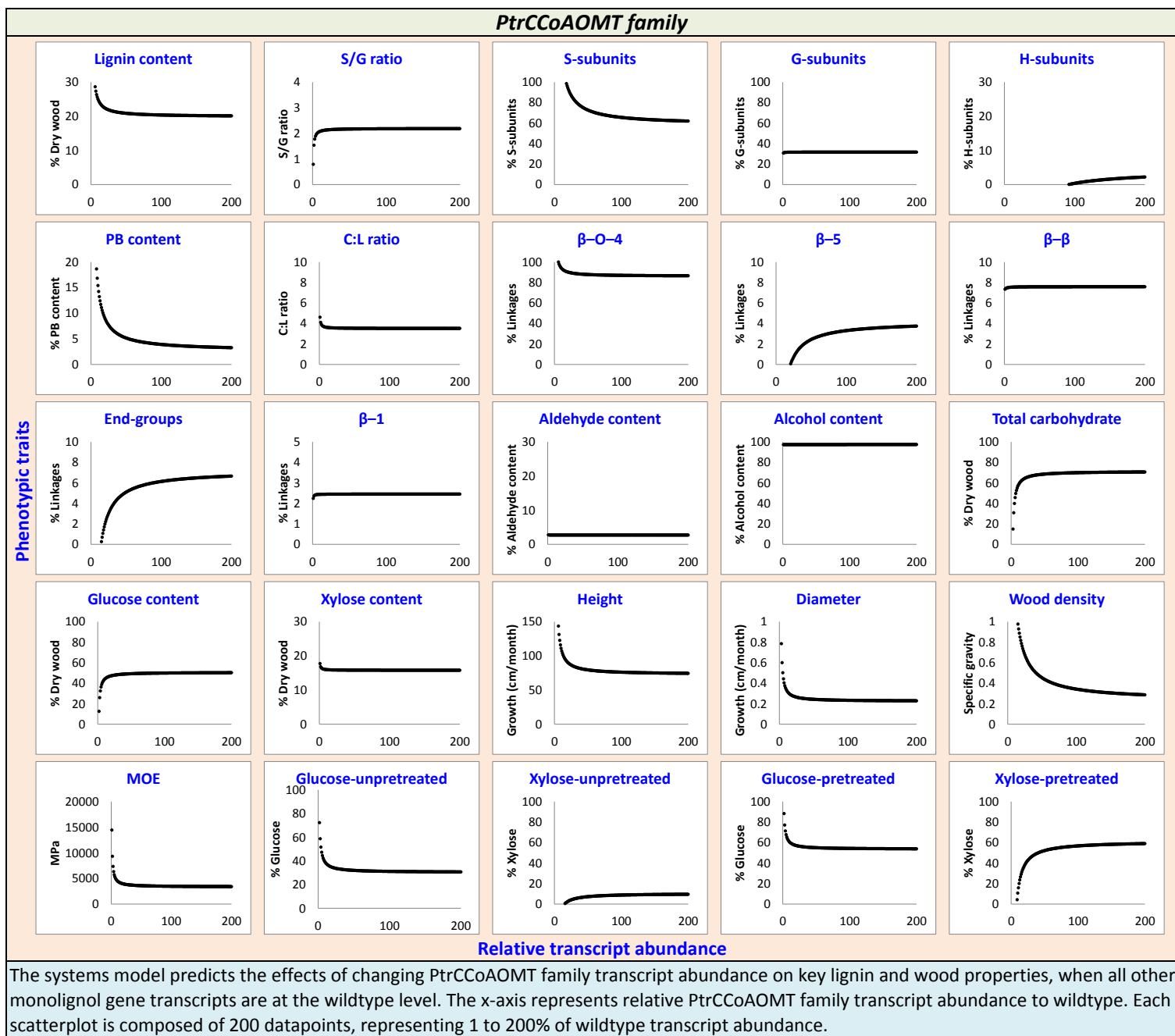

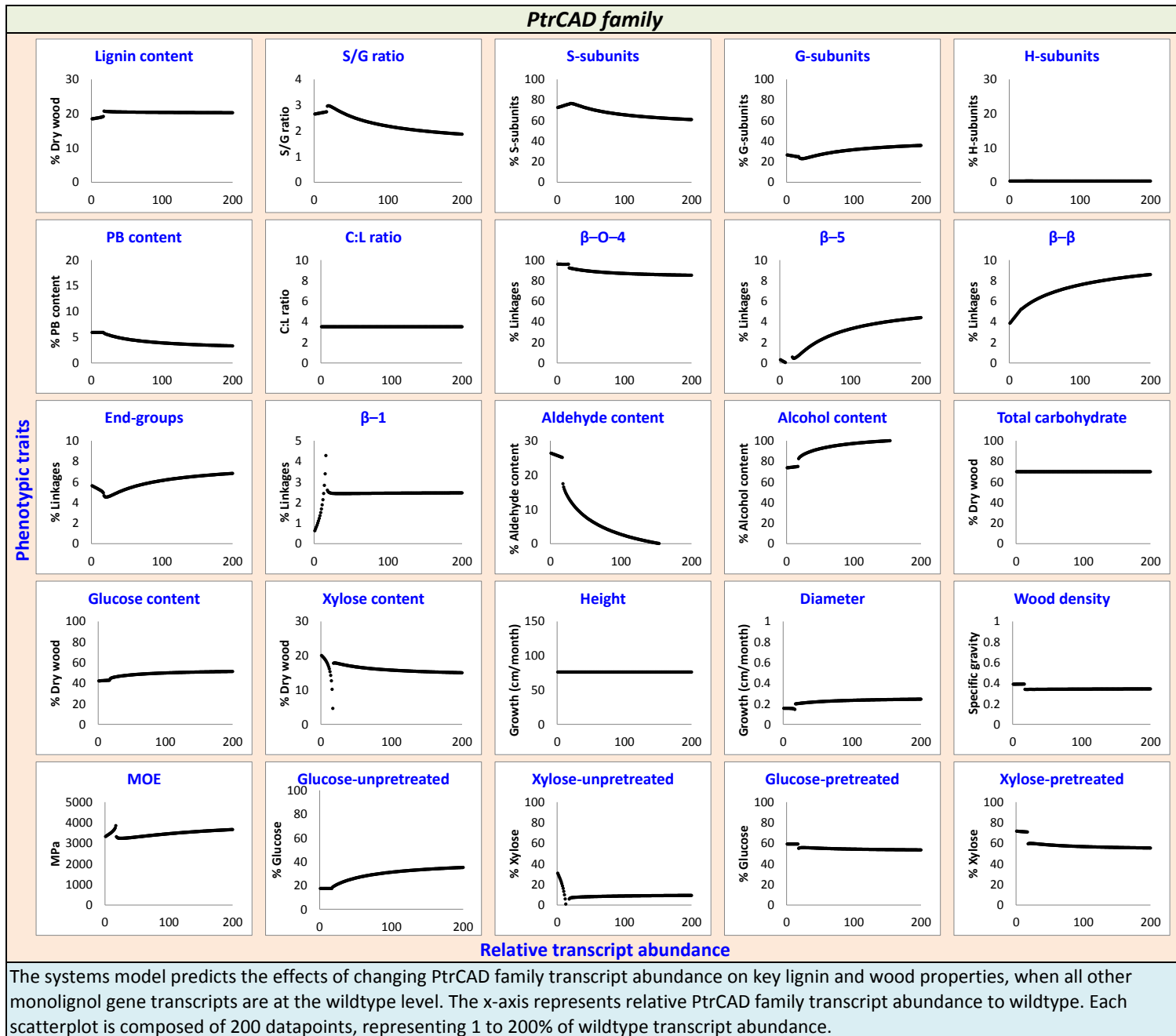

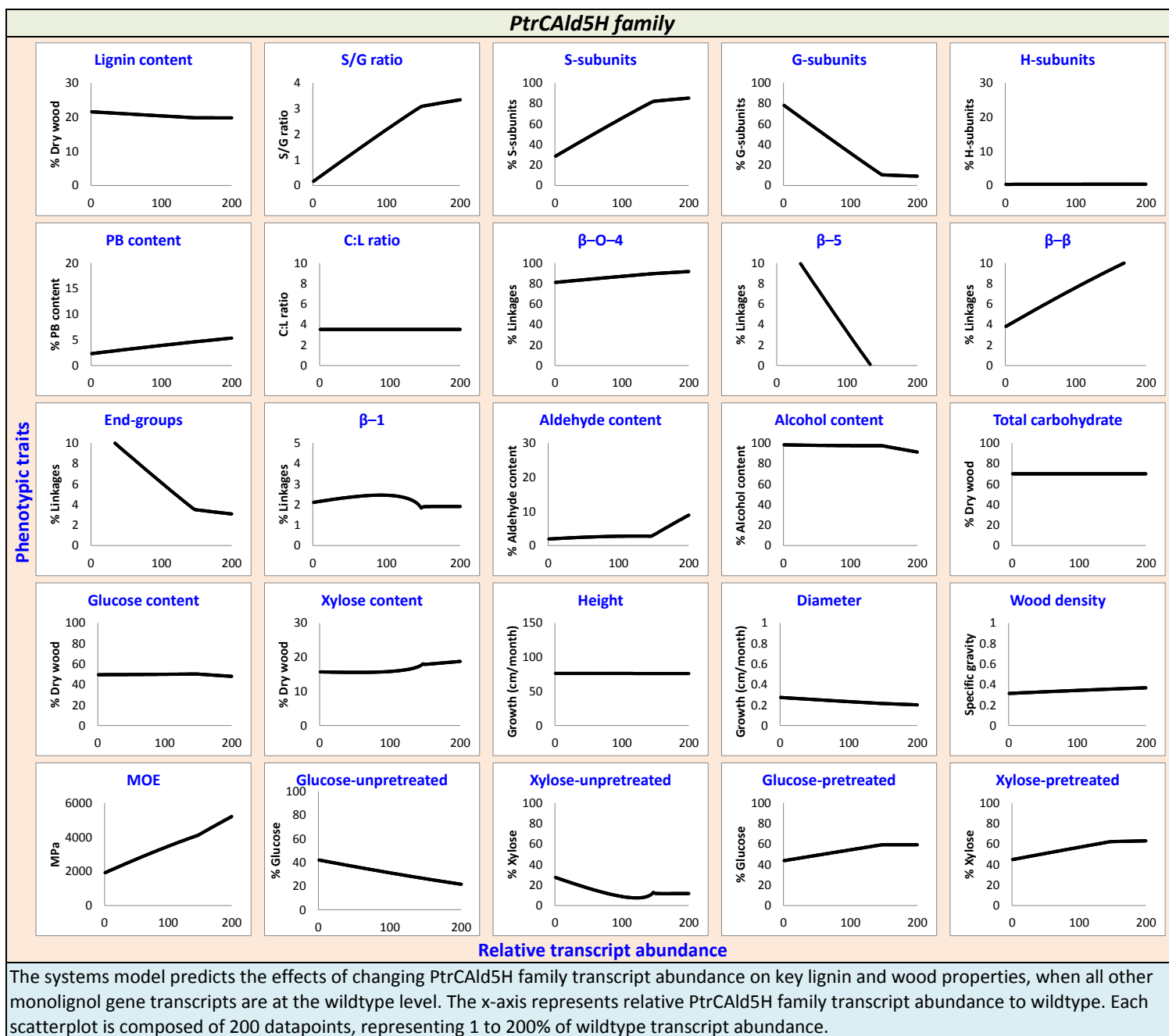

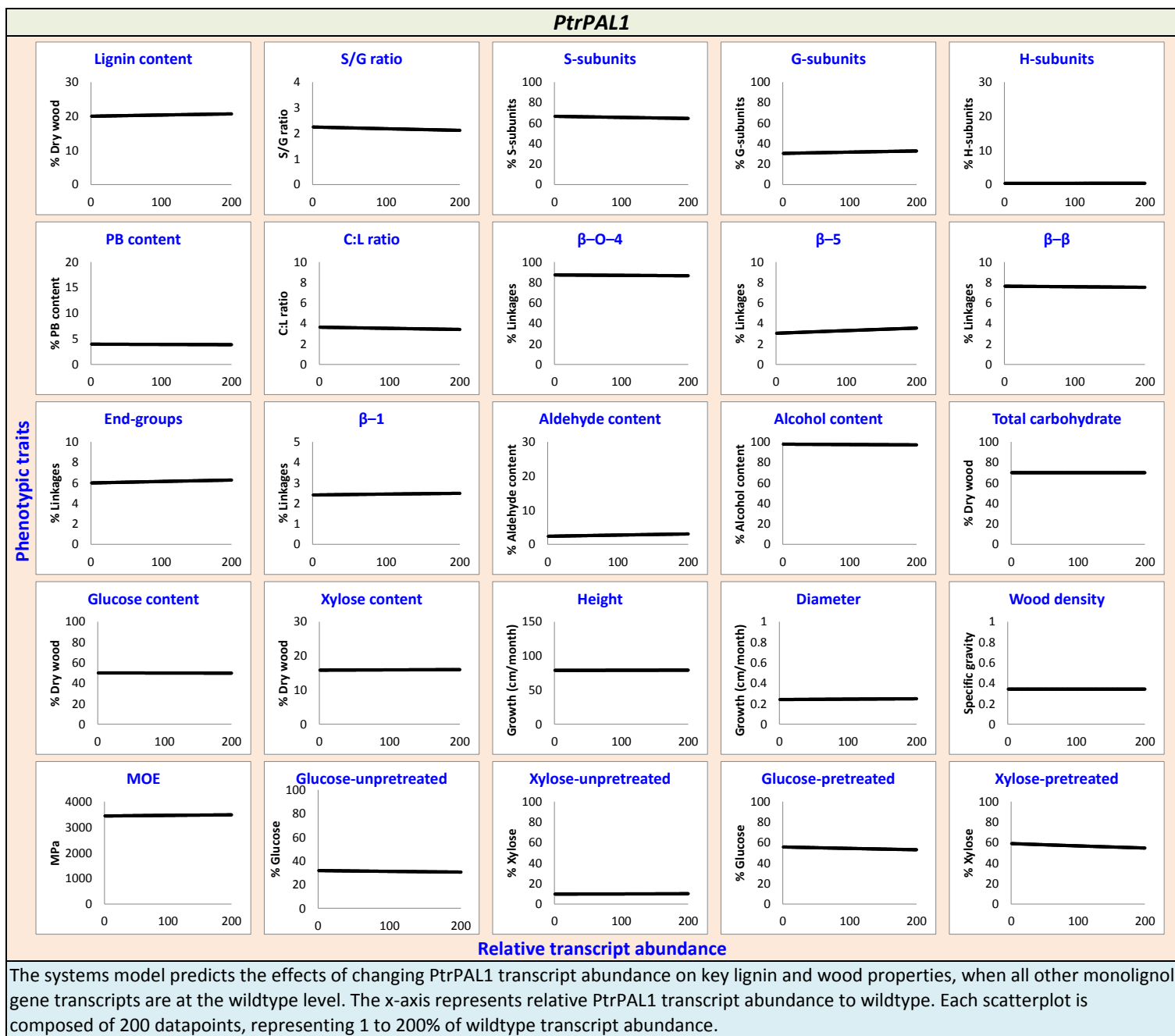

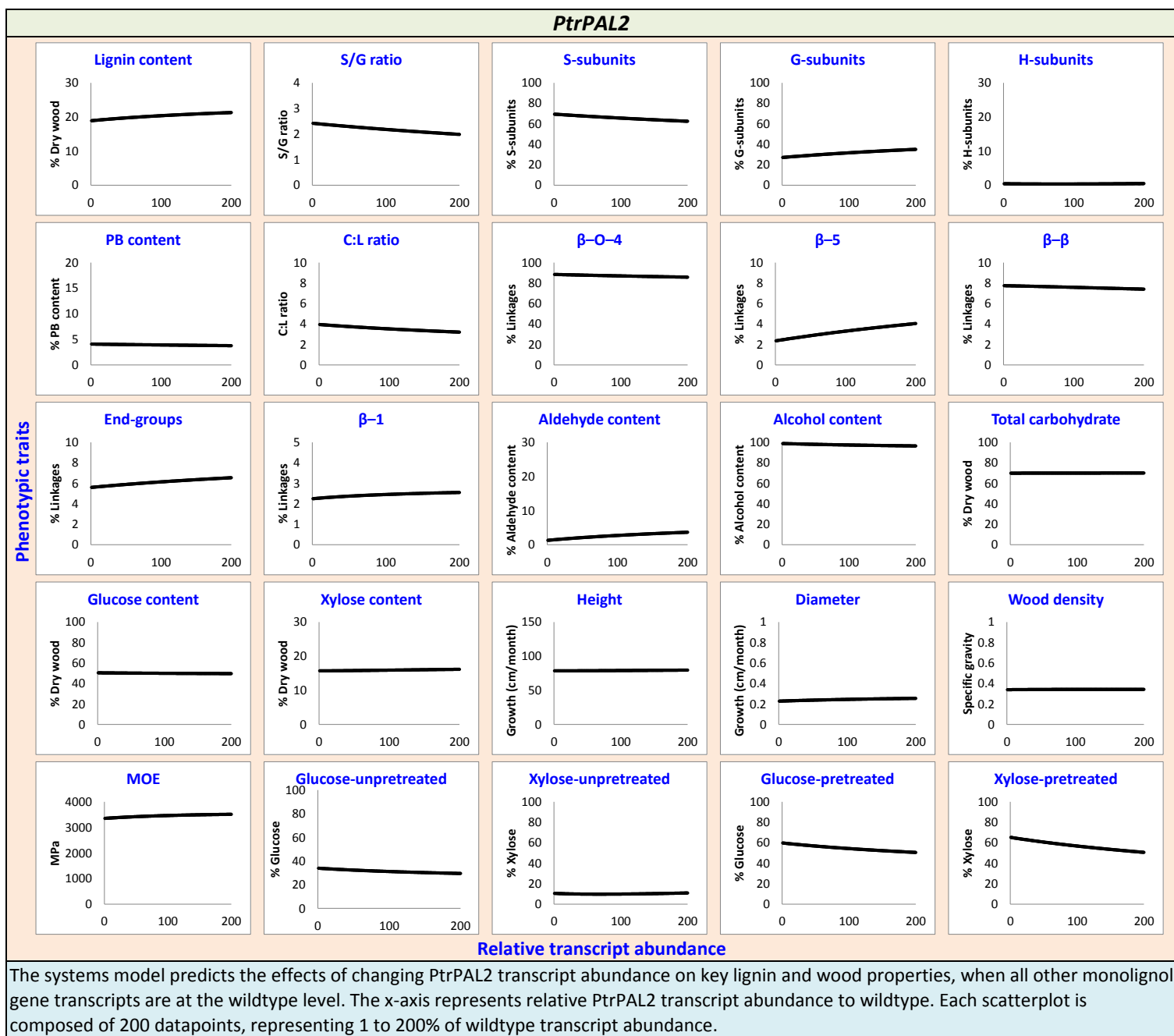

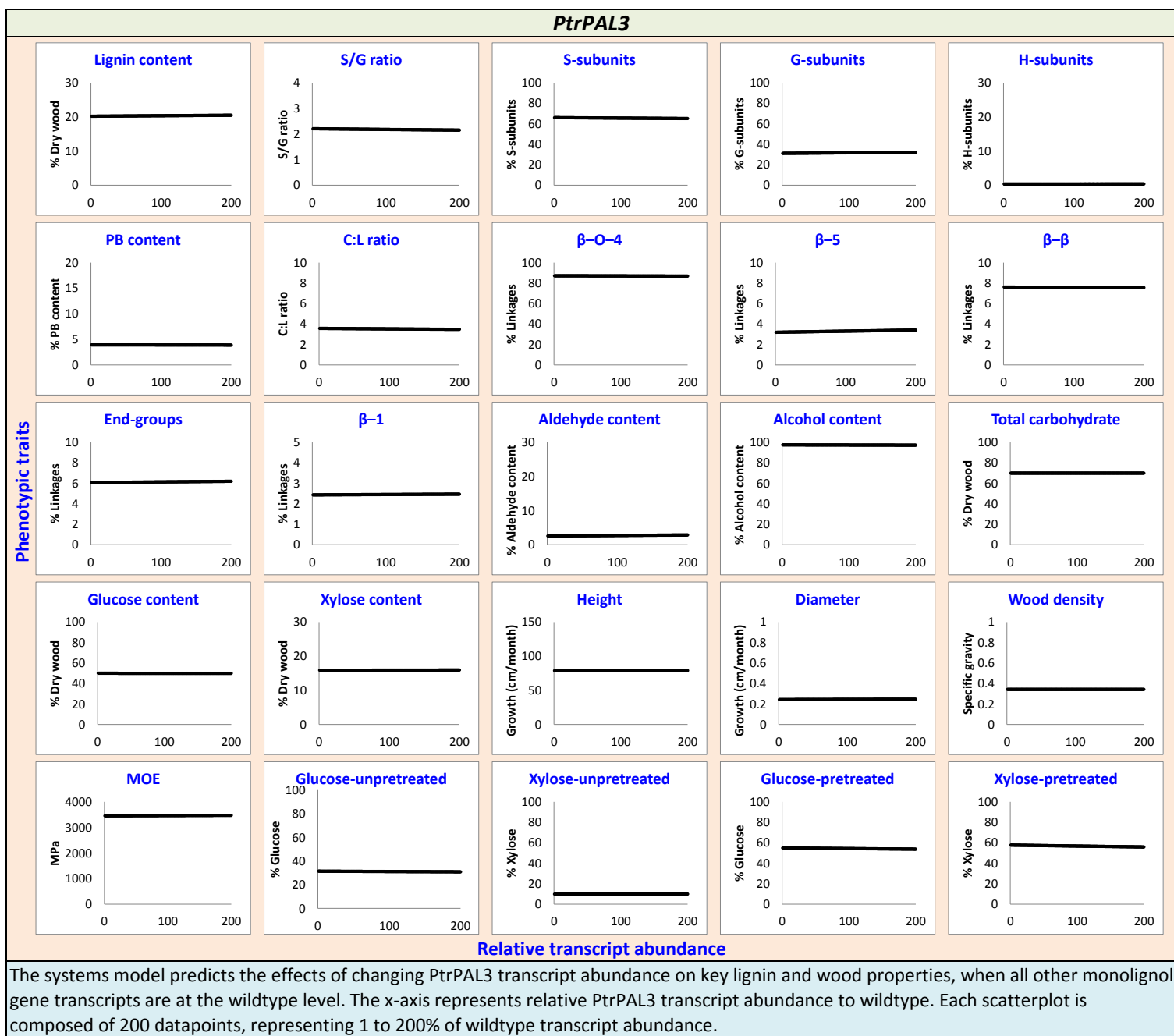

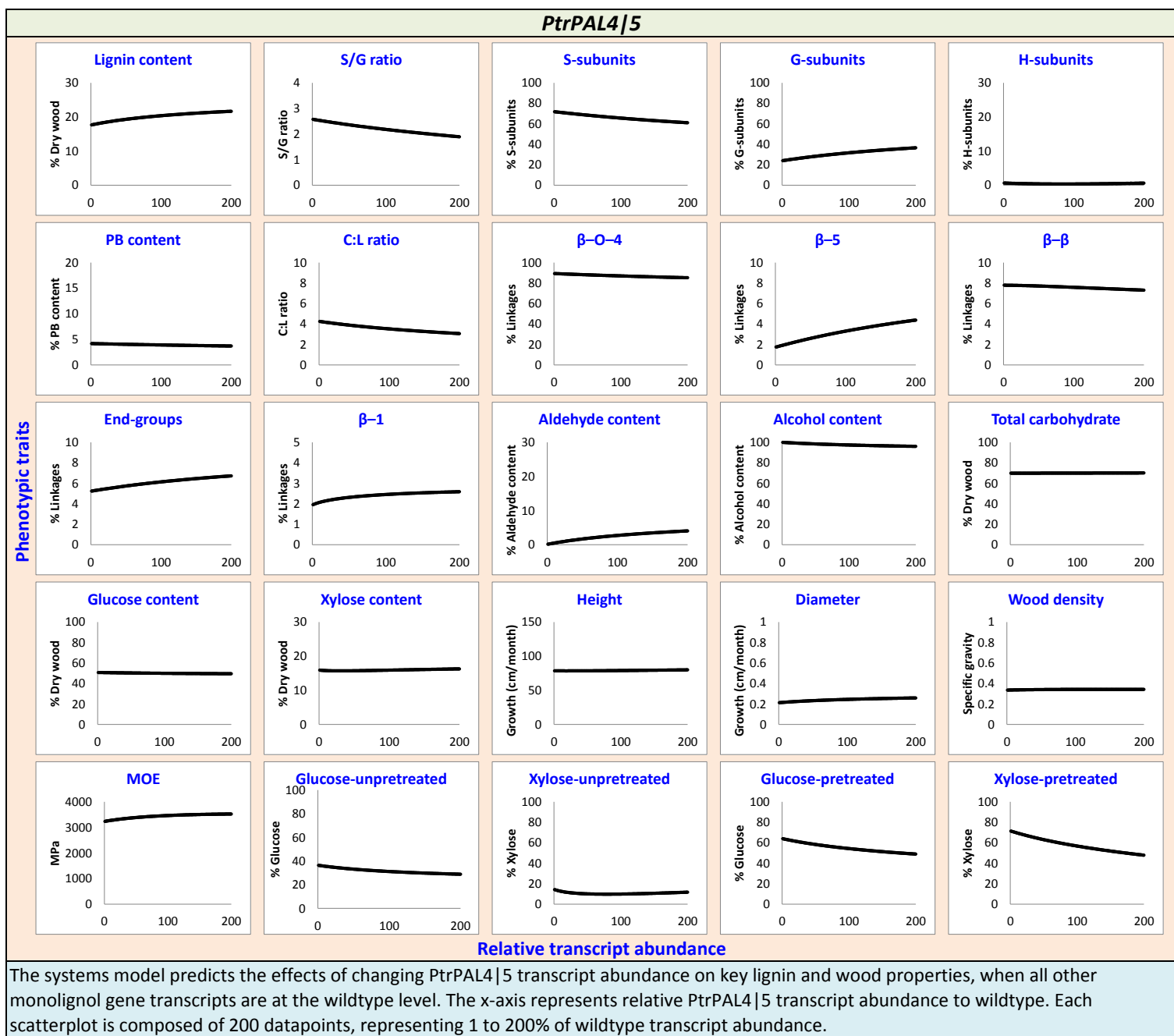

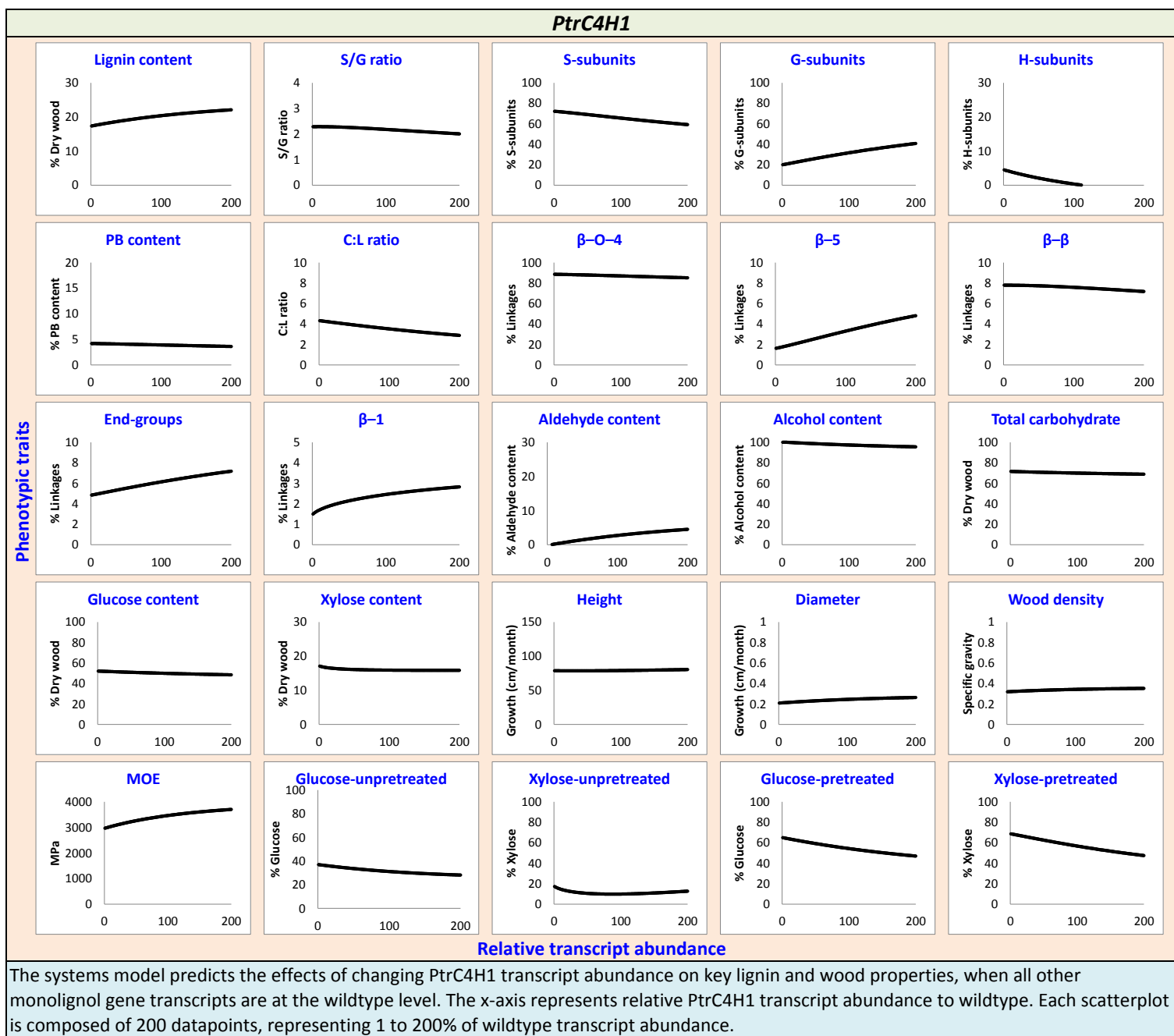

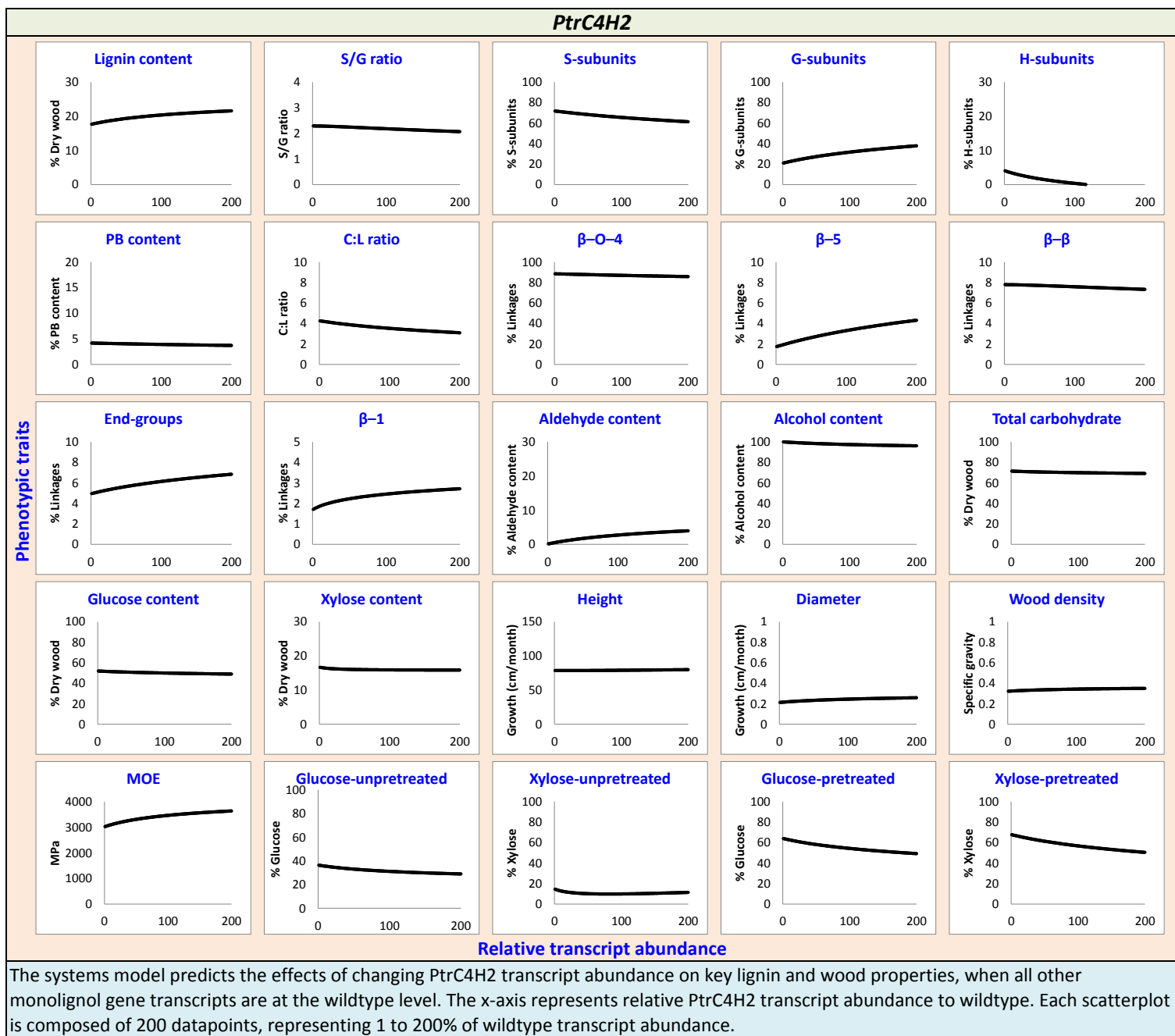

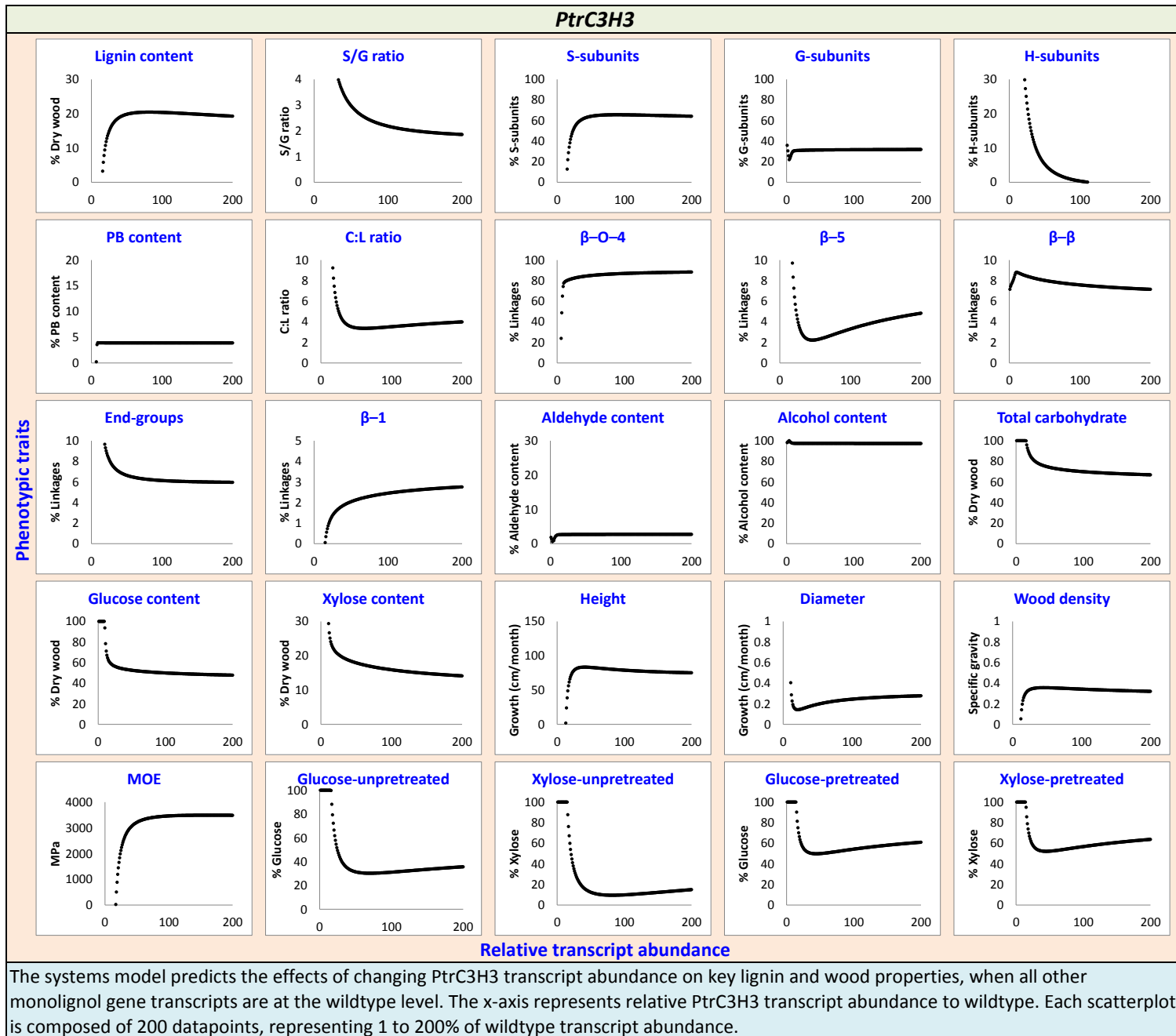

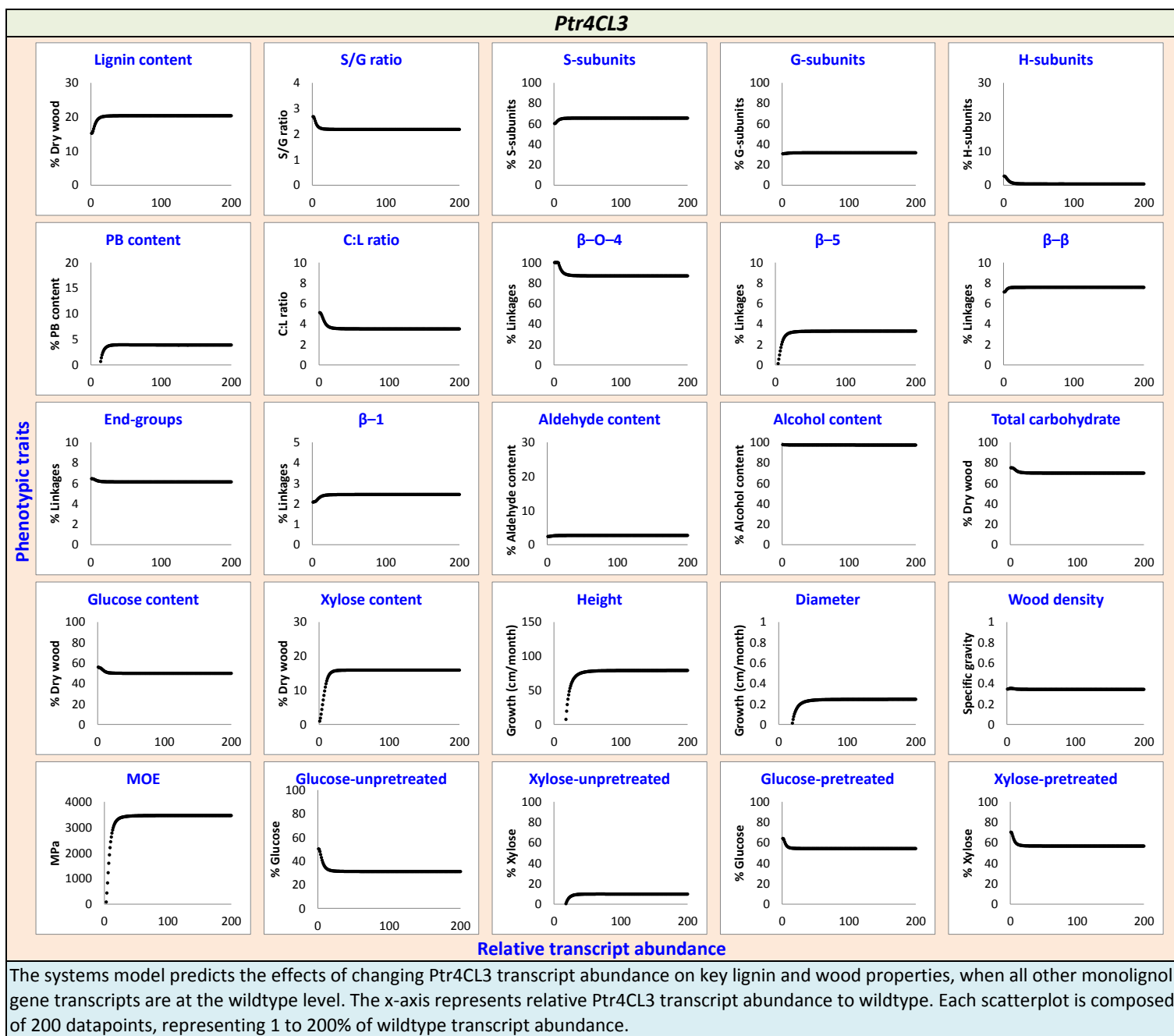

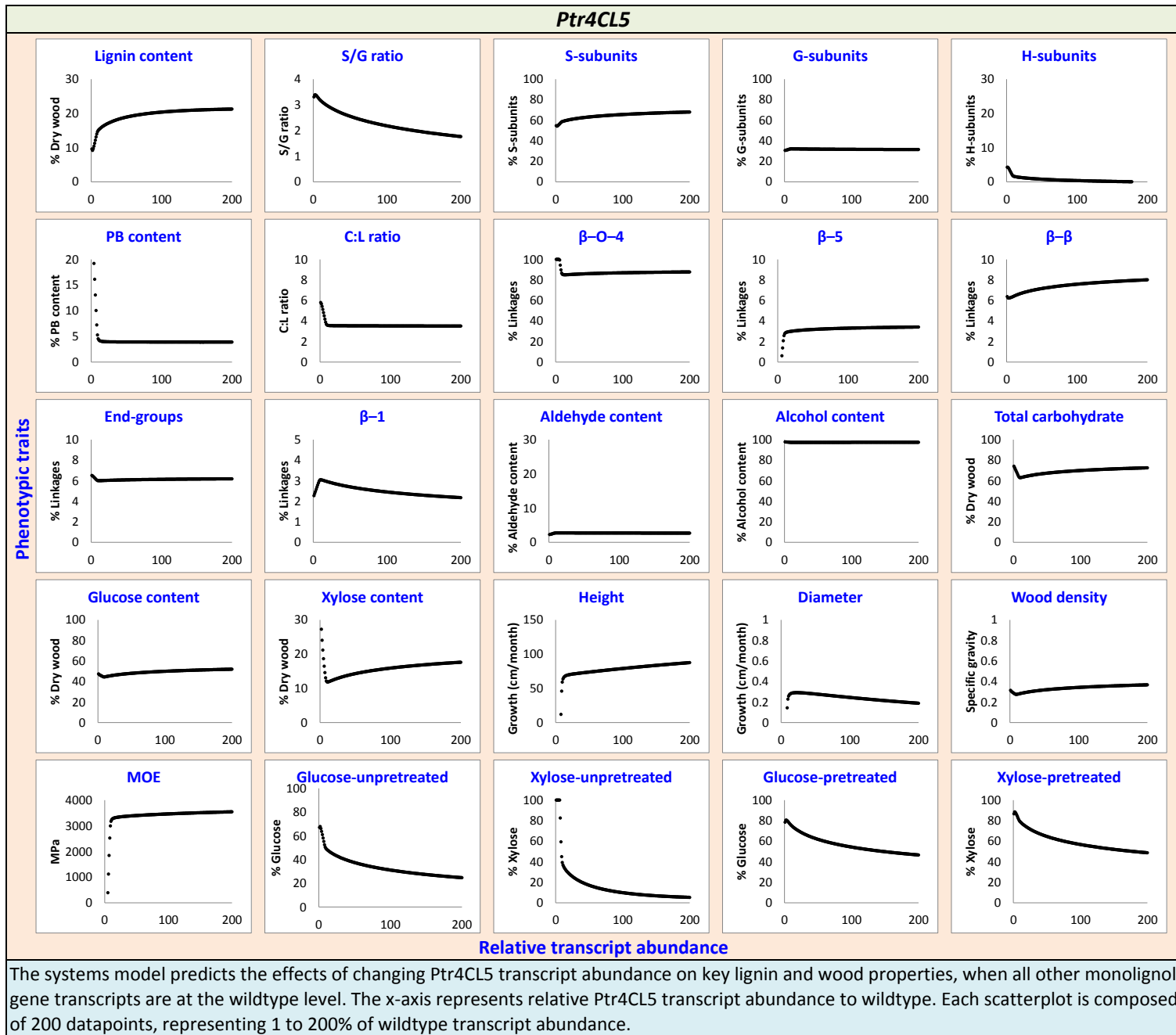

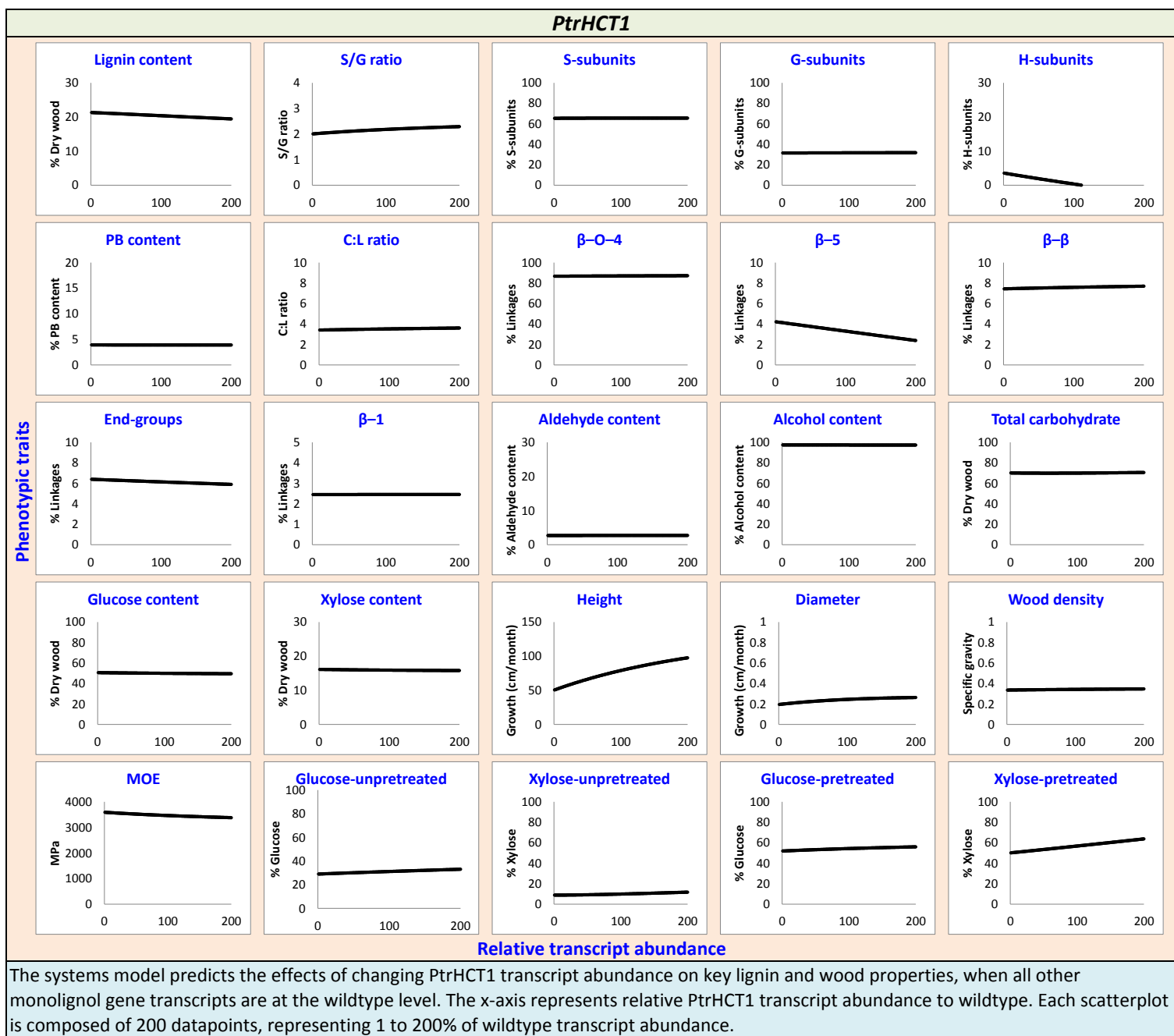

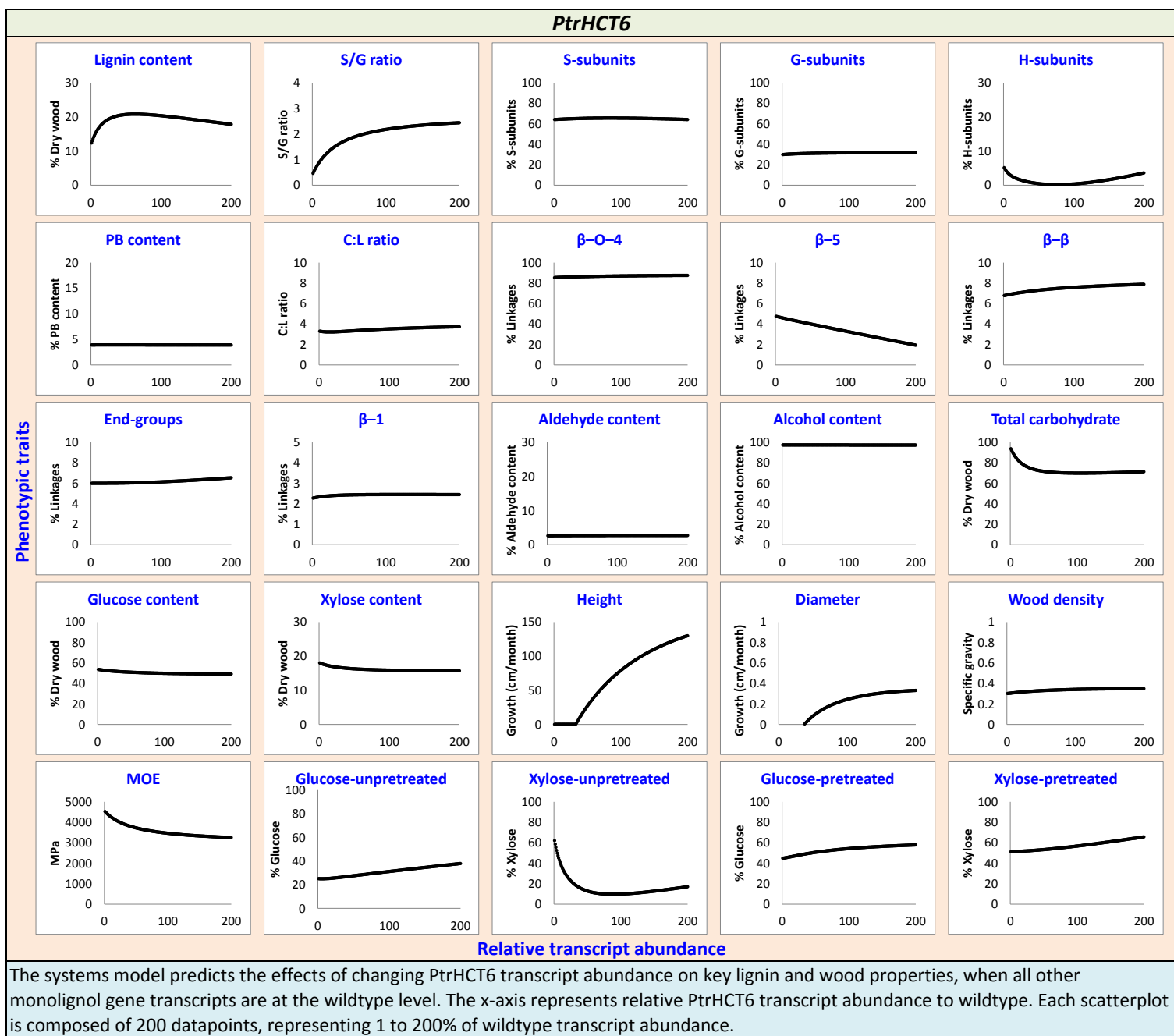

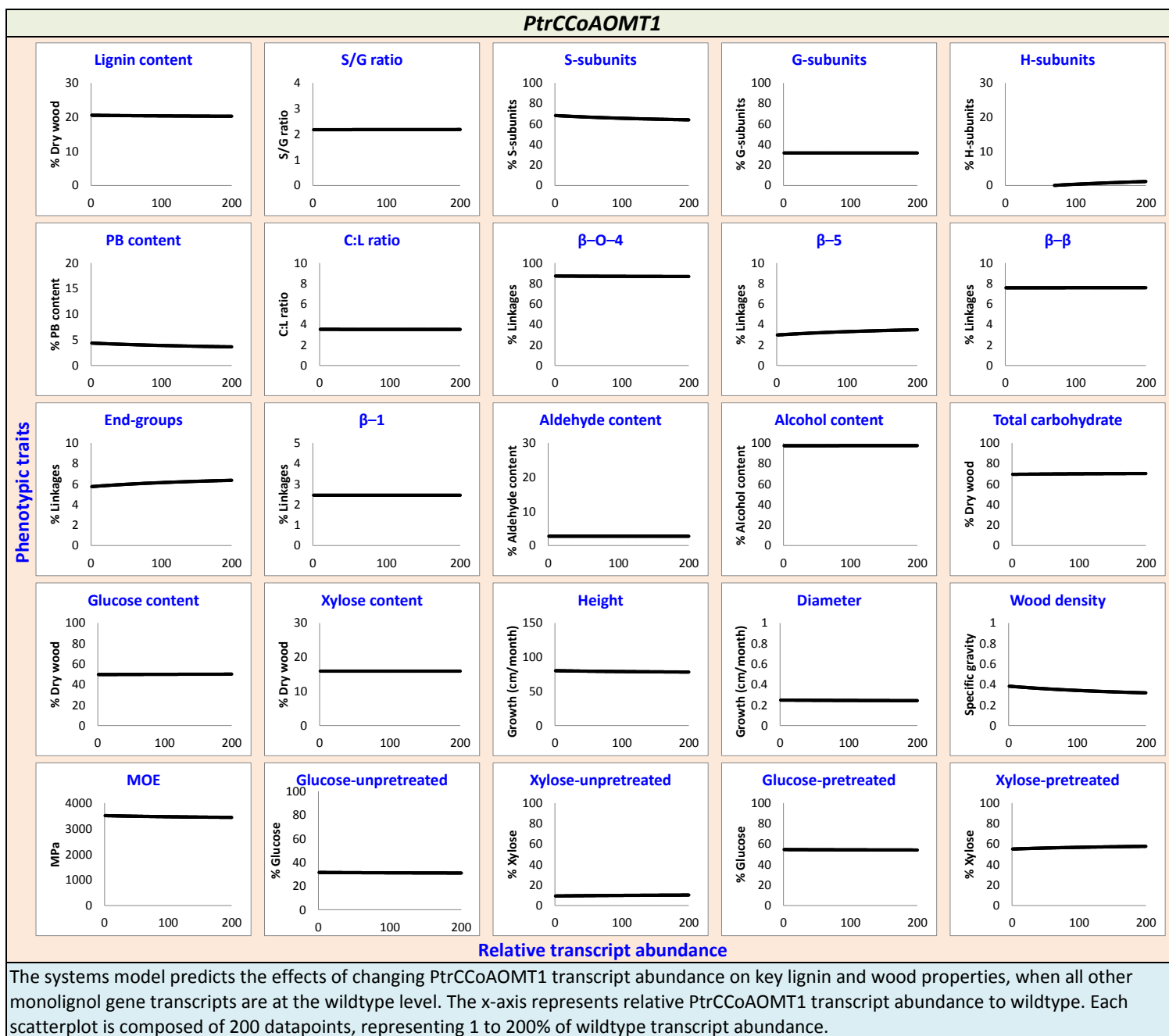

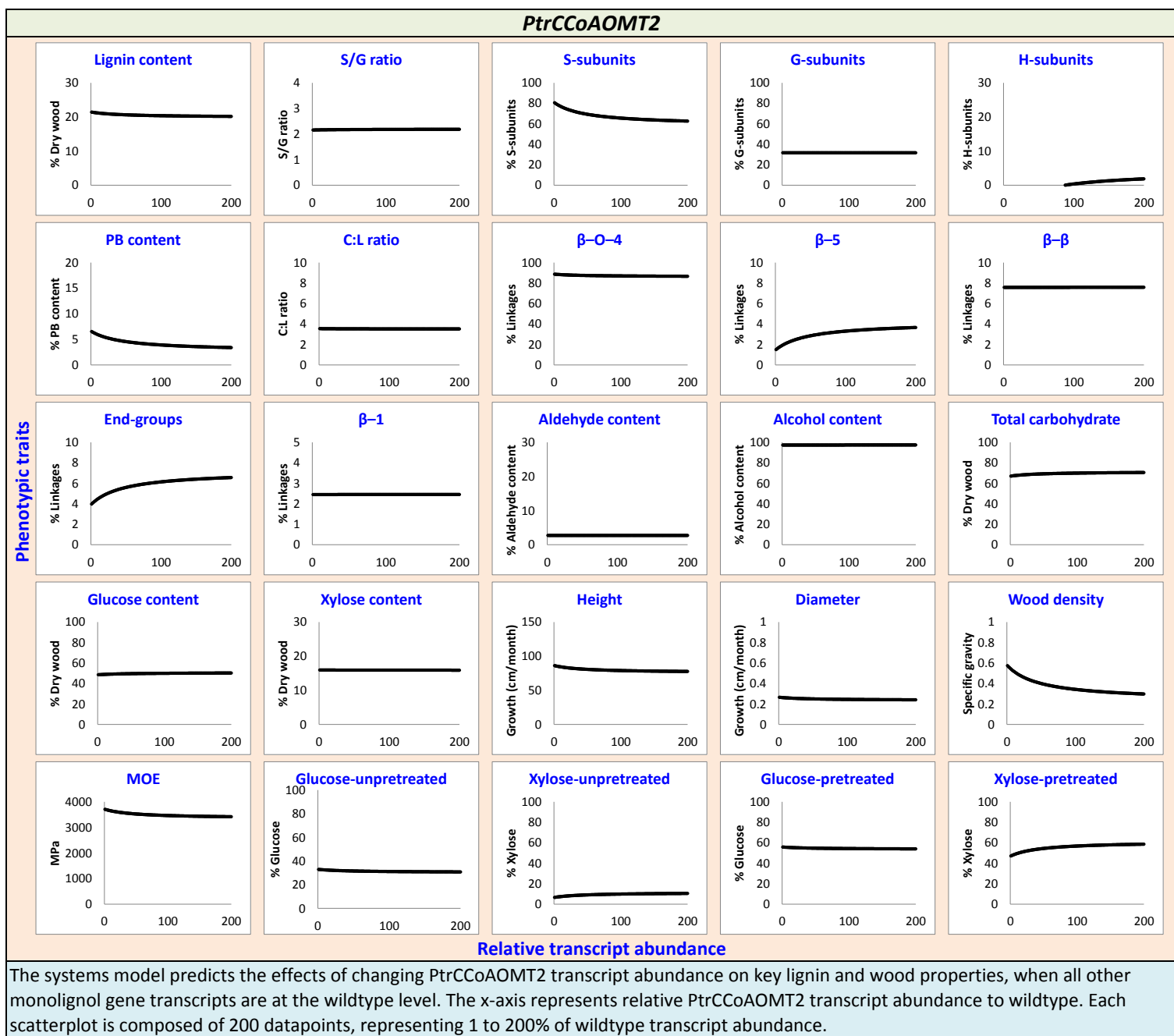

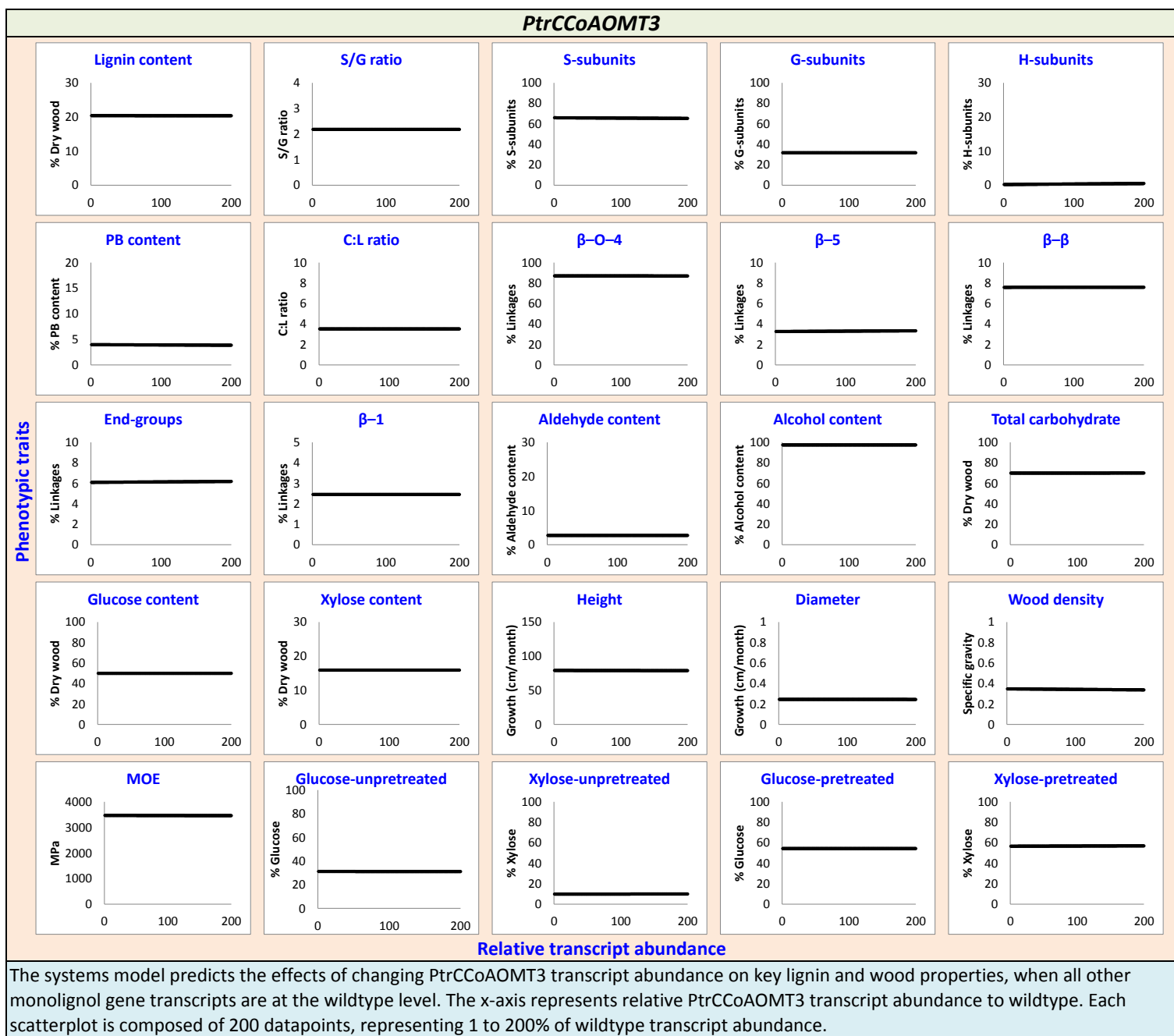

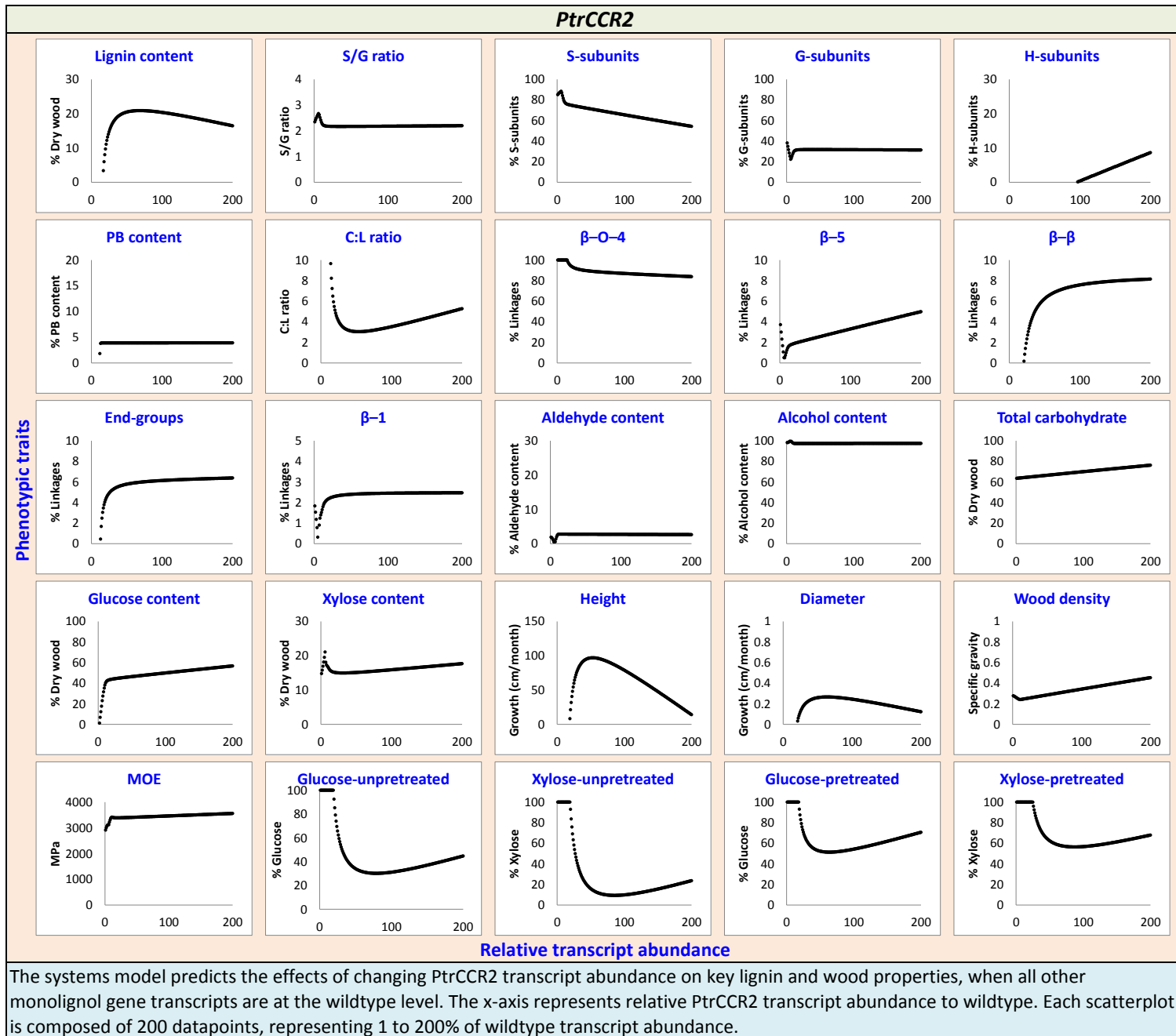

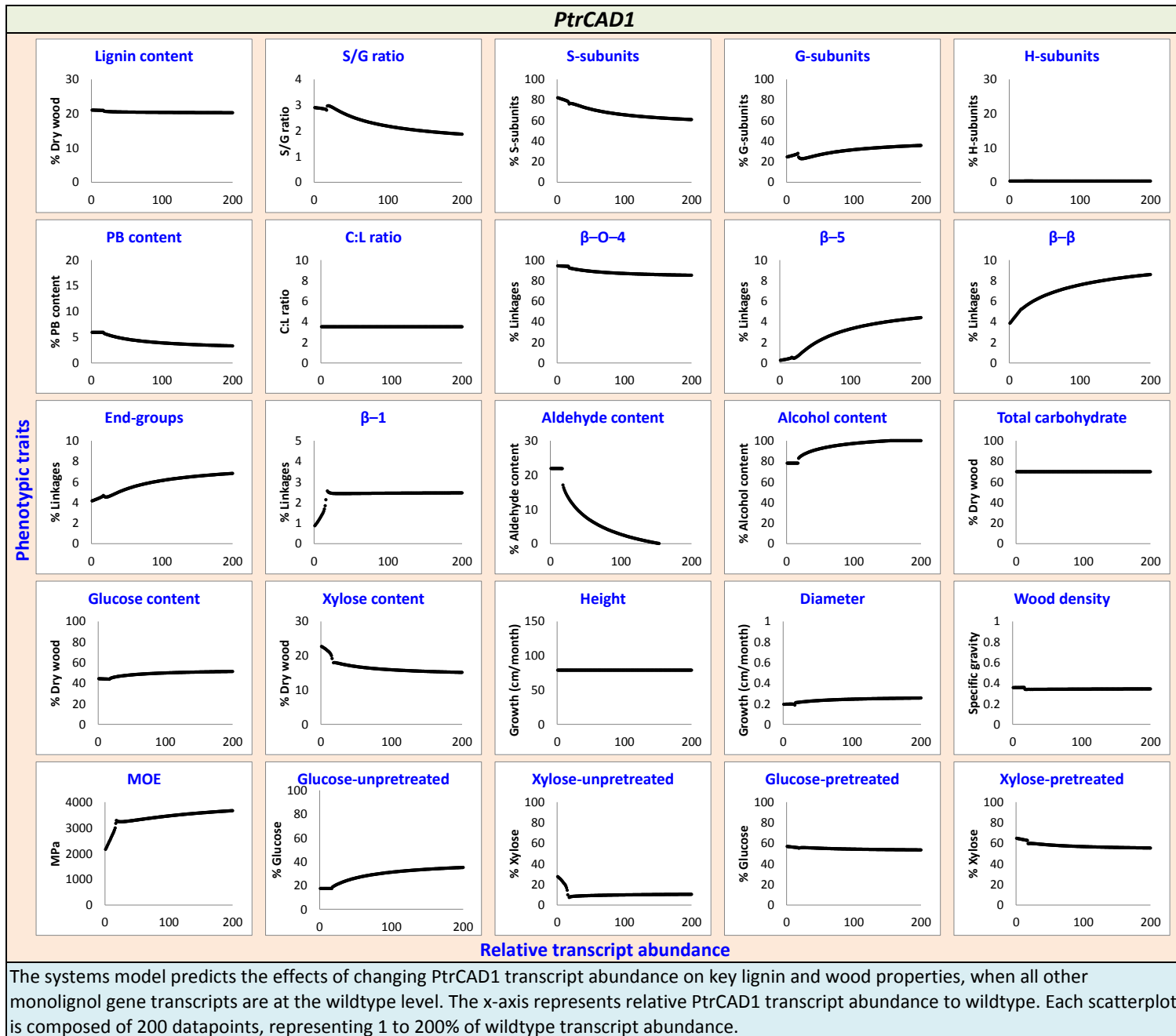

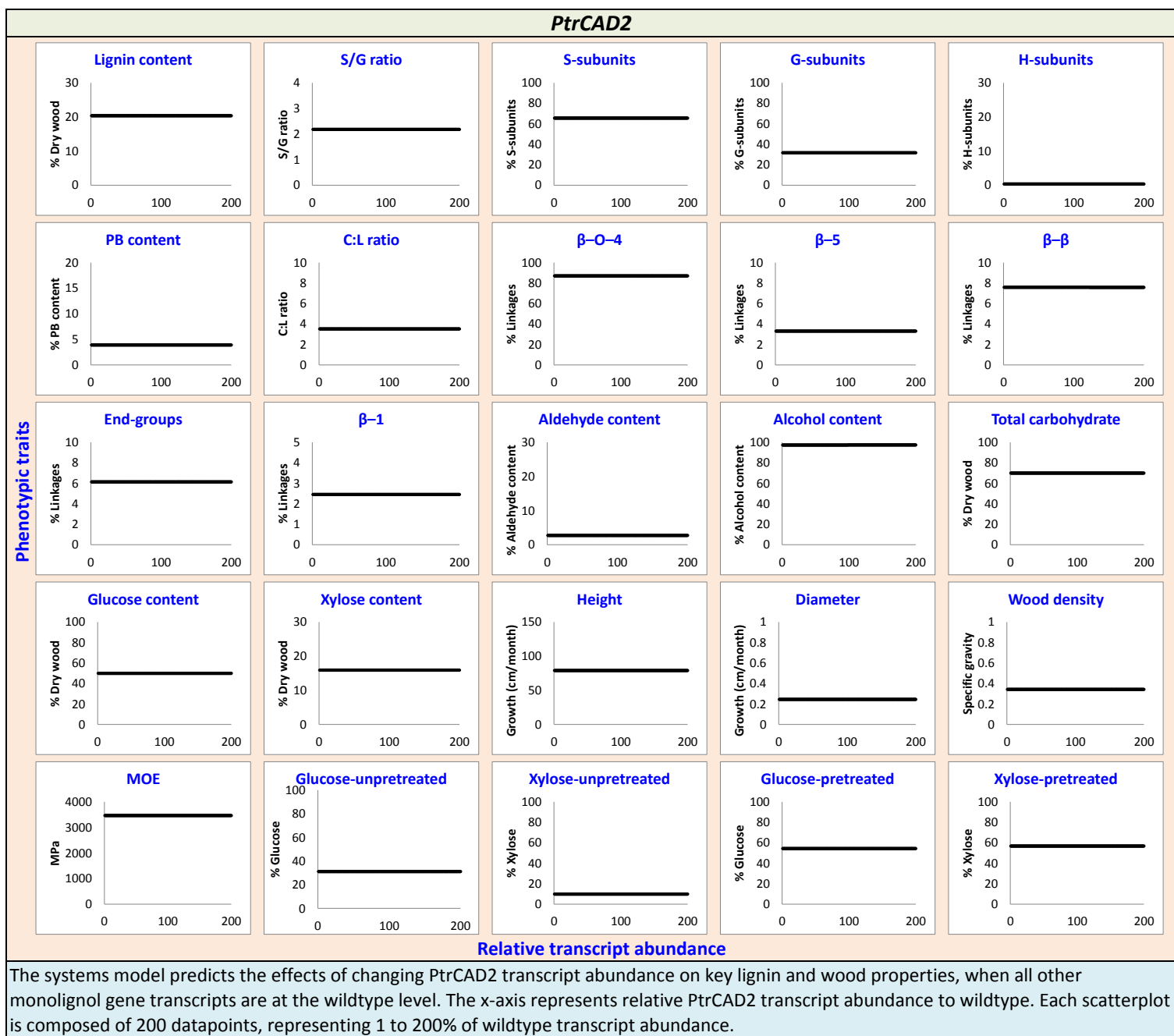

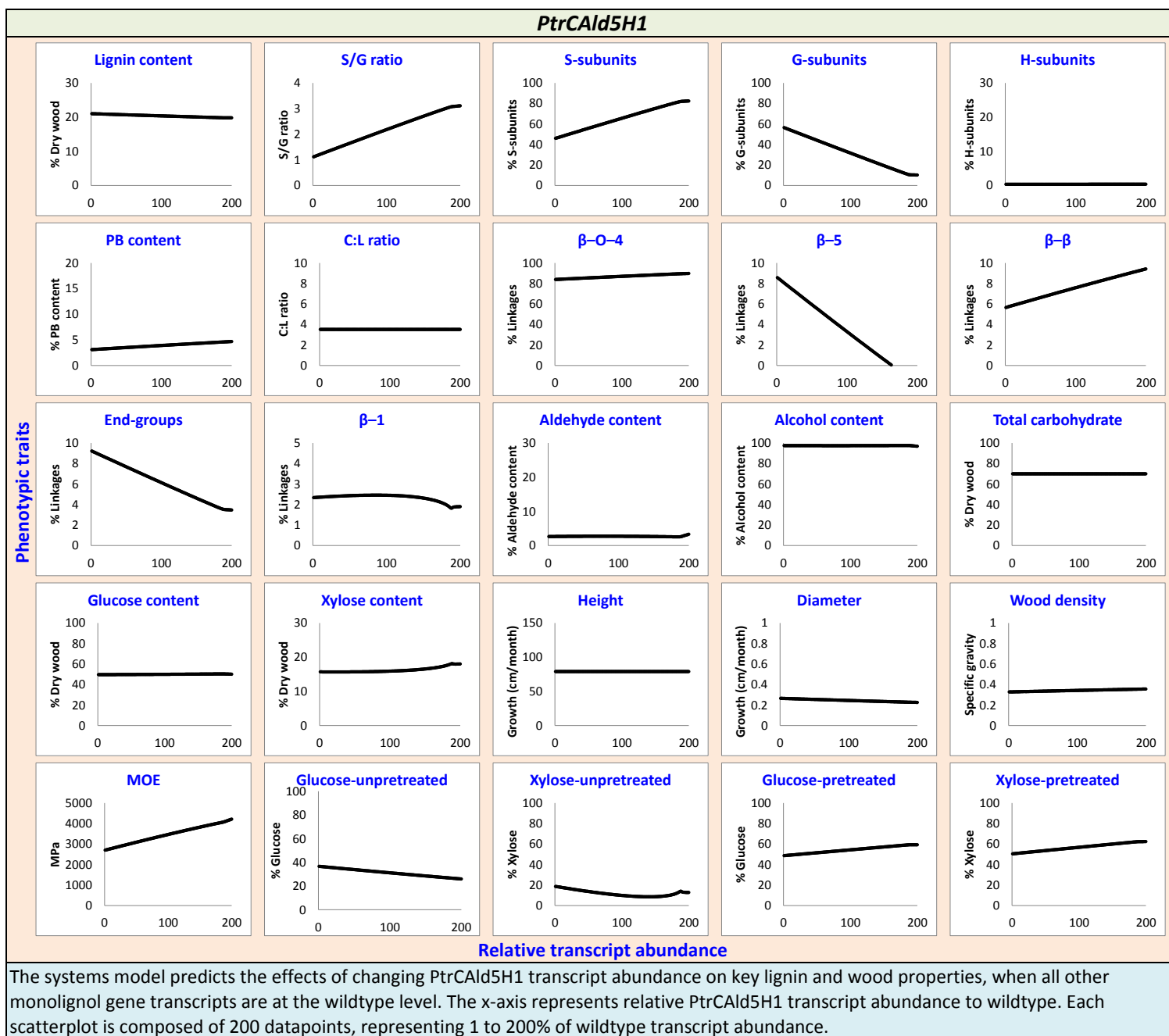

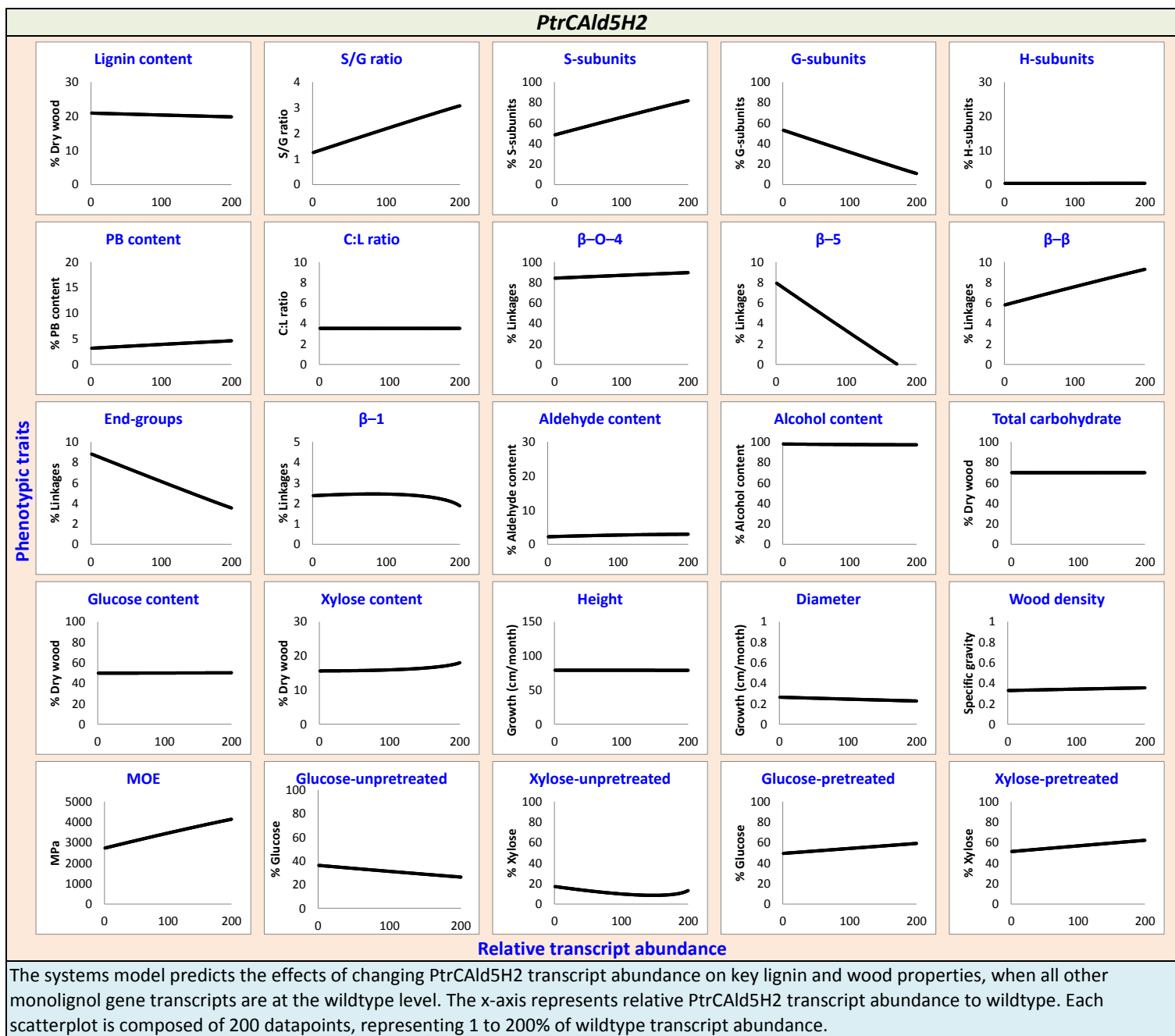

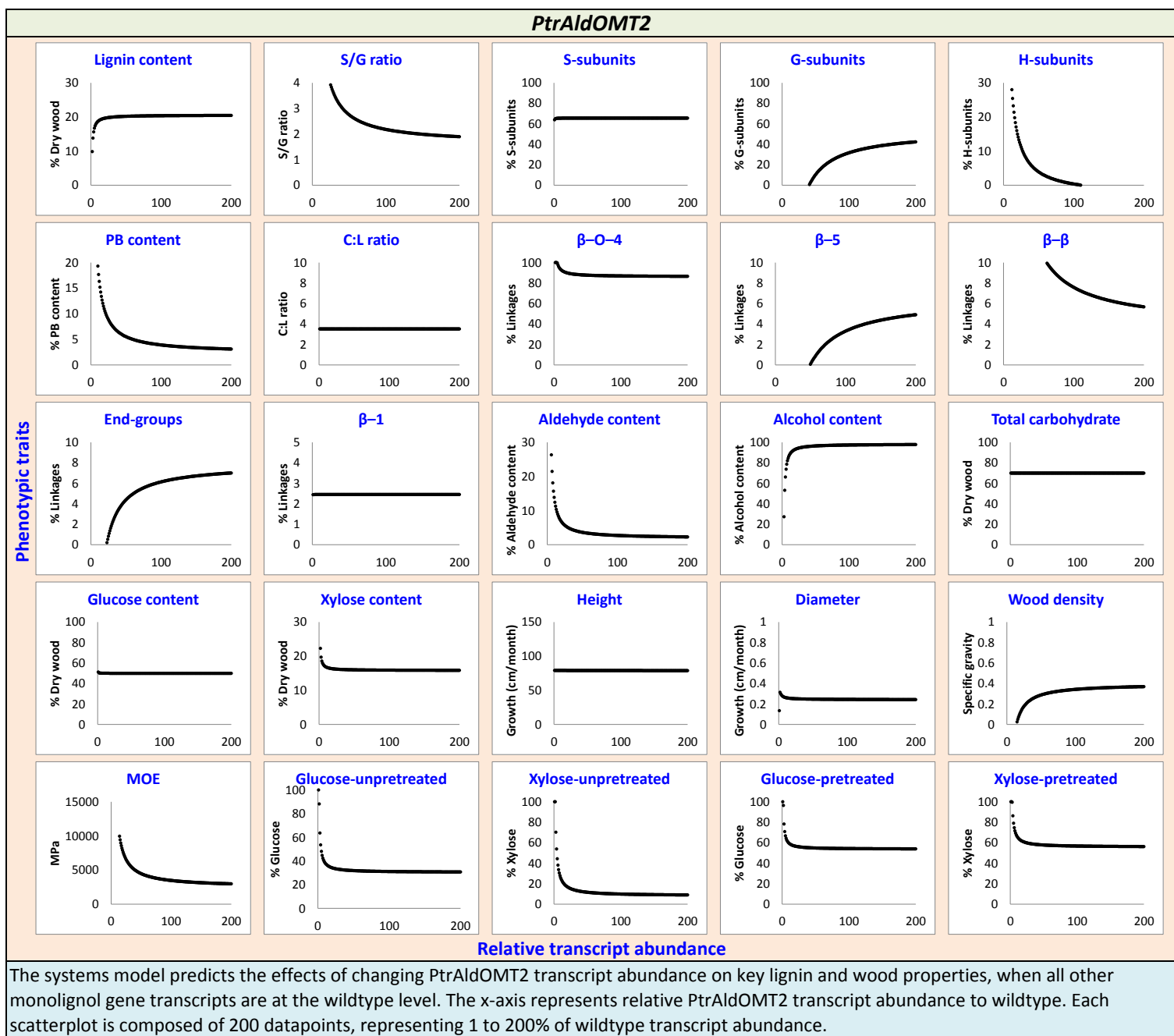

Supplement: Supplementary file 12 — Supplementary Data 10 [file 41467_2018_3863_MOESM12_ESM.pdf]
